# Elucidating the Mechanism of Metabolism of Cannabichromene by Human Cytochrome P450s

**DOI:** 10.1021/acs.jnatprod.3c00336

**Published:** 2024-03-13

**Authors:** Pritam Roy, Jonathan Maturano, Hale Hasdemir, Angel Lopez, Fengyun Xu, Judith Hellman, Emad Tajkhorshid, David Sarlah, Aditi Das

**Affiliations:** † School of Chemistry and Biochemistry, College of Sciences, and Parker H. Petit Institute for Bioengineering and Biosciences (IBB), Georgia Institute of Technology (GaTech), Atlanta, Georgia 30332, United States; ‡ Roger Adams Laboratory, Department of Chemistry, Cancer Center at Illinois, University of Illinois, Urbana, Illinois 61801, United States; § Theoretical and Computational Biophysics Group, NIH Center for Macromolecular Modeling and Visualization, Beckman Institute for Advanced Science and Technology, Department of Biochemistry, and Center for Biophysics and Quantitative Biology, University of Illinois at Urbana−Champaign, Urbana, Illinois 61801, United States; ∥ Judith Hellman Department of Anesthesia and Perioperative Care, University of California, San Francisco, California 94143, United States; ⊥ Department of Anesthesia and Perioperative Care, University of California, San Francisco, California 94143, United States

## Abstract

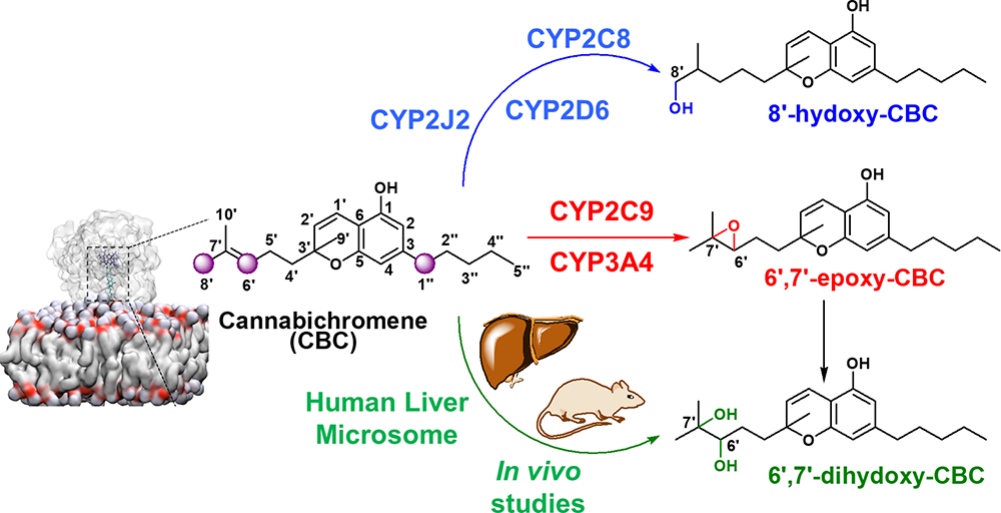

Cannabichromene (CBC) is a nonpsychoactive phytocannabinoid well-known for its wide-ranging health advantages. However, there is limited knowledge regarding its human metabolism following CBC consumption. This research aimed to explore the metabolic pathways of CBC by various human liver cytochrome P450 (CYP) enzymes and support the outcomes using *in vivo* data from mice. The results unveiled two principal CBC metabolites generated by CYPs: 8′-hydroxy-CBC and 6′,7′-epoxy-CBC, along with a minor quantity of 1″-hydroxy-CBC. Notably, among the examined CYPs, CYP2C9 demonstrated the highest efficiency in producing these metabolites. Moreover, through a molecular dynamics simulation spanning 1 μs, it was observed that CBC attains stability at the active site of CYP2J2 by forming hydrogen bonds with I487 and N379, facilitated by water molecules, which specifically promotes the hydroxy metabolite’s formation. Additionally, the presence of cytochrome P450 reductase (CPR) amplified CBC’s binding affinity to CYPs, particularly with CYP2C8 and CYP3A4. Furthermore, the metabolites derived from CBC reduced cytokine levels, such as IL6 and NO, by approximately 50% in microglia cells. This investigation offers valuable insights into the biotransformation of CBC, underscoring the physiological importance and the potential significance of these metabolites.

Cannabis plants are recognized for their diverse medicinal attributes.^1^ The primary constituents of cannabis plants, Δ9-tetrahydrocannabinol (THC) and cannabidiol (CBD), are plentiful and extensively researched. Cannabis extracts contain over 120 cannabinoids and terpenes. Lately, lesser-known cannabinoids such as cannabigerol (CBG) and cannabichromene (CBC) have gained recognition for their promising pain-relief properties and nonpsychoactive attributes. However, the limited presence of CBC in cannabis has hindered comprehensive research into its properties.^2,3^ Radioligand assays have shown that CBC has a very low binding affinity toward cannabinoid receptor 1 (CBR1) [*K*_i_ ∼ 713 nM] compared to THC [*K*_i_ ∼ 35 nM].^4^ As a result, CBC does not induce psychoactive effects since it does not strongly interact with CBR1. Recent research indicates that CBC is more effective as an agonist for cannabinoid receptor 2 (CB2) with a *K*_i_ value of approximately 100 nM, implying its potential involvement in inflammation reduction.^5^ CBC also engages with transient receptor potential (TRP) channels and various pain receptors. It acts as both an activator and desensitizer of transient receptor potential ankyrin 1-type (TRPA1) channels.^6,7^ Furthermore, CBC diminishes the release of nitric oxide induced by LPS in macrophages, akin to TRPA1 agonists such as carvacrol and cinnamaldehyde.^8^ CBC exhibits more potent agonism toward TRPV1 and TRPV2 (<10 μM for both receptors) than CBD (∼40 μM for both receptors) or CBG (∼33 μM for TRPV1 and ∼73 μM for TRPV2).^9^ This suggests that CBC can act as an antinociceptive agent. *In vitro* studies have shown that CBC inhibits the differentiation of adult neural progenitor cells into astroglia and increases the viability of the neural progenitor cells. This opens up avenues for treating neuroinflammatory diseases using CBC.^10^ In addition, CBC has displayed anti-inflammatory, analgesic, and antidepressant-like activities in rodent models.^11−13^

Patients who consumed a CBD oil, which is a mixture of several cannabinoids, displayed detectable levels of CBC in their plasma (∼60 ng/mL) and urine (∼94 ng/mL) samples.^14^ Metabolic studies have shown that CBC absorption from human plasma is higher than that of THC and CBD when all three cannabinoids are administered together.^2^ Pharmacokinetic (PK) investigations have revealed that when smoking cannabis cigarettes, THC undergoes rapid oxidation, resulting in the formation of 11-OH-THC and THC-COOH.^15^ In a recent study, we showed that the blood plasma concentration of cannabigerol (CBG), a nonpsychoactive phytocannabinoid, significantly decreases within 2 h after its parenteral administration in female mice.^16^ Microsome-based CBC metabolism studies on various rodent species have previously identified hydroxy and epoxide products.^17^ Through GC/MS analysis, it was established that the major metabolite was 8′-hydroxy CBC (**2**), and the minor metabolite was 1″-hydroxy CBC (**10**). Intriguingly, the research also revealed that in mice, the primary metabolite was 6′,7′-epoxy CBC (**4**), whereas in rabbits, 6′,7′-dihydroxy CBC (**5**) was identified as the primary metabolite. It is noteworthy that these studies did not utilize synthesized authentic standards.

Within a species, it has been demonstrated that sexual dimorphism can influence metabolic pathways. For instance, 11-hydroxy-THC, a prominent metabolite of THC, is found in higher abundance in female mice.^18^ This is due to different levels of expression of cytochrome P450 (CYP) enzymes in males and females, which are primarily involved in phase I drug metabolism.^19^ CYPs constitute a family of enzymes capable of catalyzing oxidative biotransformation of most drugs and xenobiotics^20^ making them particularly relevant to clinical pharmacology.^21^ These enzymes are involved in the biosynthesis of steroid hormones, prostaglandins, and bile acids.^22,23^ CYP3A4 is a major CYP expressed in the human liver, along with CYP3A5, and it is responsible for the metabolism of ∼50% of prescribed drugs.^24^ Another member of the CYP2 family, CYP2C9,^25^ is associated with the metabolism of the anticoagulant warfarin, the anticonvulsants phenytoin and valproic acid, and the angiotensin receptor blocker candesartan.^26^ CYP2C9 is predominantly associated with the metabolism of cannabinoids such as THC^27^ and CBG.^16^ CYPs have large catalytic pockets which can accommodate more than one substrate at a time. Kinetic studies have demonstrated that cannabinoids can be metabolized into various oxidative metabolites. Interestingly, certain cannabinoids have been observed to serve as inhibitors of CYP enzymes. As an illustration, we recently demonstrated that THC and CBD act as inhibitors of anandamide (AEA) metabolism by CYP2D6.^28^ Hence, it becomes imperative to explore the metabolism of CBC by various human CYPs to gain a comprehensive understanding of its pharmacological effects. Human CYPs operate in conjunction with the cytochrome P450 reductase (CPR) enzyme. The electron transfer process is initiated by nicotinamide adenine dinucleotide phosphate (NADPH), passing through flavin adenine dinucleotide (FAD) to flavin mononucleotide (FMN) within CPR, ultimately facilitating the oxidation of substrates by the heme group in CYPs.^29,30^ Studies have indicated that the binding of particular ligands to CPR can modify the way it interacts with CYPs, subsequently impacting the overall metabolism process.^31^

In this study, we undertook a comprehensive investigation of CBC metabolism by CYPs (Figure 1). We employed mass spectrometry techniques for precise metabolite identification, validating the results with synthesized authentic standards. Molecular dynamics simulations were employed to enhance our comprehension of the product distribution stemming from CBC metabolism. Additionally, we delved into the influence of CPR on the modulation of CYP–CBC interactions and the consequent metabolic pathways. Our investigation establishes that the resultant metabolites manifest anti-inflammatory properties in lipopolysaccharide (LPS)-stimulated microglial cells.

**Figure 1:**
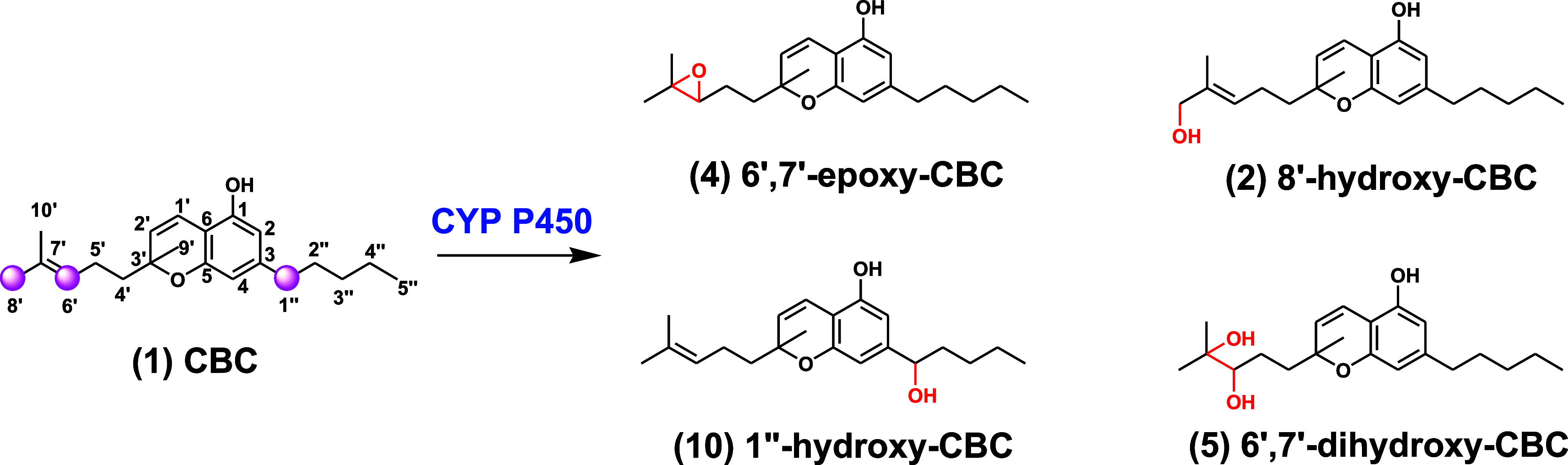
CBC and its metabolites: CBC (**1**) undergoes oxidation in the presence of CYP P450 to give 6′,7′-epoxy-CBC (**4**), 8′-hydroxy-CBC (**2**), 1″-hydroxy- CBC (**10**), and 6′,7′-dihydroxy-CBC (**5**). Please use an updated figure 1 [with better resolution] which is attached as ChemDraw file as well as in MS Word file

## Results and Discussion

### Synthesis of CBC, Its Metabolites, and Analogues

**Scheme 1:**
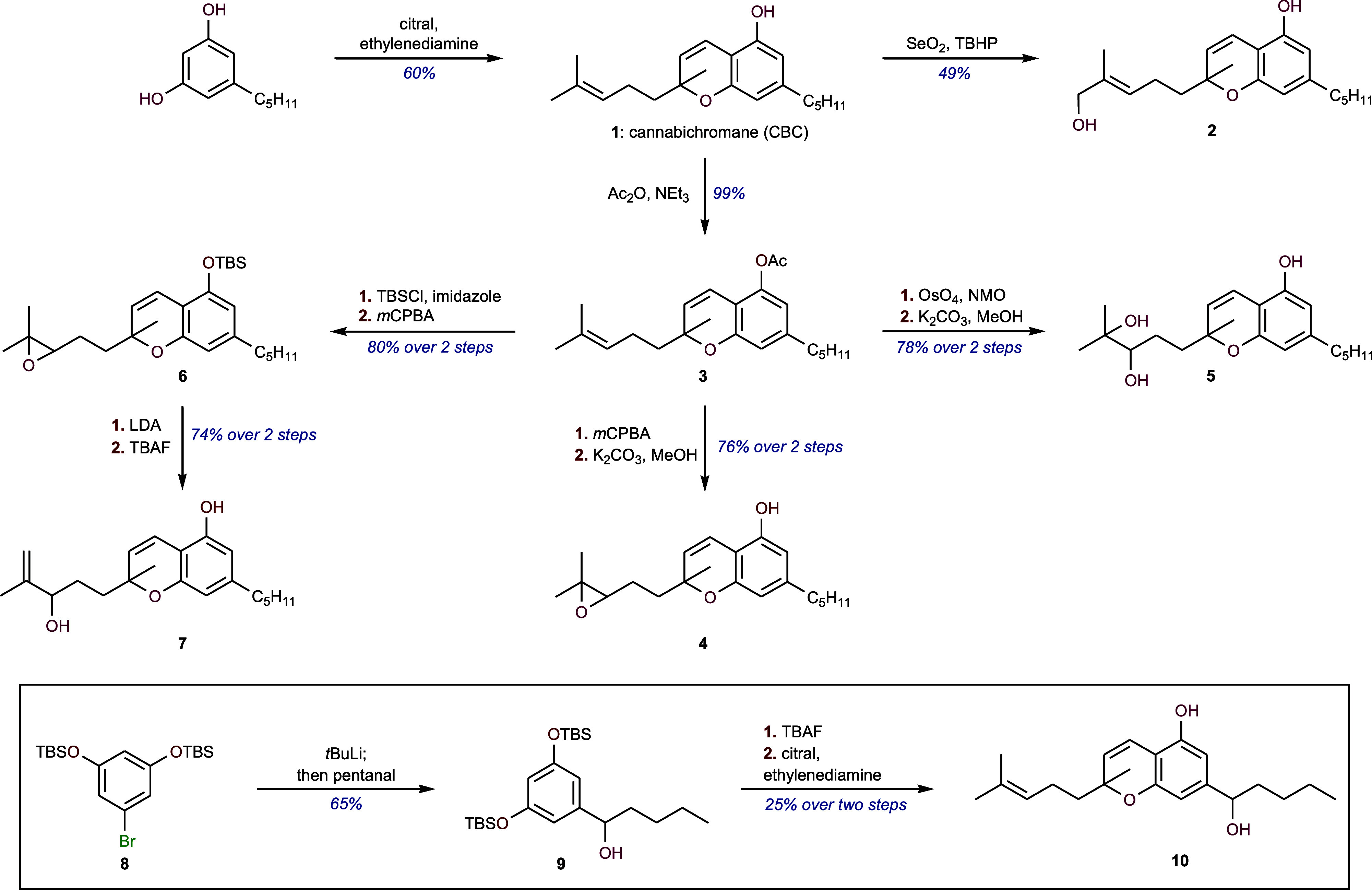
Synthesis of CBC and Its Metabolites Details of the synthesis are present in the Supporting Information.

Beginning from commercially available citral and olivetol, CBC (**1**) was prepared on a multigram scale according to Lee’s reported procedure.^32^ In order to secure different oxidation products, several protocols were explored to obtain desired chemoselectivity. For example, allylic oxidation could be achieved selectively at the C-8′ position by directly subjecting CBC (**1**) to SeO_2_,^33^ affording 8′-hydroxy-CBC (**2**) in appreciable yield. On the other hand, all other oxidations involving CBC required protection as noticeable degradations were observed when the free phenol was used. Thus, acetate protection of CBC (**1**) afforded intermediate **3** in quantitative yield. Upon dihydroxylation^34^ of this intermediate, 6′,7′-diol was produced, and subsequent deacetylation gave 6′,7′-dihydroxy-CBC (**5**). Acetylated compound **3** could also undergo epoxidation at the same site using *m*CPBA, affording 6′,7′-epoxy-CBC (**4**) after base-mediated deacetylation. While isomerization of these epoxides proved difficult, a similarly obtained *tert*-butyldimethylsilyl (TBS)-protected epoxide **6** was amenable to base-induced isomerization conditions with LDA, delivering 7′,10′-ene-6′-hydroxy-CBC (**7**) after deprotection with tetra-*n*-butylammonium fluoride (TBAF). Access to oxidation at the C-1″-benzylic position from established intermediates proved challenging using several known oxidation conditions. Consequently, we introduced this oxidation during the early stages of synthesis by preparing the corresponding 1″-hydroxy-olivetol derivative. Thus, lithiated compound **8** (reaction with *n*BuLi) allowed nucleophilic addition into pentanal to afford protected hydroxylated olivetol **9**. Deprotection and condensation with citral using reported conditions^32^ afforded 1″-hydroxy-CBC (**10**) (Scheme 1).

### Direct Metabolism of CBC by Human Liver Microsomes Determined Using LC-MS/MS

**Figure 2:**
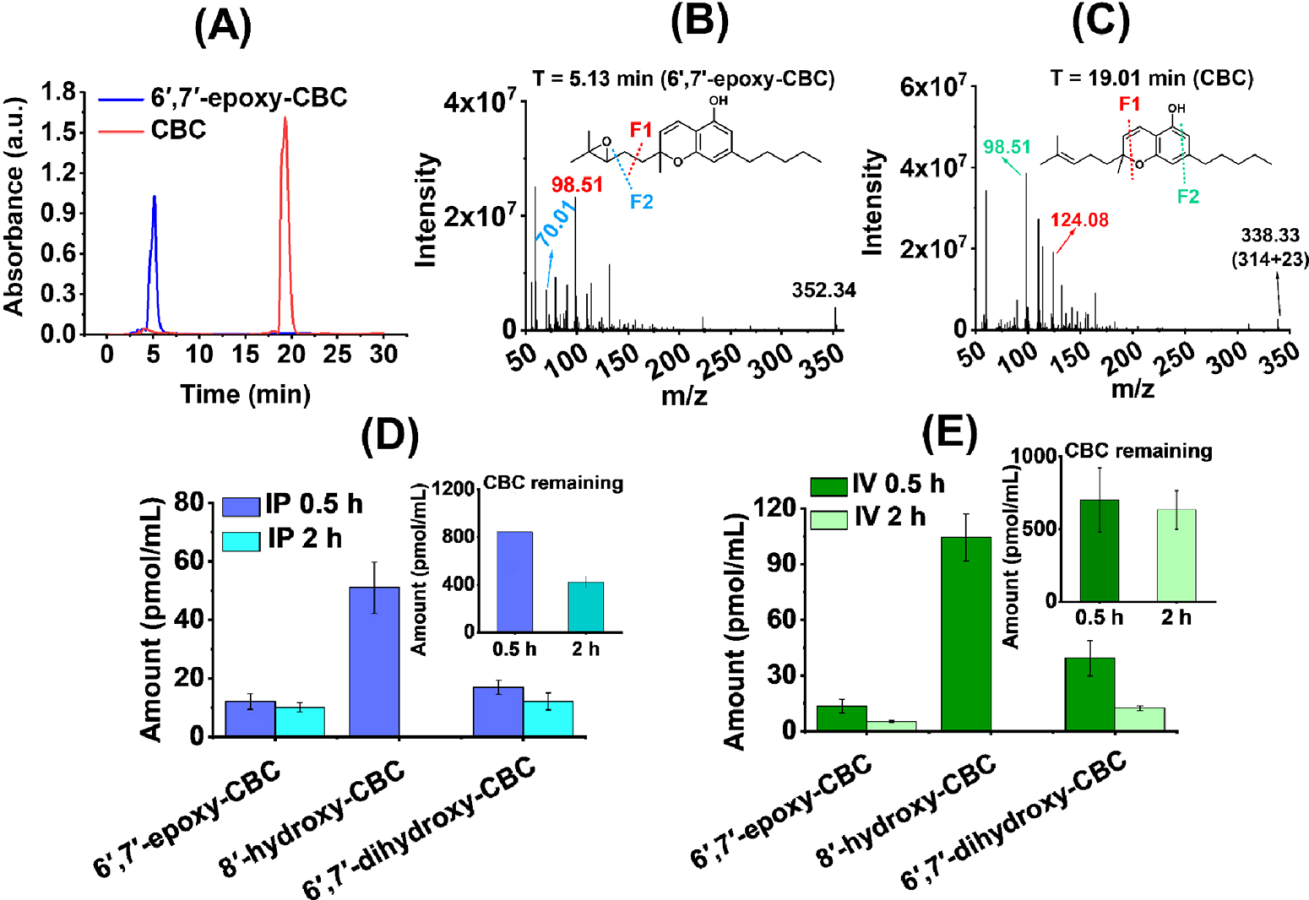
Detection of CBC and its metabolites: (A) LC-UV chromatogram of standard samples: CBC at 19.01 min (red line) and one of its metabolites 6′,7′-epoxy-CBC at 5.13 min (blue line). The corresponding mass fragmentation patterns are shown for (B) 6′,7′-epoxy-CBC and (C) CBC (all the other chromatogram and fragmentation patterns for different metabolites are shown in Supporting InformationI Figure S4). *In vivo* study: CBC and CBC metabolite obtained from the blood plasma of mouse after CBC administration through (D) intraperitoneal (IP) and (F) intravenous mode (IV). Samples were collected 0.5 and 2 h after administration and used for the analysis of CBC metabolites. The remaining CBC (not metabolized) at the corresponding time points is shown as insets for IP and IV. Data are represented as means ±SE of *n* = 3.

CBC has been demonstrated to undergo metabolism in the presence of rat or mice liver microsomes, resulting in the formation of diverse hydroxylated and epoxidized products.^35^ In this investigation, we employed human liver microsomes (HLMs) to conduct the metabolism of CBC. The products were then analyzed through liquid chromatography followed by mass spectrometry, and the results were compared with the mass fragments of the synthesized standard compounds (as shown in Figure 2A–C and Figures S2 and S3). The elution profile depicted in Figure S3 shows a sharp peak around 12 min (T5) along with a series of overlapping peaks between 3 and 4 min (T1–T4). Mass fragmentation for T1 (3.04 min) shows an intense peak around 347 *m*/*z* followed by characteristics peaks corresponding to 6′,7′-dihydroxy-CBC (**5**). The peak T2 at 3.19 min shows similar fragments to those of 6′,7′-epoxy-CBC (**4**), with a molecular ion peak at 352 *m*/*z*, indicative of the sodium adducts with 6′,7′-epoxy CBC. Only one peak is observed between 3.5 and 4 min, but upon studying the mass two different monohydroxylated compounds were identified; 1″-hydroxy-CBC (**10**) elutes around 3.5 min (T3) followed by 8′-hydroxy-CBC (**2**) at 3.96 min (T4). The molecular ion peak for 1″-hydroxy-CBC (**10**) and 8′-hydroxy-CBC (**2**) is detected at ∼352 *m*/*z*, again observed as the sodium adduct. The separate peak at ∼12 min was identified to be the substrate CBC with the molecular ion peak ∼338 (315 + 23/Na = 328). The overall LC-UV/MS chromatogram shows the formation of four distinct separate products.

### *In Vivo* Metabolism of CBC

CBC was administered via IV (intravenous) or IP (intraperitoneal) routes to mice. Plasma samples from mice were collected after 30 min and 2 h of administration. The plasma samples were extracted and analyzed using targeted mass spectrometry methods to estimate the abundance of various CBC metabolites (Figure 2D and E). CBC consumption was initially confirmed from the reduction in plasma CBC levels over the period of 2 h (shown in insets in Figure 2D and E as “CBC remaining”). The reduction in CBC levels was more prominent following the intraperitoneal mode of administration in comparison to the intravenous route. The products which have been detected from CBC metabolism are 6′,7′-epoxy-CBC (**4**), 8′-hydroxy-CBC (**2**), and 6′,7′-dihydroxy-CBC (**5**). The 8′-monohydroxy compound was determined to be the major metabolite, detectable at 4–8 times higher concentrations than the 6′,7′-epoxy compound after 0.5 h, depending on the mode of administration. Each of the three metabolites showed a significant reduction in their relative concentrations between 0.5 and 2 h. Interestingly, 8′-hydroxy-CBC (**2**) was not detected in the blood plasma after 2 h of CBC administration for both IP and IV.

### Bioactivity of CBC Metabolites

Microglia are resident macrophages present in the central nervous system. In response to injury, damage, or inflammation, microglial cells mount a response through various mechanisms such as the secretion of cytokines and chemokines. In this study, we used BV2 microglial cells, stimulated with lipopolysaccharide (LPS), to induce an inflammatory response in the presence and absence of CBC-based metabolites (Figure S5). This was followed by analyzing the expression levels of specific markers such as nitric oxide, IL-6, TNFα, and Arg-I. As depicted in Figure S5A, CBC and its metabolites reduced the production of nitric oxide as compared to cells treated with LPS only. Among the metabolites tested, a concentration-dependent reduction in the NO release can be observed for 7′,10′-ene-6′-hydroxy-CBC (**7**) and 6′,7′-dihydroxy-CBC (**5**). However, 8′-hydroxy-CBC (**2**) shows elevated levels of NO production. The levels of IL-6 are also reduced in the presence of CBC metabolites except for 8′-hydroxy-CBC (**2**) (Figure S5B). Concentration dependent reduction in IL-6 is observed for CBC, 7′,10′-ene-6′-hydroxy-CBC (**7**), 1″-hydroxy-CBC (**10**), and 6′,7′-dihydroxy-CBC (**5**). Interestingly, 6′,7′-dihydroxy-CBC (**5**) reduces the levels of both NO and IL-6 by almost 50% at 5 μM. Concentration dependent reduction in TNFα is observed for 7′,10′-ene-6′-hydroxy-CBC (**7**), 1″-hydroxy-CBC (**10**), and 6′,7′-dihydroxy-CBC (**5**). There is an increase in TNFα production with 8′-hydroxy-CBC **(2)** (Figure S6). The Arg-1 production increases in a concentration dependent manner for 6′,7′-epoxy-CBC (**5**) and 1″-hydroxy-CBC (**10**) (Figure S6). These observations are indicative that these cannabinoids (6′,7′-dihydroxy-CBC (**5**) and 7′,10′-ene-6′-hydroxy-CBC (**7**)) favor an anti-inflammatory phenotype by decreasing this pro-inflammatory marker while 8′-hydroxy-CBC is proinflammatory. Additionally, an MTT assay confirms that CBC and its metabolites are noncytotoxic to the cells (Figure S5C).

### Metabolism of CBC by Different Liver CYPs

**Figure 3:**
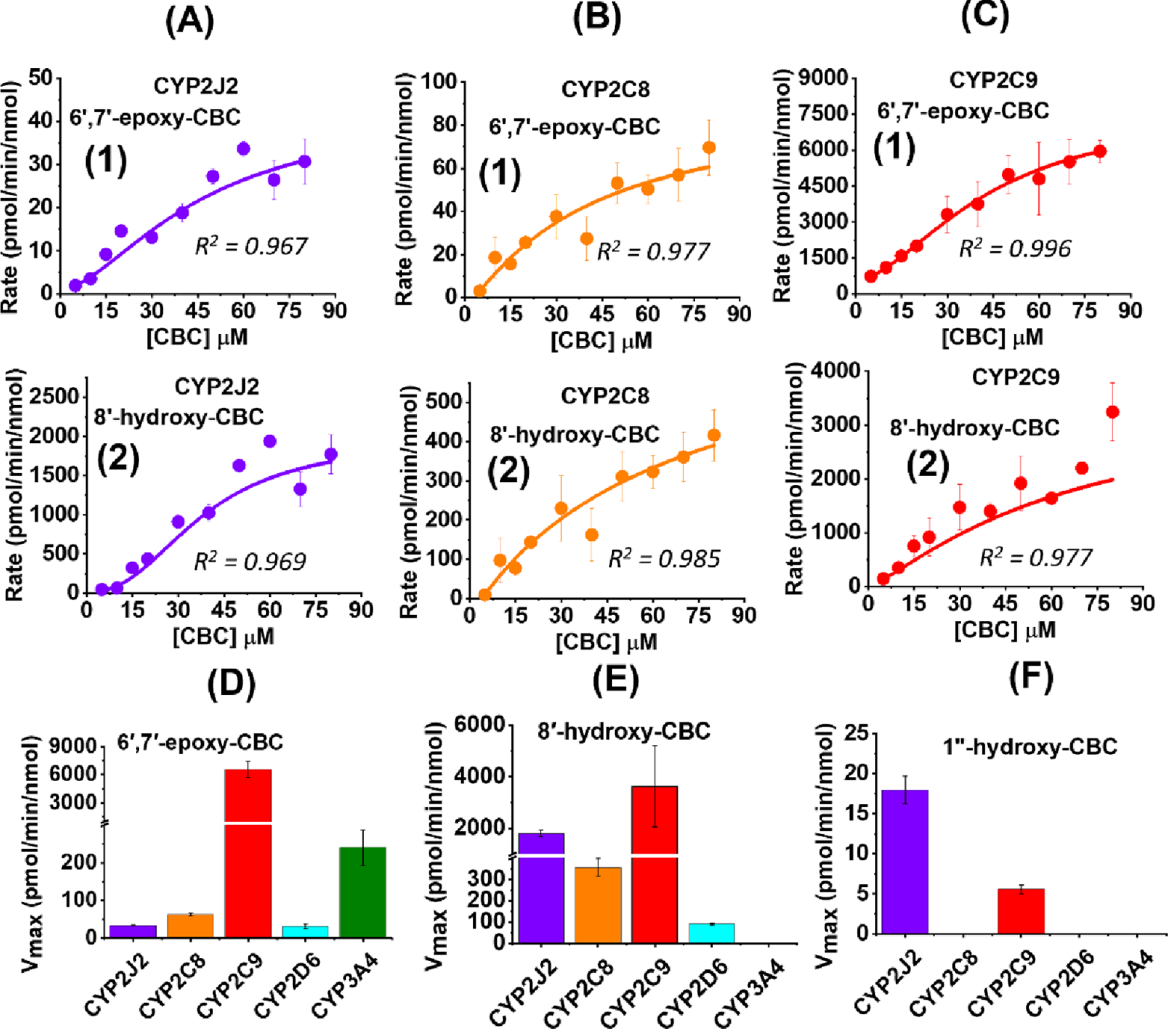
Metabolism of CBC by CYPs. Metabolism of CBC (0–80 μM) in the presence of CYPs leads to the formation of 6′,7′-epoxy-CBC (shown in the first row as 1) and 8′-hydroxy-CBC (shown in the second row as 2) for (A) CYP2J2, (B) CYP2C8, and (C) CYP2C9. The rate of product formation is analyzed in Origin software to get the *V*_max_ values. *V*_max_ for (D) 6′,7′-epoxy-CBC formation, (E) 8′-hydroxy-CBC formation, and (F) 1″-hydroxy-CBC formation are compared among different CYPs. Data has been fitted to either the Michaelis–Menten or Hill equation, error bars represent ± SEM, and *R*^*2*^ values for fittings are shown. (Plots for CYP2D6 and CYP3A4 are shown in the Supporting Information Figure S4 along with 1″-hydroxy-CBC formation.)

The kinetics of CBC metabolism were studied in the presence of the following recombinant CYPs using a lipid-constituted system: CYP2J2, CYP2C8, CYP2C9, CYP2D6, and CYP3A4. CBC upon metabolism by CYPs is converted to 6′,7′-epoxy-CBC (**4**) and 8′-hydroxy-CBC (**2**) (Figure 3A–D). Unlike the metabolites obtained from *in vivo* studies, 6′,7′-dihydroxy-CBC (**5**) is not detected in the presence of recombinant CYPs. However, 1″-hydroxy-CBC (**10**) is also formed by benzylic hydroxylation. The rates of product formation were fitted into the Hill equation, and the *V*_max_ was calculated from the plot which showed more than one binding site. Similar observations were reported for the metabolism of phytocannabinoids by CYPs where substrate binding to multiple sites often results in homotropic cooperativity.

Among all the metabolites produced by CYPs (Figure 3F), the relative concentration of 1″-hydroxy-CBC (**10**) stands out as significantly lower compared to the others. Interestingly 1″-hydroxy-CBC (**10**) is not detected in the presence of CYP2D6 and CYP3A4. CYP2J2 and CYP2C8 prefer 8′-hydroxy-CBC (**2**) formation over 6′,7′-epoxy-CBC (**4**). Our findings reveal that the ratio of 8′-hydroxy-CBC to 6′,7′-epoxy-CBC is approximately 50 for CYP2J2 and around 5 for CYP2C8 (Figure 3D and E). Moreover, the production of 8′-hydroxy-CBC (**2**) in the presence of CYP2J2 is nearly 10 times greater than that observed with CYP2C8. CYP2D6, on the other hand, displays a preference for 8′-hydroxy-CBC (**2**) formation by 3–4 times compared to 6′,7′-epoxy-CBC (**4**), but it has the lowest overall production of metabolites among all the CYPs. Notably, the rates of 6′,7′-epoxy-CBC (**4**) formation show only marginal differences for CYP2J2, CYP2C8, and CYP2D6, which is unlike the significant variance observed for 8′-hydroxy-CBC (**2**).

Among the CYPs, CYP2C9 shows the highest production of 6′,7′-epoxy-CBC (**4**) followed by CYP3A4 (Figure 3D). Interestingly, CYP3A4 exclusively produces 6 ′,7′-epoxy-CBC (**4**). While the extent of 8′-hydroxy-CBC (**2**) formation is approximately half that of 6′,7′-epoxy-CBC (**4**) for CYP2C9, it still demonstrates the highest rate among the CYPs, closely followed by CYP2J2 (Figure 3E). These findings highlight the diversity in product distribution based on the specific CYPs employed in the study.

### NADPH Oxidation by CYP–CPR in the Presence of Substrate

**Figure 4:**
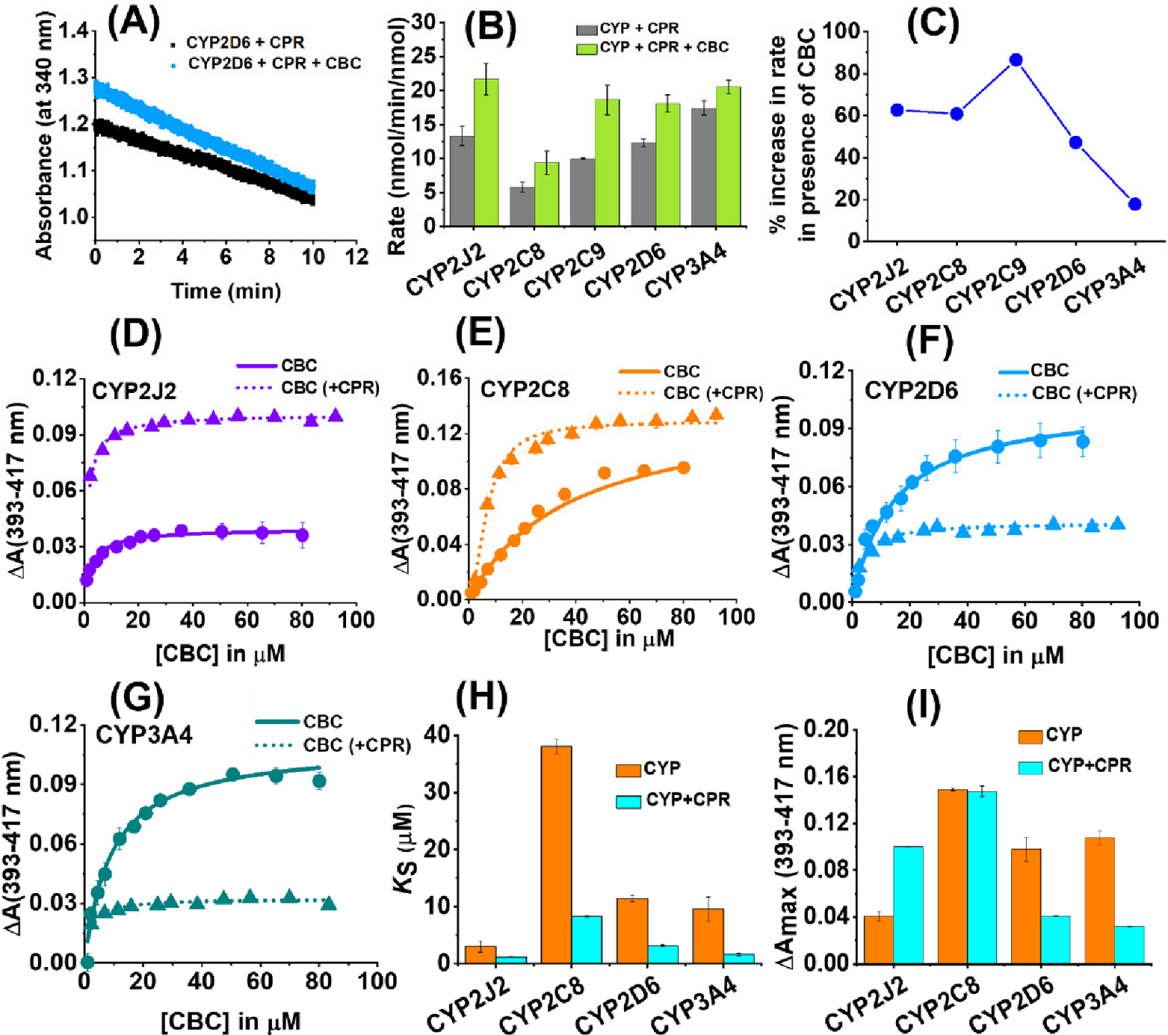
CBC binding and metabolism by CYPs. NADPH activity assay: Rate of change of absorbance of NADPH (at 340 nm) in the presence (deep blue) and absence (black) of CBC (50 μM) for (A) CYP2J2 with CPR as the redox partner. (B) Comparison of rate of NADPH consumption for different CYPs is shown in the absence of CBC (gray histogram) and presence of CBC (green histogram). (C) Percentage increase in the rate of NADPH oxidation when CBC is added to CYPs. Binding of CBC to CYP–CPR: Binding plots of CBC titration to CYPs are shown in the presence (dotted line) and absence (bold line) of CPR for (D) CYP2J2, (E) CYP2C8, (F) CYP2D6, and (G) CYP3A4. Comparison of (H) binding affinity *K*_s__(app)_ (in μM) and (I) change in spin shift due to heme perturbation Δ*A*_max_ among different CYPs is carried out.

Substrate metabolism by CYPs is primarily facilitated by the electron transfer from NADPH to cytochrome P450 reductase (CPR)^36^ to CYPs.^37^ During this process NADPH is oxidized to NADP^+^ after hydride transfer to the FAD binding domain of CPR.^38^ This process can be monitored by the decrease in absorbance at 340 nm.^39^ Herein, we have studied the rate of NADPH oxidation by CPR in the presence of different CYPs used in our study. We further compared the rate of NADPH consumption in the presence of 50 μM of CBC. Our results showed that the average rate of NADPH consumption for substrate-free CYP2J2, CYP2C9, and CYP2D6 is ∼10 nmol min^–1^ nmol^–1^ (Table S4, Figure S12). However, the rate for CYP2C8 (∼6 nmol min^–1^ nmol^–1^) is almost one-third that of substrate-free CYP3A4. The rates of NADPH oxidation showed a significant increase in the presence of CBC (Figure 4A). Apart from CYP2C8 (∼9 nmol min^–1^ nmol^–1^), the average rate of oxidation in the presence of CBC is ∼20 nmol min^–1^ nmol^–1^ for all the four CYPs (Figure 4B). Upon calculating the percentage increase of NADPH consumption in the presence of CBC, it can be found that CYP2C9 shows the maximum increase by ∼90% followed by CYP2J2, CYP2C8 (both ∼60%), and CYP3A4 (∼15%) (Figure 4C).

### Binding of CBC to Cytochrome P450s

CYPs generally show absorbance around 417 nm (Soret peak) which can shift to a lower wavelength or higher wavelength depending on the substrate. The lower shift is referred to as a Type I shift, resulting from distal water being replaced by the substrate. The higher shift is referred to as a Type II shift, resulting from the substrate directly coordinating with the heme iron. With this in mind, we investigated CBC’s binding activity with various CYPs, looking for variations in the absorption patterns specifically to help elucidate the mechanism of binding.

CYP2J2, CYP2C8, CYP2D6, and CYP3A4 upon titration with CBC showed a characteristic Type I shift. Cannabinoids generally act as Type I substrates for CYPs as highlighted in previous literature.^40,28^ The shift in the Soret spectra for different CYPs by CBC was used to calculate the change in the equilibrium constant (*K*_d(app)_) and the extent of spin state change due to heme perturbation (Δ*A*_max_). A standard one-site binding model was used to fit the binding curves for all the CYPs (*R*^*2*^∼0.9) from which the *K*_d(app)_ and Δ*A*_max_ values were evaluated (Figure 4D–G). Among the CYPs, CYP2J2 showed a tight binding curve with very high affinity of CBC toward the heme moiety (*K*_d(app)_ ∼ 3 μM) (Figure 4H). The binding affinities of CBC toward CYP2D6 and CYP3A4 are almost comparable (*K*_d(app)_ ∼ 11 and 9.5 μM, respectively); however, it has the least affinity toward CYP2C8 (*K*_d(app)_ ∼ 38 μM). On the contrary, CYP2C8 has the highest spin state change (Δ*A*_max_ ∼ 0.15) among all the CYPs in the presence of CBC whereas CYP2J2 has the least (Δ*A*_max_ ∼ 0.04). The heme perturbations for CYP2D6 and CYP3A4 are somewhat intermediate between the other two CYPs (Δ*A*_max_ ∼ 0.09 and 0.11, respectively).

### CBC Binding to CYP in the Presence of CPR

It is known that in the presence of substrate, CYP’s interaction with CPR changes. Therefore, we studied the binding of CBC to CYPs in the presence of CPR. Addition of CPR to the CYP causes a slight blue shift in the Soret peak; however, additional peaks are not observed for CYPs. Addition of CBC further causes a blue shift in the Soret peak indicating a Type I spin shift. The binding constant (*K*_d(app)_) values for CBC in the presence of CPR are observed below 10 μM for all the CYPs (Figure 4H), which is significantly lower compared to CBC binding to CYPs without CPR. The trend in binding affinity of CBC to CYPs is similar in the presence and absence of CPR, with CYP2C8 having the highest *K*_d(app)_ value and CYP2J2 having the lowest. The presence of CPR increases the binding affinity of CBC threefold for CYP2J2 (from ∼3 to ∼1 μM) and CYP2D6 (from ∼11 to ∼3 μM) and fivefold for CYP2C8 (from ∼38 to ∼8 μM) and CYP3A4 (∼9.5 to ∼1.6 μM). On the other hand, CBC can show heme perturbation in CYPs to a different extent in the presence and absence of CPR (Figure 4I). CYP2C8 (Δ*A*_max_ ∼ 0.15) shows the maximum spin change followed by CYP2J2 (Δ*A*_max_ ∼ 0.1), while CYP2D6 and CYP3A4 have the lowest spin change of ∼0.03–0.04. The overall study shows that CYP2J2 and CYP3A4 have higher affinity toward CBC in the presence of CPR and CYP2C8 shows the maximum spin state change.

### Computational Characterization of CBC Binding to CYP2J2

To study putative binding modes of CBC to CYP2J2, we used ensemble molecular dynamics (MD) and ensemble molecular docking, both of which have been implemented previously to characterize different ligand–CYP2J2 interactions.^41,42^ A CBC molecule was docked to the active site using Autodock Vina^43^, and the top 10 docked poses for each protein conformation were collected, resulting in a set of 2000 docked poses. Based on the docked poses and RMSD (root-mean-squared displacement), three different clusters were assigned. Clusters 1, 2, and 3 may represent different binding modes of CBC leading to the production of compound 6′,7′-epoxy-CBC, 1″-hydroxy- CBC, and 8′-hydoxy-CBC, respectively (Figure S9). To further analyze the stability of such representative binding modes using MD simulations, each binding mode was simulated for an additional 1 μs.

**Figure 5:**
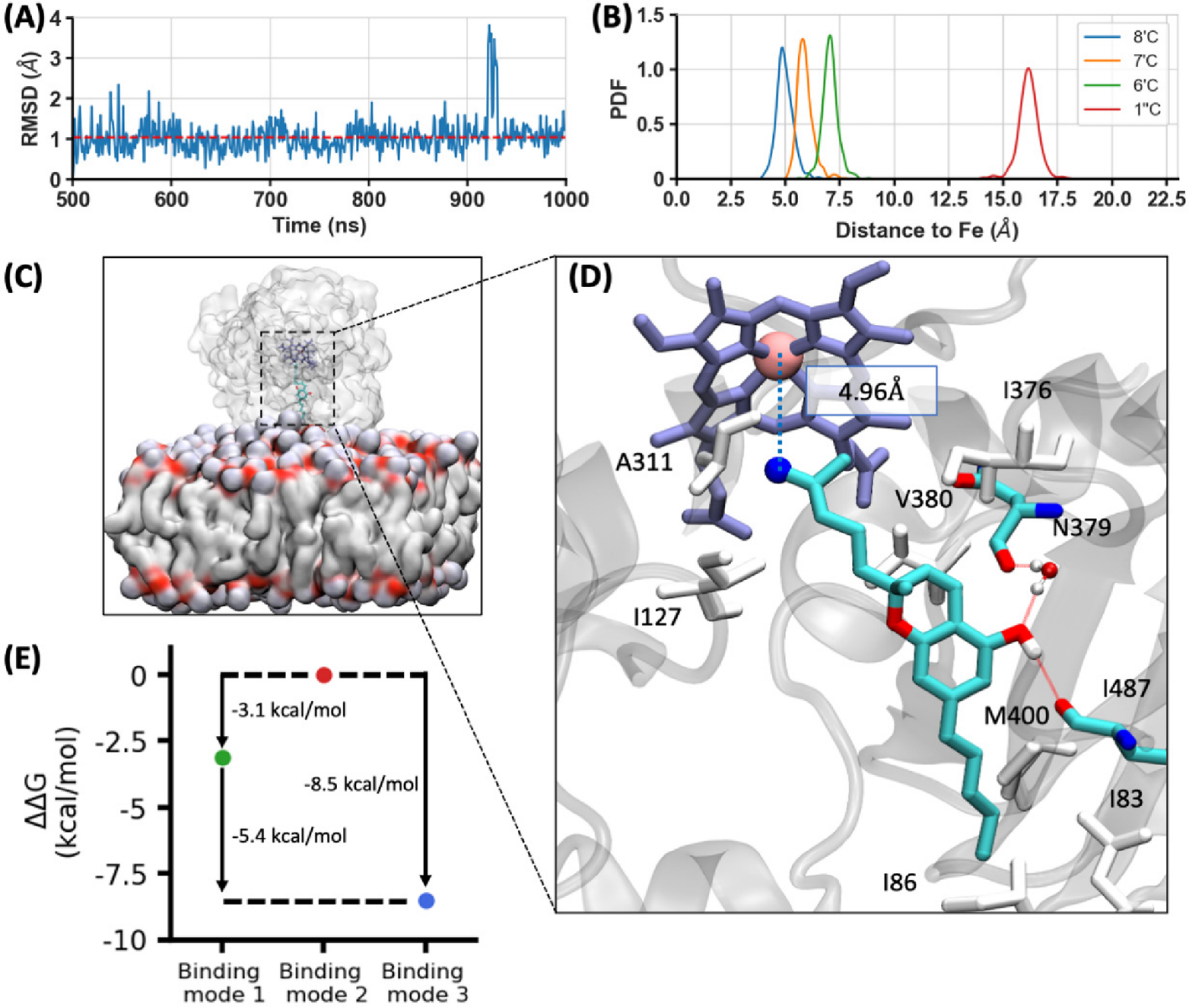
Putative binding mode of CBC to CYP2J2, which may lead to the production of 8′-hydroxy-CBC. (A) RMSD of CBC in binding mode 3 from the last 500 ns of the MD trajectory. Average RMSD is shown as a dashed red line. (B) The distribution of the selected carbon atom’s distance to the Fe atom in binding mode 3, resulting from the last 500 ns of the MD simulation. The heme distance distributions of 8′, 7′, 6′, and 1″-carbon atoms are shown as probability density functions (PDFs) in blue, orange, green, and red, respectively. (C) Representative snapshot of the MD setup with a docked CBC in the active site. CYP2J2 and the membrane are shown as surface representations. CBC and heme group are shown in cyan and lavender. (D) Representative snapshot of binding mode 3, highlighting some of the important residues surrounding CBC. Hydrophobic residues are colored in white. Atoms of N379 and I487 are shown in cyan (carbon), red (oxygen), and blue (nitrogen). A water molecule is shown in ball-and-stick representation. Hydrogen bonds are shown as red dashed lines. The distance between 8′-C (shown in blue) and Fe atom (shown in pink) is shown as a blue dashed line. (E) The relative binding free energies (ΔΔ*G*) of binding modes. The ΔΔ*G* of binding mode 1, 2, and 3 are shown in green, red and blue, respectively. The binding affinities of binding mode 1 and 3 are 3.1 and 8.5 kcal/mol stronger than that of binding mode 2, respectively.

After 500 ns, the RMSD of each binding mode is within 2 Å, indicating that they are stabilized in the active site cavity (Figure 5A, Figure S10A and B). Furthermore, binding mode 3 shows the 8′-carbon atom closest to the Fe atom among all binding modes with an average distance of 4.97 Å (Figure 5B, Figure S10C). This is in correlation with the experimental findings since 8′-hydoxy-CBC is formed as the major CBC metabolite in CYP2J2. Key amino acid residues interacting with CBC in binding mode 3 are highlighted in Figure 5D. Furthermore, our data suggests consistent hydrogen bonding, mainly between the CBC hydroxy group and the backbone oxygen of I487, as well as N379, mediated by a water molecule, potentially stabilizing this binding mode (Figure 5D). The 15 strongest interacting residues with each binding mode and representative snapshots of binding modes 1 and 2 are shown in Figures S10D and S11A and B.

In summary, we studied the metabolism of CBC by cytochrome P450s to form bioactive metabolites. We synthesized the authentic standards of the potential CBC metabolites that were predicted using molecular docking studies (epoxides and hydroxides), followed by the establishment of the targeted LC-MS/MS methods. When CBC was administered to mice (IV and IP mode), it was shown that in IV mode, the levels of metabolites spiked after 0.5 h while in IP mode the levels spiked at 2 h.^44^ There is lower concentration of 6′,7′-epoxy-CBC and 8′-hydoxy-CBC metabolites after 0.5 h. The formation of 6′,7′-dihydroxy-CBC (**5**) may be due to the presence of sEH enzyme (soluble Epoxide Hydrolase).^45^ This can be responsible for the lower amount of 6′,7′-epoxy-CBC (**4**) since it can get converted into the dihydroxy product (**5**) within the physiological system. The disappearance of 8′-hydroxy-CBC (**2**) from the system after 2 h despite being the major product is likely due to the subsequent oxidation of the terminal hydroxide group to carboxylic acid. Previously it has been shown that 11-OH-Δ9-THC is further oxidized to Δ9-THC-COOH *in vivo*, and this step is catalyzed by a microsomal aldehyde oxygenase (MALDO) which is a member of the CYP2C subfamily.^46,47^ It has been also found that liver microsomes obtained from mice can also form a significant amount of THC-COOH^18^ which points to the potential conversion of 8′-hydroxy-CBC (**2**) to its corresponding 8′-carboxy-CBC. Comparing the CBC metabolites from HLM and plasma samples from mice, 1″-hydroxy-CBC (**10**) is produced in the case of HLM and is not detected in the plasma samples from mice. This suggests that there is a difference in the CYP-mediated metabolism process between humans and mice which can be due to the variation in CYP genes.^48^

Metabolism of CBC by CYPs leads to the formation of 6′,7′-epoxy-CBC (**5**) and 8′-hydroxy-CBC (**2**) as major metabolites with traces of 1″-hydroxy-CBC. Kinetic plots indicate that CBC metabolism proceeds through a multisite binding process which has been shown to be true for other CYPs.^49^ For some CYPs such as CYP2J2, homotropic cooperativity operates.^50^ CYP2J2 is involved in metabolism of endocannabinoids and PUFA to their epoxy derivatives.^51^ The allylic position is favored by CYP2J2 over the saturated chain. Previously it was shown that oxidation of terfenadone by CYP2J2 resulted in allylic and benzylic hydroxylation as well as olefin epoxidation.^52^ Our experimental data shows that the amount of hydroxide formed is ∼50 more than the epoxide formation. From the docked structure (Figure S7A) it can be predicted that CBC adopts two different orientations at the active site pocket. The first orientation features the allylic group oriented toward the heme moiety (66% for CYP2J2), and the second features the alkyl chain oriented toward the heme moiety (34%). The energetically favorable docked structure for CYP2J2-CBC shows that the 8′-C is comparatively closer to the heme than 6′-C. This orientation of CBC at the active site of the protein is stabilized through the hydrogen bonding interaction with Arg117 (2.45 Å) present in the F-helix (Figure S8A). Arg117 acts as a key residue for substrate recognition in CYP2J2, and mutation of this residue increased the *K*_m_ as well as IC50 value with lowering of regioselectivity of the substrate.^52^ The orientation of the alkyl group away from the heme can be justified through the presence of hydrophobic residues like Val380 and Ile127 as well as the pi-stacking interaction between the aromatic ring in CBC and Phe310. This favors the formation of the 8′-hydroxy-CBC as the major metabolite for CYP2J2. To further probe into the dynamics of CBC at the active site, we have used molecular dynamics simulation within a solvent system. CBC shows major fluctuations within the active site pocket as seen from the RMSD values. However, the fluctuations are reduced significantly after 500 ns owing to the stabilization of the molecule at the active site. Our simulation results show that a putative binding mode of CBC which presents 8′-C to the heme group is stabilized in the active site via the nearby hydrophobic residues such as Ile127, Val380, and Phe310 (Figure 5D and Figure S10) and a hydrogen bonding network between the CBC hydroxy group and the backbone oxygen of I487 and N379, the latter mediated by a water molecule. Furthermore, this binding mode has the lowest relative binding free energy compared to the other two binding modes that position 6′-C or 1″-C toward the heme group (Figure 5E and Figure S11). Taken together, our results suggest that the most stable binding pose of CBC with the highest binding affinity favors the oxidation of the 8′-C in CBC over other regions.

Docking studies of CBC with all the remaining CYPs show that a combination of both electrostatic as well as hydrophobic interactions can favor a specific orientation of CBC in the active site (Figure S8). This in turn can regulate the formation of major metabolites for different CYPs. (Detailed discussion can be found in Supporting Information Section 17.)

The variation in the rate of CBC metabolism by different CYPs has been further supported by the NADPH oxidation assay. NADPH binds to CPR, followed by subsequent electron transfer from CPR to CYPs. In the absence of CYPs, the generation of oxidized CPR is prevented, which restricts the electron flow resulting in a lower NADPH oxidation rate (Figure S12E and F). When CBC is present, CYP2C9 exhibits the highest increase in the rate of NADPH oxidation, whereas CYP3A4 shows the least increase. This highlights the significance of CPR in conjunction with CYPs in regulating substrate binding and metabolism rate. It has been previously observed that substrates can influence the CYP–CPR interaction by binding at the protein–protein interface or at the allosteric sites.^53^ Additionally, substrates may partition between the membrane and protein surface, which affects their overall binding.^54^ Taking these conditions into account, it is possible that CBC can partition between CYPs and CPR by binding to both proteins to varying extents. This would suggest a lower affinity of CBC toward CYPs in the presence of CPR; however, the observed phenomenon is the opposite. A significant decrease in the *K*_d(app)_ for CBC toward CYPs in the presence of CPR is observed which implies an enhanced increase affinity of the substrate at the heme active site. Although the detailed mechanism remains unclear, theoretical studies have shown that the bottleneck radius of CYPs increases and remains open significantly for a longer time in the presence of CPR.^55^ This can facilitate easier access of CBC into the active site of CYP, thereby increasing the binding affinity of CBC.

The Δ*A*_max_ for CYP2C8 is found to be the highest, and the value does not differ significantly in the presence and absence of CPR. For CYP2J2 the Δ*A*_max_ is found to increase in the presence of CPR whereas it reduces for CYP2D6 and CYP3A4. Low values of Δ*A*_max_ obtained from our study for both CYP2D6 and CYP3A4 (in the presence of CPR) indicate that the distal water molecule attached to Fe is not significantly perturbed.^56^ The residue present in the I-helix has been also known to stabilize the distal water molecule through H-bonding^57^ which subsequently prevents the displacement of this water molecule by the dioxygen species. This can prevent the dioxygen activation^56^ in the heme thereby lowering the rate of substrate metabolism. Interestingly, in the presence of T309 V mutant, the enzyme activity increased up to 75-fold as compared to that of the wild-type.^57^ Herein, we have also tried to rationalize the significant difference in the rate of CBC metabolism by CYP2C9 and CYP3A4 using protein–protein docking studies. CYP–CPR structure is stabilized through hydrogen bonding interaction which plays a key role in the electron transfer process. Docking of the CYP–CPR complex with 6′,7′-epoxy-CBC and 8′-hydroxy-CBC showed that these metabolites can act as allosteric inhibitors for CYP3A4 by binding at the CYP–CPR interface (detailed discussion on protein–protein docking is given in the Supporting Information Section 18 and Figures S13 and S14).

## Experimental Section

### General Experimental Procedures

Human liver microsome (HLM) (1 mL at 20 mg/mL) was purchased from XenoTech which consists of mixed gender donors (pool of 50) and was dissolved in 250 mM of sucrose. The sample contains 0.579 nmol/mg protein of cytochrome P450 enzymes, 0.439 nmol/mg protein of cytochrome b5, and 171 ± 12 nmol/mg protein of NADPH-cytochrome c reductase. Lipopolysaccharides (*Escherichia coli*-O17:B8) were purchased from Sigma. The MTT assay kit (Cat No. 10009365), ELISA mouse IL-6 Kit (Cat No. 583371), Griess reagent-1 (Cat No. 780018), and Griess reagent-2 (Cat No. 780020) were obtained from Cayman Chemical Ltd. ELISA mouse IL-10 (Cat No. 88-7105) and TNFα Kits (Cat No. BMS607-3FIVE) were obtained from ThermoFisher Scientific. ELISA mouse Arginase 1 Kit (ab269541) was obtained from Abcam.

BV-2 cells were cultured and maintained at 37 °C in a humidified atmosphere of 95% air and 5% CO_2_ in high-glucose Dulbecco’s modified Eagle’s medium (Cat No. 10-013-CV, Corning Life Sciences), supplemented with 5% heat-inactivated fetal bovine serum, streptomycin (100 μg/mL), and penicillin (100 units/mL).

#### Expression and Purification of CYP P450 Enzymes

In this work five different CYP P450 enzymes were expressed (CYP 2J2, CYp2C8, CYP2C9, CYP2D6, and CYP3A4) and purified as per our previous report.^16^ Briefly, all the CYPs having His-tag were expressed in recombinant DH-5α *E. coli* along with pTGro7 plasmid using ampicillin (100 μg/mL) and chloramphenicol (20 μg/mL) as antibiotics. The protein supernatant which was separated from the membrane was loaded into Ni-NTA column and eluted in the presence of 0.1% cholate or cymal-5 using a high concentration of imidazole. The proteins were concentrated and buffer exchanged to remove the imidazole prior to using for any experiments.

#### Recombinant Expression of Cytochrome P450 Reductase (CPR)

Cytochrome P450 reductase serves as the redox partner for CYPs, and it was purified as previously described.^58^

#### CBC Metabolism in the Presence of Human Liver Microsome (HLM)

For HLM-mediated CBC metabolism, 9 μL of pooled HLM stock was dissolved in 0.1 M phosphate buffer (pH = 7.4) such that the final concentration of the CYP protein 0.4 μM (final volume calculated after addition of all the components = 500 μL). Two microliters of 10 mM CBC stock (dissolved in EtOH) was added (final CBC concentration 40 μM) and incubated with HLM for 10 min. The metabolism was triggered by adding 50 μL of 10 mM NADPH (final concentration 1 mM) and allowed to proceed at 37 °C for 30 min. The reaction was quenched by adding an equal volume of hexane:ethyl acetate mixture (Hex/EtOAc = 80:20 v/v). The quenched reactions were vortexed thoroughly and centrifuged for 5 min at 3300 rpm at 4 °C, and the organic layers were transferred into clean tubes. The extraction process was repeated for an additional two times to have a total of three extractions. The extracted metabolites (in Hex/EtOAc solvent) were dried using a rotavapor and resuspended in 100 μL of 95% ethanol. This sample was further used for LC/UV and LC/MS analysis.

#### LC-MS Analysis

CBC metabolites from HLM were analyzed using the Q-Exactive MS system (Thermo, Bremen, Germany) in the Metabolomics Laboratory of Roy J. Carver Biotechnology Center, University of Illinois at Urbana–Champaign. Software Xcalibur 4.1.31.9 was used for data acquisition and analysis. The Dionex Ultimate 3000 series HPLC system (Thermo, Germering, Germany) used includes a degasser, an autosampler, and a binary pump. Mass spectra were acquired under both positive and negative electrospray ionization (sheath gas flow rate, 37; aux gas flow rate: 8; sweep gas flow rate, 2; spray voltage, 3.5 kV; capillary temp, 250 °C; Aux gas heater temp, 400 °C). Compounds in the eluate were measured in full-scan mode at a resolution of 70 000 at *m*/*z* 200 and a scan range of *m*/*z* 50–750. The data-dependent MS2 was triggered after each full scan with a resolution of 17 500 at *m*/*z* 200 at a normalized collision energy of 40. The LC separation was performed on an Agilent Eclipse XDB-C_18_ (4.6 mm × 150 mm × 5 μm) 250 mm × 4.6 mm Luna 5 μm C_18_(2) 100 Å column. The 1200 series HPLC system (Agilent Technologies) includes a degasser, an autosampler, and a binary pump. The mobile phase A (5% acetonitrile in water) and mobile phase B (95% acetonitrile in water) were made to pass through the column with a flow rate of 0.15 mL/min with a linear gradient as follows: 0–5 min, 30% A and 70% B; 5–10 min, 5% A and 95% B; 10–15 min, 10% A and 90% B; 15–20 min, 15% A and 85% B; 20–15 min, 20% A and 80% B. The process was carried out at 4 °C with an injection volume of 10 μL. Positive mass spectra were acquired with the ion spray voltage of 5500 V under ESI.

#### CBC Metabolism by CYPs

To study the kinetics of CBC metabolism, concentration dependent metabolism of CBC was carried out in the presence of various CYPs using the following procedure. CYP2J2, CYP2C8, CYP2D6, CYP2C9, and CYP3A4 (final concentration 0.5 μM) were initially dissolved in lipid containing POPC:POPS (8:2 v/v) followed by addition of CPR (final concentration 0.6 μM) in 0.1 M potassium phosphate buffer (pH 7.4). CBC (5–80 μM) was added and incubated at 37 °C for 10 min. The reaction was triggered by NADPH and extracted using the exact sample protocol as mentioned in the HLM section.

#### CBC Administration to Female Mice

CBC was dissolved in ethanol, and an aliquot was then mixed with Tween-80. Ethanol was removed by evaporation. The mixture was then reconstituted with PBS to a 6% final concentration of Tween-80. C 57BL/6J female mice, age 8–10 weeks, were injected with 20 mg/kg CBC or vehicle intravenously (IV, *n* = 3) via the tail vein or intraperitoneally (IP, *n* = 3). Blood samples were collected terminally by cardiac puncture into heparin-coated syringes at 0.5 and 2 h after CBC treatment and transferred into tubes. The tubes were then centrifuged, and plasma samples were removed and stored at −80 °C.

#### Extraction of CBC and CBC Metabolites from the Blood Plasma

200 μL of blood plasma from each sample was taken into a glass tube, and 500 μL of extraction solvent (hexane:ethyl acetate 80:20 v/v) was added. The remaining extraction steps are similar to those used for CBC metabolite extraction from CYPs as mentioned earlier.

#### Quantitation of CBC Metabolite

Samples were analyzed with the 5500 QTRAP LC/MS/MS system (Sciex, Framingham, MA) in the Metabolomics Lab of Roy J. Carver Biotechnology Center, University of Illinois at Urbana–Champaign. Software Analyst 1.7.1 was used for data acquisition and analysis. The 1200 series HPLC system (Agilent Technologies, Santa Clara, CA) includes a degasser, an autosampler, and a binary pump. The LC separation was performed on a Phenomenex Gemini C_6_-phenyl column (2 × 100 mm, 3 μm) with mobile phase A (0.1% formic acid in water) and mobile phase B (0.1% formic acid in acetonitrile). The flow rate was 0.2 mL/min. The linear gradient was as follows: 0–2 min, 95% A; 10 min, 50% A; 20–25 min, 0% A; 25.1–31 min, 95% A. The autosampler was set at 10 °C. The injection volume was 5 μL. Mass spectra were acquired under positive electrospray ionization (ESI) with the ion spray voltage of +5000 V. The source temperature was 400 °C. The curtain gas, ion source gas 1, and ion source gas 2 were 325, 65, and 55 psi, respectively. Multiple reaction monitoring (MRM) was used for quantitation: CBC *m*/*z* 315.1 → *m*/*z* 135.0; 1-acetoxy-6′,7′-dihydroxy-CBC (JM-I-73) *m*/*z* 349.1→ *m*/*z* 193.0; 1″-hydroxy-CBC (JM-I-66) *m*/*z* 331.1→ *m*/*z* 275.1; 6′,7′-epoxy-CBC (JM-I-27) *m*/*z* 331.1→ *m*/*z* 193.0; 7′,10′-ene-6′-hydroxy-CBC (JM-I-30) *m*/*z* 331.1→ *m*/*z* 193.0; 8′-hydroxy-CBC (JM-I-32) *m*/*z* 334.1→ *m*/*z* 77.0. Internal standard EET-EA-d11 was monitored at *m*/*z* 375.2→ *m*/*z* 357.1.

#### NADPH Activity Assay

CYP-mediated NADPH oxidation was measured using kinetics mode in a Cary 300 UV–vis spectrometer (Agilent Technologies). 0.2 μM (final concentration) of CYP was taken in 0.1 M potassium phosphate buffer (pH 7.4) and incubated with 0.6 μM (final concentration) of CPR for 5 min with a reaction volume of 400 μL. The reaction was initiated with 200 μM of NADPH, and the absorption change was monitored over 10 min at 340 nm. The rate of NADPH consumption (or NADPH to NADP^+^ conversion) was calculated by taking the molar extinction coefficient as 6.22 mM^–1^ cm^–1^ at 340 nm. To estimate the change in NADPH oxidation in the presence of CBC, both CYP and CPR were further incubated using CBC (final concentration 50 μM) for 5 min followed by measuring the change in absorbance at 340 nm as discussed earlier.

#### Spectroscopic Titration of CBG with CYPs

CBC binding to various CYPs was determined by analyzing the Soret shift from ∼417 to ∼390 nm. CBC (stock 10 mM) was dissolved in ethanol and titrated with different CYPs at room temperature. Buffer-exchanged CYP2J2, CYP2D6, CYP3A4, and CYP2C8 (concentrations of proteins were kept between 4 and 5 μM) were dissolved in 0.1 M phosphate buffer (pH = 7.4), and initial spectra were recorded from 200 to 800 nm with a prominent peak at 417 nm. The concentration of CBC was varied from 0 to 100 μM keeping the final concentration of substrate in EtOH not more than 1% in cuvette. 0–4 μL of stock CBC was added using a Hamilton syringe in both the sample and blank cuvettes. The mixture was incubated for 5 min at 37 °C, and absorbance spectra were recorded using a Cary Bio 300 UV–vis spectrophotometer (Agilent Technologies, Santa Clara, CA). To eliminate the background absorbance, we derived the final spectra by subtracting the combined CYP–CBC spectra from that of CBC alone.

For CBC titration to CYP–CPR complex, initially CYPs were dissolved in 400 μL of 0.1 M phosphate buffer (pH = 7.4) keeping the concentration between 7 and 10 μL. Following this 50 μL of CPR (50 μM stock) was added to both cuvettes, and the final concentration of both of the proteins was 5–6 μM. Both proteins were initially mixed and incubated for 5 min followed by titration with CBC in the same process as mentioned above. The substrate-bound spectra were then subtracted from substrate-free spectra, and the extent of binding was analyzed using Origin Pro 2020.

### Biological Studies

Lipopolysaccharides (*E. coli*-O17:B8) were purchased from Sigma. The MTT assay kit (Cat No. 10009365), ELISA mouse IL-6 Kit (Cat No. 583371), Griess reagent-1 (Cat No. 780018), and Griess reagent-2 (Cat No. 780020) were obtained from Cayman Chemical Ltd. ELISA mouse IL-10 (Cat No. 88-7105) and TNFα Kits (Cat No. BMS607-3FIVE) were obtained from ThermoFisher Scientific. ELISA mouse Arginase 1 Kit (ab269541) was obtained from Abcam.

BV-2 cells were cultured and maintained at 37 °C in a humidified atmosphere of 95% air and 5% CO_2_ in high-glucose Dulbecco’s modified Eagle’s medium (Cat No. 10-013-CV, Corning Life Sciences), supplemented with 5% heat-inactivated fetal bovine serum, streptomycin (100 μg/mL), and penicillin (100 units/mL).

#### Microglial Cell Culture

BV2 cells were a gift from Dr. Bob McCusker, University of Illinois. They were cultured and maintained at 37 °C in a humidified atmosphere of 95% air and 5% CO_2_ in high-glucose Dulbecco’s modified Eagle’s (DMEM) medium (Cat No. 10-013-CV, Corning Life Sciences), supplemented with 5% heat-inactivated fetal bovine serum (FBS), streptomycin (100 μg/mL), and penicillin (100 units/mL). Stocks of all the minor cannabinoids were made in ethanol and stored at −80 °C and diluted as required before cell treatment. A final concentration of ethanol was maintained at 0.1% in the cell culture experiments.

First, cell viability of BV2 cells was assessed after treatment with cannabinoids, at various concentrations (0.1, 1.0, 2.5, 5.0 μM), utilizing Cayman’s MTT assay. It was concluded that these concentrations did not affect the cell viability for all cannabinoids. Once viability was determined, we measured the production of pro-inflammatory markers such as nitric oxide (NO), interleukin 6 (IL-6), tumor necrosis factor alpha (TNFα), and arginase 1 (Arg1) after BV2 cells were treated with minor cannabinoids and then stimulated with lipopolysaccharide (LPS) to evoke an inflammatory response.

#### Cell Viability Assay

The viability of BV-2 cells was evaluated by measuring the tetrazolium salt conversion using a 3-[4,5-dimethylthiazol-2-yl]-2,5-diphenyltetrazolium bromide (MTT) assay kit from Cayman Chemicals Ltd. Approximately 10 000 cells/well were seeded in a 96-well plate and grown for 24 h at 37 °C in a CO_2_ incubator.

Cells were preincubated for 4 h with compounds, followed by with or without stimulation with LPS (25 ng/mL) and an incubation period of 24 h. Following the 24 h incubation, the experiment was carried out according to the Cayman MTT protocol. Briefly, 10 μL of MTT reagent was added to the cells in the 96-well plate. After incubating for 4 h at 37 °C, the formazan crystals were formed, which were dissolved by adding 100 μL of crystal dissolving solution. Further incubation of cells for 24 h was allowed, and the absorbance was measured at 570 nm on a plate reader (SpectraMax iD5).

#### Nitric Oxide (NO) Determination

BV-2 cells were plated at a density of 50 000 cells/well in a 24-well plate at 37 °C under controlled conditions. Cells were pretreated with the minor cannabinoids at various concentrations (0.1, 1.0, 2.5, 5.0 μM) for 4 h and then stimulated with LPS for 24 h. The culture medium was removed and saved for the determination of nitric oxide production through Cayman’s Griess assay. IL-6, TNFα, and Arginase 1 production was also determined utilizing collected medium. For the Greiss assay, in a 96-well plate, 100 μL of culture medium was used, and 50 μL of both Greiss reagent-1 and Greiss reagent-2 was added and incubated for 10 min. The absorbance was measured at 540 nm using a plate reader (SpectraMax iD5). The quantification was done using a calibration curve that was generated using the sodium nitrite standard obtained from Cayman Chemicals.

#### ELISA Cytokine Determination

BV-2 cells were pretreated with the minor cannabinoids as described previously for 4 h in serum media (reduced to 5% FBS). Cells were stimulated with LPS (25 ng/mL in growth media) and allowed to incubate for 24 h. The culture medium was collected, and the concentrations of IL-6 (Cayman Chemicals), TNFα (ThermoFisher Scientific), and arginase 1 (ThermoFisher Scientific) cytokine expression were determined using specific monoclonal antibodies by ELISA kits as per manufacturer’s instructions.

#### Ensemble Molecular Docking and Molecular Dynamics Simulations

To characterize the putative binding modes of CBC to CYP2J2, ensemble molecular docking was performed using Autodock Vina.^43^ The structure of CYP2J2 was obtained from a previous publication in which CYP2J2 was modeled from CYP3A4.^59^ From the CYP2J2 equilibrium simulation, a snapshot was collected at every 100 ps from the last 20 ns of the trajectory, resulting in 200 unique protein conformations. Hence, an extensive conformational data set of CYP2J2 was created to be used in the ensemble docking protocol.^41,42^ To perform the ensemble docking, a 24 × 24 × 22 Å grid box was placed at center of the active site of CYP2J2, including the heme group. A single CBC molecule was docked into each of the 200 snapshots via Autodock Vina with an exhaustiveness of 40, with the top 10 poses collected for each protein snapshot. The resulting 2000 docked poses were clustered using a root-mean-square deviation (RSMD) metric,^60^ a cutoff distance of 3 Å between each cluster, and selecting the 8′, 7′, and 6′ carbon atoms as reference points. Selecting atoms from the end of the allylic chain of CBC (8′, 7′, and 6′-carbon atoms) resulted with the best separation between clusters in terms of the distance between the Fe atom and 6′, 1″, and 8′-carbon atoms. The resulting three clusters were further filtered using the binding score distribution of each cluster. A pose with the lowest binding score within the first quartile of the distribution was chosen as a representative binding mode for each cluster. The resulting three binding modes, in which the 6′, 1″, or 8′-carbon atom is closest to the Fe, represent the binding modes that may lead to the production of 6′,7′-epoxy-CBC, 1″-hydroxy-CBC, or 8′-hydroxy-CBC, respectively.

To determine the stability of the binding modes resulting from the docking, each representative pose was simulated using NAMD3,^61,62^ CHARMM36m force field^63^ parameters for the protein and lipids, and TIP3P^64^ for water, using a 2 fs time step. The parameters for CBC were obtained from the CHARMM General Force Field.^65^ Each system was first minimized for 2000 steps and equilibrated for 1 ns. Heavy atoms of CBC and the alpha carbons of protein were harmonically restrained (*k* = 1 kcal mol^–1^ Å^–2^). Equilibration was followed by a production run for an additional 1 microsecond. The temperature was maintained at 310 K using a Langevin thermostat, and the pressure was maintained at 1 bar using the Nosé–Hoover piston method.^66,67^ The particle mesh Ewald (PME) method^68^ was used to calculate long-range electrostatic interactions, with a maximum grid spacing of 1 Å. The nonbonded forces were calculated using a 12-Å cutoff and a 10-Å switching distance. Visual Molecular Dynamics (VMD)^69^ was used to visualize and analyze the simulations. Molecular Operating Environment (MOE) software (Molecular Operating Environment, 2019.01; Chemical Computing Group ULC, 1010 Sherbooke St. West, Suite #910, Montreal, QC, Canada, H3A 2R7, 2021) was used to plot 2D ligand–protein interaction diagrams.

To determine relative binding free energies of the three binding modes, free energy perturbation (FEP) simulations^70,71^ were performed using NAMD.^72^ To determine the representative snapshot of binding mode simulations, each simulation trajectory was clustered using an RSMD metric using a cutoff distance of 1.5 Å between each cluster and selecting CBC heavy atoms as reference points. A representative snapshot from the largest cluster of each binding mode simulation was chosen to perform FEP. During the FEP simulation, the ligand placed in bulk solution at least 25 Å away from the protein was annihilated while being created in the binding site. The forward (from λ = 0 to λ = 1; ligand disappearing from bulk solution and appearing in the active site) and backward (from λ = 1 to λ = 0; ligand disappearing from the active site and appearing in bulk solution) alchemical transformations were performed in 50 equally spaced consecutive windows (Δλ = 0.02) to evaluate the convergence and reversibility of FEP simulations and to achieve a gradual transformation. At each window, 100 ps of equilibration was followed by 1 ns of data collection. To prevent end-point catastrophes, a soft-core potential was applied with a 5 Å van der Waals shift coefficient. Cα atoms of the protein, CBC heavy atoms, and the heme group heavy atoms were harmonically restrained (*k* = 10 kcal mol^–1^ Å^–2^) to prevent conformational changes. The rest of the parameters were kept the same as the MD simulation protocol explained above. The results were analyzed using the ParseFEP plugin in VMD,^73^ and the Bennett acceptance ratio (BAR) method was used to estimate the statistical error.^74^

#### Docking Studies of CBC with Different CYPs

To analyze the binding region of CBC near the active site of the protein, protein–ligand docking studies were carried out between CBC and all the different CYPs. The crystal structures of the following CYPs were downloaded from RCSB: 1TQN (for CYP 3A4),^75^ 3TDA (for CYP 2D6),^76^ 5XXI (for 2C9),^77^ and 2NNI (for CYP 2C8).^78^ However, the structure of CYP2J2 was obtained through homology modeling as mentioned previously. The three-dimensional structure of CBG was prepared and optimized in Avogadro software.^79^ Prior to docking, the water molecules as well as other ligands associated with the protein structures were removed, and polar hydrogens as well as Kollman charges were added. The docking studies were carried out in Autodock Vina^43^ software with a grid size of 22 × 24 × 20 Å for CYP3A4, 24 × 20 × 20 Å for CYP2D6, 20 × 24 × 26 Å for CYP2C8, 26 × 24 × 22 Å for CYP2J2, and 20 × 12 × 14 Å for CYP2C9 with heme as the center. The coordinates of the centers are as follows: *x* = −19.128, *y* = −23.978, *z* = −13.910 for CYP3A4; *x* = 16.662, *y* = −24.053, *z* = 2.133 for CYP2D6; *x* = 47.198, *y* = 21.478, *z* = −28.956 for CYP2C8; *x* = 11.014, *y* = −7.454, *z* = 47.451 for CYP2J2; and *x* = 25.6312, *y* = 21.607, *z* = 29.324 for CYP2J9. All the docked structures were analyzed by using UCSF Chimera software.^80^

#### CPR Docking with CYPs

Protein–protein interaction between CPR and CYPs was carried out using the Haddock 2.4 web server.^81,82^ CPR exists as either an open or closed structure; however, the open structure interacts with CYPs. The crystal structure of the open conformation of CPR was downloaded from RCSB, PDB ID 3FJO,^83^ and the water molecules were removed from it keeping the prosthetic groups intact. Among the CYPs, CYP3A and CYP2C9 were selected for this study. Prior to the submission to the Haddock web server, the probable interacting residues were selected as active residues keeping all the other parameters as default. The active residues are as follows: ^118^YGEGDFPD^125^ for CPR, ^441^NCIGMR^446^ for CYP3A4,^84^ and K121, R125, R132, F134, M136, K138, K432, and G442 for CYP2C9.^85^ The result obtained after docking has multiple clusters which varies on their electrostatic or hydrophobic interaction. The cluster corresponding to the highest Haddock score (highest negative value) is further used for analysis.

#### Docking of the CYP–CPR Complex with CBC Metabolites

In order to understand the site of binding of the CBC metabolite in the CYP–CPR complex, 8′-hydroxy-CBC and 6′,7′-epoxy-CBC were docked with the complex. For the CYP3A4–CPR complex, only 6′,7′-epoxy-CBC was used for docking with a grid size of 32 × 28 × 28 Å and a center as *x* = 32.028, *y* = 2.500, *z* = 1.639. For the CYP2C9–CPR complex both 8′-hydroxy-CBC and 6′,7′-epoxy-CBC were separately used for docking with a grid size of 22 × 32 × 28 Å and a center as *x* = −47.179, *y* = 5.79, *z* = 10.145. The grid box covers both the heme as well as the FNM moieties of CYP and CPR, respectively. Docking was done using Autodock Vina, and the results were analyzed using UCSF Chimera.

## Supporting Information Available

Details of spectroscopic data, binding affinity values, ^1^H and ^13^C NMR spectra (PDF), docking studies, molecular dynamics, and detailed discussions (PDF)
