# Supplementary material for: Elucidating the Mechanism of Metabolism of Cannabichromene by Human Cytochrome P450s
Source: J Nat Prod. 2024 Mar 13;87(4):639–51. doi: 10.1021/acs.jnatprod.3c00336 (PMC11061835; doi:10.1021/acs.jnatprod.3c00336)
Supplement: Supplementary file 1 — np3c00336_si_001.pdf [file np3c00336_si_001.pdf]

Elucidating the Mechanism of Metabolism of Cannabichromene by Human Cytochrome P450s

Pritam Roy, Jonathan Maturano, Hale Hasdemir, Angel Lopez, Fengyun Xu, Judith Hellman,

Emad Tajkhorshid, David Sarlah* and Aditi Das*

AUTHOR INFORMATION

Corresponding Author

Aditi Das - School of Chemistry and Biochemistry, College of Sciences. Georgia Institute of Technology (GaTech), IBB, Parker H. Petit Institute for Bioengineering and Biosciences, Atlanta, GA 30332. Email: aditi.das@chemistry.gatech.edu

David Sarlah - Roger Adams Laboratory, Department of Chemistry, Cancer center at Illinois, University of Illinois, Urbana, Illinois 61801, United States Email: sarlah@illinois.edu

Authors

Pritam Roy - School of Chemistry and Biochemistry, College of Sciences. Georgia Institute of Technology, IBB, Parker H. Petit Institute for Bioengineering and Biosciences, Atlanta, GA 30332.

Jonathan Maturano - Roger Adams Laboratory, Department of Chemistry, Cancer center at Illinois, University of Illinois, Urbana, Illinois 61801, United States

Hale Hasdemir - Theoretical and Computational Biophysics Group, NIH Center for Macromolecular Modeling and Visualization, Beckman Institute for Advanced Science and Technology, Department of Biochemistry, and Center for Biophysics and Quantitative Biology, University of Illinois at Urbana−Champaign, Urbana, Illinois 61801, United States

Angel Lopez - School of Chemistry and Biochemistry, College of Sciences. Georgia Institute of Technology, IBB, Parker H. Petit Institute for Bioengineering and Biosciences, Atlanta, GA 30332.

Fengyun Xu - Judith Hellman Department of Anesthesia and Perioperative Care, University of California San Francisco San Francisco, CA.

Judith Hellman - Department of Anesthesia and Perioperative Care, University of California San Francisco San Francisco, CA.

Emad Tajkhorshid - Theoretical and Computational Biophysics Group, NIH Center for Macromolecular Modeling and Visualization, Beckman Institute for Advanced Science and Technology, Department of Biochemistry, and Center for Biophysics and Quantitative Biology, University of Illinois at Urbana−Champaign, Urbana, Illinois 61801, United States

Table of Contents

Contents

1 Synthesis of CBC based compounds 4
1.1 General Procedures 4
1.2 Experimental Section 4
1.3 ^{1}H NMR and ^{13}C NMR spectra 13
1.4 Mass Analysis 39
2 Table of comparison for Binding parameters 64
3 Possible fragmentation pattern of CBC and metabolites 65
4 LC-UV/MS of CBC and standard metabolites 66
5 LC/UV-MS of CBC metabolites in presence of Human Liver microsome 67
6 CBC Metabolism by different CYPs 68
7 Biological Study 69
7.1 NO assay, IL-6 assay and MTT Assay 69
7.2 Expression of TNFα and Arginase 1 70
8 Docking study 71
8.1 Different orientations of CBC in the active site of CYPs 71
8.2 Active sites around CBC in different docked poses 72
9 Distance of CBC from the heme Fe center in the active site of respective CYPs 73
10 Molecular Dynamics Simulation of CYP2J2 with CBC 74
10.1 Distance distributions of selected CBC atoms from the heme Fe center in CYP2J2 74
10.2 Average RMSD values and contact residues for CBC 75
10.3 Representative binding modes and free energy perturbation (FEP) calculations 76
11 NADPH activity assay 77
12 NADPH oxidation rates 78
13 CYP-CPR docked structure 79
14 Distance between CYP and CPR in the docked structure 80
15 Metabolites of CBC docked with CYP-CPR 81
16 H-bonding interaction of CBC metabolites and CYP-CPR 82
17 Docking studies of CBC at the active site of different CYPs 83
18 Discussion on protein-protein docking 84
19 MD simulation of CYP2J2 and CBC 85
20 References 88

1. Synthesis of CBC based compounds

1.1 General Procedures

All chemicals were purchased from commercial suppliers and used as received, unless otherwise noted. Acetonitrile (MeCN, HPLC grade), dichloromethane (CH_2Cl_2, HPLC grade), benzene (ACS grade), toluene (ACS grade), diethylether (ACS grade), tetrahydrofuran (ACS grade), acetone (HPLC grade), and methanol (ACS grade) were used as solvents without further purification. 4-dimethylaminopyridine (DMAP), triethylamine (Et_3N), acetic anhydride (Ac_2O), selenium dioxide (SeO_2), ethylenediamine, glacial acetic acid, N-methylmorpholine (NMO), osmium tetroxide (OsO_4), citric acid, mCPBA (*m*-chloroperoxybenzoic acid), TBSCl (tert-butyldimethylsilyl chloride), imidazole, *n*BuLi, TBAF (tetrabutylammonium fluoride), and pentanal, were purchased from commercial sources and used without further purification. Reaction temperatures correspond to the external temperature of the reaction vessel unless otherwise noted. Analytical thin-layer chromatography (TLC) was performed on Merck silica gel 60 F254 aluminum sheets. Visualization was accomplished with UV light and/or potassium permanganate ($KMnO_4$). Retention factor (R_f) values reported were measured using a 10 × 2 cm TLC plate in a developing chamber containing the solvent system described. Silicycle SiliaFlash® P60 (SiO2, 40–63 µm particle size, 230–400 mesh) was used for flash column chromatography. ^{1}H NMR spectra were obtained at 500 MHz and ^{13}C NMR were obtained at 126 MHz. NMR spectra were recorded using a Bruker Avance III 500 MHz spectrometer equipped with BB CryoProbe or Varian/Agilent VNMRS 750 MHz Narrow Bore and were referenced to residual chloroform (7.26 ppm, ^{1}H) and solvent chloroform-*d* (77.16, ^{13}C). Chemical shifts are reported in parts per million (ppm) and multiplicities are indicated as: s (singlet), d (doublet), t (triplet), q (quartet), p (pentet), m (multiplet), and br (broad). Coupling constants, *J*, are reported in Hertz. Mass spectrometry (MS) was performed by the University of Illinois Mass Spectrometry Laboratory. Electron Impact (EI+) spectra were performed at 70 eV using methane as the carrier gas, with time-of-flight (TOF) mass analyzer. Electrospray ionization (ES+) spectra were performed using a time-of-flight (TOF) mass analyzer. Data are reported in the form of m/z. Infrared (IR) spectra were measured neat on a Perkin-Elmer Spectrum Two FT-IR ATR spectrometer. Peaks are reported in cm^{-1} with indicated relative intensities: s (strong, 0 – 33% T); m (medium, 34 – 66% T), w (weak, 67 – 100% T), and br (broad).

1.2 Experimental Section

Synthesis of CBC *(2-methyl-2-(4-methylpent-3-en-1-yl)-7-pentyl-2H-chromen-5-ol)*

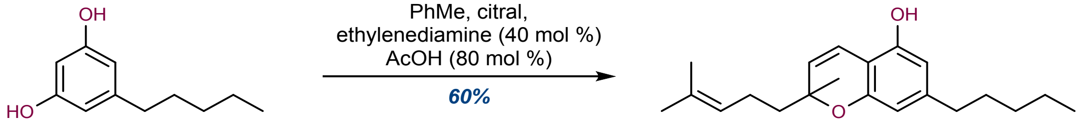

Reaction was carried out according to literature procedure[X]. To a solution of olivetol (6.18 g, 34.3 mmol, 1 equiv.) and citral (6.43 mL, 41.2 mmol, 1.2 equiv.) in toluene (343 mL, 0.1M), was added ethylenediamine (460 µL, 13.7 mmol. 0.4 equiv.) and AcOH (790 µL, 27.4 mmol, 0.8 equiv.) at room temperature. The reaction was heated and stirred at reflux for 6 hours before being cooled to room temperature and concentrated under reduced pressure. Crude product was purified via flash column chromatography

utilizing an eluent system of 30:1 to 10:1 hexane:EtOAc. Product was isolated as a yellow oil (6.50 g, 21 mmol, 60% yield).

R$_f$ – 0.20 (20:1 Hexane:EtOAc)

^{1}H NMR (500 MHz, $CDCl_3$) δ 6.61 (d, *J* = 10.0 Hz, 1H), 6.25 (s, 1H), 6.12 (s, 1H), 5.49 (d, *J* = 10.1 Hz, 1H), 5.10 (t, *J* = 7.2 Hz, 1H), 4.73 (s, 1H), 2.44 (t, *J* = 7.8 Hz, 2H), 2.14 – 2.07 (m, 2H), 1.75 – 1.68 (m, 1H), 1.66 (s, 3H), 1.64-1.61(m, 1H), 1.58 (s, 3H), 1.57-1.50 (m, 2H), 1.38 (s, 3H), 1.31 (td, *J* = 8.5, 7.3, 4.9 Hz, 4H), 0.88 (t, *J* = 6.8 Hz, 3H).

^{13}CNMR - ^{13}C NMR (126 MHz, $CDCl_3$) δ 154.19, 151.11, 144.90, 131.77, 127.38, 124.33, 116.91, 109.28, 107.80, 107.11, 78.31, 41.19, 36.04, 31.61, 30.77, 26.39, 25.81, 22.85, 22.68, 17.76, 14.16.

HRMS – (ES+, m/z) [M+H]+ calcd. for $C_{21}H_{31}O_2$ 315.2324; found, 315.2314

IR – (ATR, neat, cm1): 3404 (br), 2962 (s), 2928 (m), 2858 (s), 1624 (m), 1576 (m), 1430 (m), 1083 (m), 774 (m)

Synthesis of CBC-Ac (*2-methyl-2-(4-methylpent-3-en-1-yl)-7-pentyl-2H-chromen-5-yl acetate)*

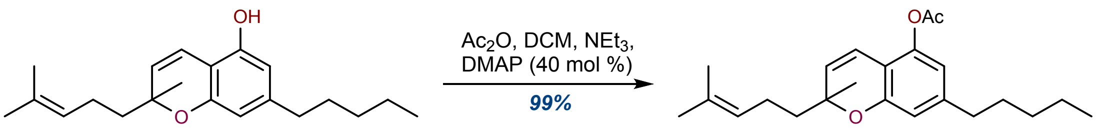

To a solution of CBC (1.03 g, 3.28 mmol, 1 equiv.) in DCM (32.8 mL, 0.1M) was added acetic anhydride (619 µL, 6.55 mmol, 2 equiv.) at room temperature. While stirring, DMAP (0.328 mmol, 0.1 equiv.) was added to the solution, followed by the dropwise addition of triethylamine at room temperature (1,37 mL, 9.83 mmol, 3 equiv.). Solution was monitored by TLC, and reaction proceeded to completion in 3 hours. The organic phase was washed with a 1M solution of HCl (30 mL) and the aqueous layer was extracted with DCM (2 30 mL) Extract as then dried with $MgSO_4$, filtered, and concentrated. The crude brown oil was purified by flash column chromatography using an eluent system of 15:1 to 10:1 to 7:1 hexane:EtOAc. Product was isolated as a brown oil (1.15 g, 3.23 mmol, 99%).

R$_f$ – 0.4 (15:1 hexane:EtOAc)

^{1}HNMR – ^{1}H NMR (500 MHz, $CDCl_3$) δ 6.51 (s, 1H), 6.41 (s, 1H), 6.31 (d, *J* = 10.0 Hz, 1H), 5.54 (d, *J* = 10.1 Hz, 1H), 5.09 (t, 1H), 2.50 (t, *J* = 7.8 Hz, 2H), 2.30 (s, 3H), 2.16 – 2.02 (m, 2H), 1.66 (s, 3H), 1.57 (s, 4H), 1.54 (s, 3H), 1.38 (d, *J* = 1.4 Hz, 3H), 1.35 – 1.27 (m, 4H), 0.88 (t, *J* = 6.7 Hz, 3H).

^{13}CNMR – ^{13}C NMR (126 MHz, $CDCl_3$) δ 169.34, 153.94, 146.31, 144.65, 131.87, 129.40, 124.18, 116.79, 114.11, 114.02, 111.77, 78.62, 41.27, 35.97, 31.60, 30.65, 26.48, 25.79, 22.83, 22.64, 20.98, 17.75, 14.13.

HRMS – (ES+, m/z) [M+H]+ calcd. for $C_{23}H_{33}O_3$, 357.2430; found, 357.2419

IR – (ATR, neat, cm1): 2961 (s), 2928 (s), 2858 (s), 1771 (m), 1624 (s), 1368 (s), 1200 (m), 1052 (m)

Synthesis of CBC-Ac-diol *(5-(5-hydroxy-2-methyl-7-pentyl-2H-chromen-2-yl)-2-methylpentane-2,3-diol)*

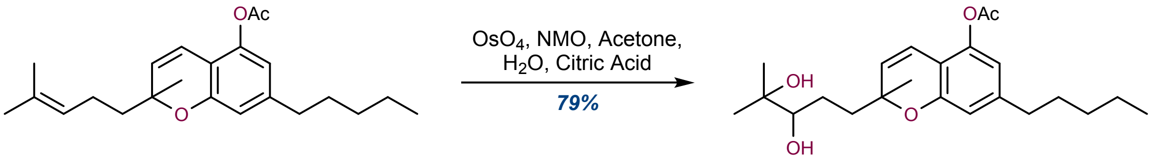

To a solution of CBC-Ac (180 mg, 0.572 mmol, 1 equiv.) in 10:1 Acetone:water (5.5:0.5 mL, 0.1 M), was added citric acid (220 mg, 1.14 mmol, 2 equiv.) and NMO (0.572 mmol, 1 equiv.). Next, OsO_4 in 0.2 M MeCN (29 µL, 5.72 µmol, 0.01 equiv.) was added at room temperature. The solution was left to stir for 24 hours, or until completion by TLC. The crude mixture was quenched with sodium thiosulfate and extracted with DCM (2 × 5mL). The crude product was concentrated under reduced pressure and purified via flash column chromatography (gradient eluent of 4:1 to 2:1 to 1:1 hexane:EtOAc). Product was isolated as a clear oil (158 mg, 0.453 mmol, 79%).

R_f – 0.2 (3:1 Hexane:EtOAc)

^{1}HNMR – ^{1}H NMR (500 MHz, $CDCl_3$) δ 6.51 (s, 1H), 6.42 (s, 1H), 6.33 (dd, J = 10.0, 4.3 Hz, 1H), 5.54 (dd, J = 10.0, 4.0 Hz, 1H), 3.34 (dt, J = 10.7, 2.4 Hz, 1H), 2.50 (t, 2H), 2.30 (s, 3H), 2.05 – 1.91 (m, 2H), 1.84 – 1.62 (m, 3H), 1.62 – 1.52 (m, 2H), 1.39 (s, 3H), 1.34 – 1.28 (m, 3H), 1.18 (d, J = 2.2 Hz, 3H), 1.12 (d, J = 12.5 Hz, 3H), 0.88 (t, J = 6.8 Hz, 3H).

^{13}CNMR - ^{13}C NMR (126 MHz, $CDCl_3$) δ 169.45,169.42, 153.85, 153.72, 146.36, 144.87, 144.81, 129.53, 129.07, 117.30, 117.16, 114.35, 114.30, 114.05, 113.98, 111.88, 111.81, 78.99, 78.86, 78.56, 73.34, 73.31, 38.78, 38.43, 35.96, 31.58, 30.67, 27.07, 26.72, 26.57, 26.41, 26.10, 23.43, 22.64, 21.00, 14.14.

HRMS – (ES+, m/z) [M+H]+ calcd. for $C_{23}H_{34}O_5$ 391.2484; found, 391.2474

IR – (ATR, neat, cm1): 3440 (br), 2956 (s), 2930 (m), 2858 (s), 1768 (m), 1624 (m), 1428 (m), 1368 (m), 1199 (m), 1053 (m)

<u>Synthesis of 5-(5-hydroxy-2-methyl-7-pentyl-2H-chromen-2-yl)-2-methylpentane-2,3-diol</u>

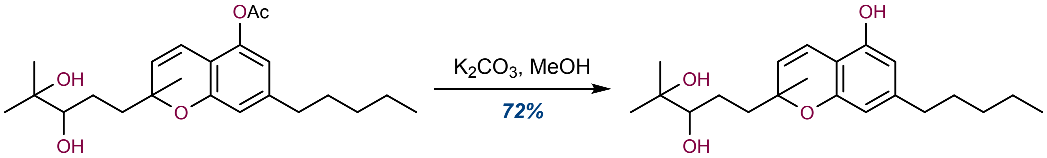

To a solution of CBC-Ac-diol (187 mg, 0.479 mmol, 1 equiv.) in MeOH (4.78 mL, 0.1M) was added potassium carbonate (165 mg, 1.2 mmol, 2.5 eq.) at room temperature. The reaction was left to stir for 2 hours until the consumption of starting material was observed by TLC and the solution was a deep purple. The solution was concentrated, washed with 1M HCl (5 mL) and extracted with DCM (3 × 5mL). Extract was then dried with $MgSO_4$, filtered, and concentrated. Product was purified by flash column chromatography using an eluent system of 20:1 DCM:MeOH. Product was isolated as a clear oil (121 mg, 0.347 mmol, 72%).

^{1}HNMR – ^{1}H NMR (500 MHz, $CDCl_3$) δ 6.63 (d, J = 10.0 Hz, 1H), 6.24 (s, 1H), 6.13 (s, 1H), 5.49 (d, J = 10.0 Hz, 1H), 4.82 – 4.65 (m, 1H), 3.37 (t, 1H), 2.44 (t, J = 7.8 Hz, 2H), 2.06 – 1.91 (m, 1H), 1.85 – 1.64 (m, 3H), 1.60 – 1.54 (m, 2H), 1.38 (s, 2H), 1.35 – 1.24 (m, 4H), 1.19 (d, J = 8.4 Hz, 3H), 1.14 (d, J = 5.7 Hz, 3H), 0.89 (t, 3H).

^{13}CNMR – 13C NMR (126 MHz, CDCl3) δ 153.90, 153.82, 151.21, 145.09, 145.05, 127.52, 127.04, 117.34, 117.31, 109.28, 109.22, 108.09, 108.02, 107.14, 107.00, 79.00, 78.67, 78.64, 78.28, 73.38, 73.34, 38.49, 38.22, 36.03, 31.62, 30.78, 26.74, 26.71, 26.66, 26.62, 26.13, 26.11, 23.46, 23.36, 22.67, 14.17.

HRMS – (ES+, m/z) [M+H]+ calcd. for $C_{21}H_{33}O_5$, 349.2379; found, 349.2376

IR – (ATR, neat, cm1): 3367 (br), 2960 (m), 2929 (m), 2858 (m), 1623 (m), 1577 (m), 1430 (m), 1060 (m)

Synthesis of CBC-Ac Epoxide *(2-(2-(3,3-dimethyloxiran-2-yl)ethyl)-2-methyl-7-pentyl-2H-chromen-5-yl acetate)*

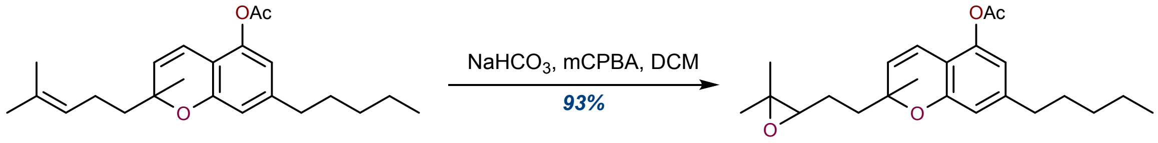

To a solution of CBC-Ac (131 mg, 0.369 mmol, 1 equiv.) in DCM (4.0 mL, 0.1 M) was added sodium bicarbonate (39 mg, 0.461 mmol, 1.25 equiv.). mCPBA (85 mg, 0.369 mmol, 1 equiv.) was added to the solution in a single addition and left to stir at room temperature overnight. Upon completion by TLC, reaction was quenched with a solution of saturated aqueous sodium bicarbonate and sodium thiosulfate (4 mL), then extracted with DCM (3 × 4 mL). Extract was then dried with $MgSO_4$, filtered, and concentrated. The crude oil was purified by flash column chromatography using an eluent system of 20:1 to 15:1 to 10:1 to 5:1 Hexane:EtOAc. Product was isolated as a greenish oil (128 mg, 0.345 mmol, 93%).

R_f – 0.6 (3:1 hexane:EtOAc))

^{1}HNMR – 1H NMR (500 MHz, CDCl3) δ 6.50 (s, 1H), 6.45 – 6.40 (m, 1H), 6.33 (d, J = 10.1 Hz, 1H), 5.52 (dd, J = 14.6, 10.1 Hz, 1H), 2.76 – 2.67 (m, 1H), 2.50 (t, J = 7.8 Hz, 2H), 2.30 (d, J = 1.7 Hz, 3H), 1.93 – 1.53 (m, 6H), 1.39 (s, 3H), 1.36 – 1.29 (m, 4H), 1.28 (d, J = 7.9 Hz, 3H), 1.22 (d, J = 3.1 Hz, 3H), 0.88 (t, J = 6.7 Hz, 3H).

^{13}CNMR - 13C NMR (126 MHz, CDCl3) δ 169.34, 153.76, 153.69, 146.39, 144.85, 129.14, 128.73, 117.35, 117.16, 114.31, 114.28, 114.05, 113.97, 111.69, 111.55, 78.54, 78.13, 64.57, 64.30, 58.75, 58.59, 38.14, 37.71, 35.97, 31.60, 30.65 (d, J = 2.9 Hz), 26.90, 26.31, 25.00, 24.11, 23.74, 22.64, 21.00, 18.81, 18.76, 14.14.

HRMS – (ES+, m/z) $[M-H]^-$ calcd. for $C_{23}H_{33}O_4$, 373.2371; 373.2379

IR – (ATR, neat, cm1): 2960 (m), 2928 (m), 2858 (s), 1770 (m), 1624 (m), 1369 (m), 1201 (m), 1054 (m)

Synthesis of CBC-Epox *2-(2-(3,3-dimethyloxiran-2-yl)ethyl)-2-methyl-7-pentyl-2H-chromen-5-ol*

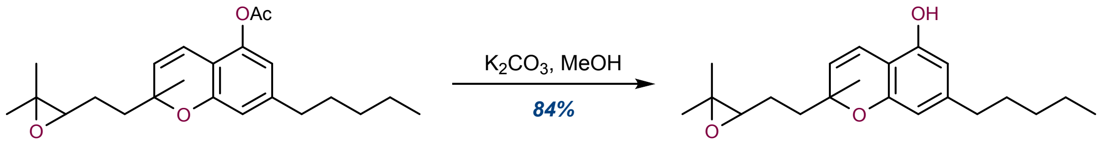

To a solution of CBC-Ac-Epox (128 mg, 0.343 mmol, 1 equiv.) in MeOH (3.4 mL, 0.1 M), was added K_2CO_3 (119 mg, 0.858 mmol, 2.5 equiv.) at room temperature. The solution was left to stir and monitored by TLC until completion (about 1 hour). The solution was concentrated, washed with 1M HCl (4 mL) and

extracted with EtOAc (3 × 4 mL). Extract was then dried with $MgSO_4$, filtered, and concentrated. Crude appeared as an oil which was purified by flash column chromatography (6:1 to 5:1 to 4:1 hex:EtOAc) to afford the product as a greenish oil (96 mg, 0.290 mmol, 84%).

R_f – 0.6 (3:1 Hexane: EtOAc)

^{1}HNMR – ^{1}H NMR (500 MHz, $CDCl_3$) δ 6.66 (d, *J* = 10.0 Hz, 1H), 6.20 (s, 1H), 6.14 (s, 1H), 6.06 (s, 1H), 5.43 (dd, *J* = 19.5, 10.0 Hz, 1H), 2.80 (dt, *J* = 15.2, 6.1 Hz, 1H), 2.42 (t, *J* = 7.8 Hz, 2H), 1.85 – 1.63 (m, 4H), 1.54 (dt, *J* = 12.6, 6.2 Hz, 2H), 1.37 (d, *J* = 4.7 Hz, 3H), 1.30 (d, *J* = 6.3 Hz, 7H), 1.25 (d, *J* = 2.7 Hz, 3H), 0.87 (t, *J* = 6.8 Hz, 3H).

^{13}CNMR – ^{13}C NMR (126 MHz, $CDCl_3$) δ 153.86-153.79, 144.91, 126.62-126.21, 117.82-117.64, 108.79-108.70, 108.09-108.04, 107.05-106.88, 78.09-77.67, 65.24-64.90, 59.69-59.44, 38.00-37.44, 36.03, 31.58, 30.75-30.73, 26.76, 26.18, 24.93, 24.00, 23.62, 22.64, 18.71 (d, *J* = 8.7 Hz), 14.13.

HRMS – (ES+, m/z) $[M-H]^-$ calcd. for $C_{21}H_{31}O_3$, 331.2273; found, 331.2260

IR – (ATR, neat, cm1): 3340 (br), 2959 (m), 2927 (m), 2857 (s), 1622 (m), 1577 (m), 1429 (m), 1138 (m), 774 (m)

Synthesis of CBD-TBS *(tert-butyldimethyl((2-methyl-2-(4-methylpent-3-en-1-yl)-7-pentyl-2H-chromen-5-yl)oxy)silane)*

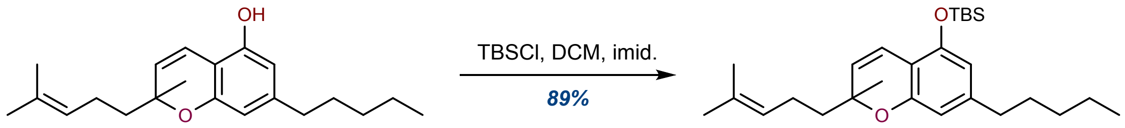

To a solution of CBC (1.02 g, 3.24 mmol, 1 equiv.) in DCM (32.4 mL, 0.1M) was added TBSCl (587 mg, 3.89 mmol, 1.2 equiv.) while vigorously stirring at room temperature. Next, imidazole (486 mg, 7.14 mmol, 2.2 equiv.) was added to the solution in a single portion. Solution was left to stir and monitored to completion by TLC. Solution was quenched with 1M HCl (30 mL) and the aqueous layer was extracted with DCM (3 × 30 mL). Extract was then dried with $MgSO_4$, filtered, and concentrated. Crude appeared as an oil which was purified by flash column chromatography using an eluent system of 20:1 hexane:EtOAc. Product was isolated as a clear oil (1.24 g, 2.89 mmol, 89%).

R_f – 0.1 (hexane)

^{1}HNMR – ^{1}H NMR (500 MHz, $CDCl_3$) δ 6.60 (d, *J* = 10.1 Hz, 1H), 6.27 (s, 1H), 6.15 (s, 1H), 5.45 (d, *J* = 10.0 Hz, 1H), 5.09 (t, *J* = 7.5 Hz, 1H), 2.44 (t, *J* = 7.6 Hz, 2H), 2.14 – 2.05 (m, 2H), 1.66 (s, 3H), 1.60 - 1.53 (m, 7H), 1.36 (s, 3H), 1.35 – 1.24 (m, 4H), 1.00 (s, 9H), 0.88 (t, *J* = 6.8 Hz, 3H), 0.20 (s, 6H).

^{13}CNMR – ^{13}C NMR (126 MHz, $CDCl_3$) δ 154.14, 151.28, 144.34, 131.70, 127.08, 124.42, 118.25, 111.86, 111.02, 109.75, 78.07, 41.24, 36.13, 31.56, 30.85, 26.43, 25.97, 25.82, 22.87, 22.68, 18.46, 17.74, 14.17, -4.11.

HRMS – (ES+, m/z) [M+H]+ calcd. for $C_{27}H_{45}O_2Si$, 429.3189; found, 429.3171

IR – (ATR, neat, cm1): 2957 (m), 2928 (m), 2858 (m), 1612 (m), 1563 (m), 1430 (m), 1252 (m), 1103 (m), 1084 (m), 1068 (m), 835 (m), 779 (m)

Synthesis of *(tert-butyl((2-(2-(3,3-dimethyloxiran-2-yl)ethyl)-2-methyl-7-pentyl-2H-chromen-5-yl)oxy)dimethylsilane)*

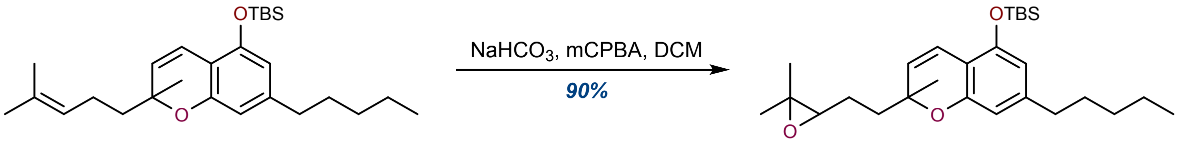

To a solution of CBC-TBS (307 mg, 0.716 mmol, 1equiv.) in DCM (9 mL, 0.1M) was added sodium bicarbonate (75 mg, 0.895 mmol, 1.25 equiv.) at room temperature. mCPBA (165 mg, 0.716 mmol, 1 equiv.) was added to the solution and left to stir at room temperature overnight. Upon completion by TLC, reaction was quenched with a solution of saturated aqueous sodium bicarbonate and sodium thiosulfate (9 mL), then extracted with DCM (3 × 9 mL). Extract was then dried with $MgSO_4$, filtered, and concentrated. The crude oil which was purified by flash column chromatography using an eluent system of 20:1 to 15:1 to 10:1 to 5:1 Hexane:EtOAc. Product was isolated as a greenish oil (285 mg, 0.641 mmol, 90%).

R_f – 0.4 (Hexane:EtOAc 15:1)

^{1}H NMR (400 MHz, $CDCl_3$) δ 6.62 (d, J = 9.9 Hz, 1H), 6.26 (s, 1H), 6.16 (s, 1H), 5.44 (d, J = 9.9 Hz, 0.5H), 5.41 (d, J = 10.2 Hz, 0.5H), 2.77 – 2.67 (m, 1H), 2.44 (t, J = 7.6 Hz, 2H), 1.82 (t, J = 7.8 Hz, 1H), 1.77 – 1.62 (m, 3H), 1.60 – 1.50 (m, 2H), 1.38 (s, 3H), 1.35 – 1.29 (m, 3H), 1.28 (d, J = 5.8 Hz, 4H), 1.22 (s, 3H), 1.01 (s, 9H), 0.88 (t, J = 6.7 Hz, 3H), 0.21 (s, 6H).

^{13}CNMR – ^{13}C NMR (126 MHz, $CDCl_3$) δ 153.97, 153.88, 151.36, 144.53, 126.82, 126.38, 118.73, 118.56, 112.00, 111.94, 110.89, 110.71, 109.75, 109.64, 77.99, 77.58, 64.71, 64.43, 58.72, 58.54, 38.11, 37.58, 36.12, 31.55, 30.84, 30.82, 26.86, 26.27, 25.96, 25.02, 24.09, 23.76, 22.67, 18.82, 18.75, 18.46, 14.17, -4.10, -4.12.

HRMS – (ES+, m/z) [M+H]+ calcd. for $C_{27}H_{44}O_3Si$, 445.3138; found, 445.3126

IR – (ATR, neat, cm1): 2957 (s), 2929 (s), 2858 (s), 1612 (s), 1563 (s), 1426 (s), 1253 (s), 1117 (s), 1103 (s0), 1078 (s), 837 (m), 779 (m)

Synthesis of *5-(5-((tert-butyldimethylsilyl)oxy)-2-methyl-7-pentyl-2H-chromen-2-yl)-2-methylpent-1-en-3-ol*

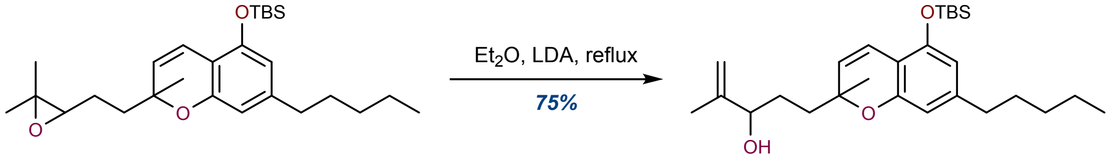

To a solution of LDA (1.0 mL, 1.6 mmol, 2.5 equiv.) in Et_2O (3.2 mL, 0.2 M) under inert atmosphere, was added a solution of CBC-TBS-epox (285 mg, 0.642 mmol, 1 equiv.) in Et_2O (3.2 mL, 0.2M) dropwise over a few minutes at room temperature. The solution was then refluxed and monitored until complete by TLC. Solution was quenched with 1M HCl (6 mL) and extracted with Et_2O (3 × 7 mL). Extract was then dried with $MgSO_4$, filtered, and concentrated. The crude oil was purified by flash column chromatography using an eluent system of 20:1 then 10:1 Hexane:EtOAc. Product was isolated as a greenish oil (213 mg, 0.479 mmol, 75%).

R_f – 0.6 (3:1 hexane:EtOAc)

^{1}HNMR – ^{1}H NMR (500 MHz, $CDCl_3$) δ 6.61 (d, J = 10.0, 1H), 6.26 (s, 1H), 6.15 (s, 1H), 5.67 – 5.61 (m, 0.5H), 5.42 (d, J = 10.1 Hz, 1H), 5.30 (s, 0.5H), 4.92 (s, 1H), 4.82 (s, 1H), 4.08 – 4.02 (m, 1H), 2.44 (t, J = 7.7 Hz, 2H), 1.78

– 1.71 (m, 1H), 1.69 (s, 3H), 1.60 – 1.50 (m, 3H), 1.39 (s, 1H), 1.36 (s, 2H), 1.34 – 1.27 (m, 5H), 1.00 (s, 9H), 0.87 (t, 3H), 0.20 (s, 6H).

^{13}CNMR - ^{13}C NMR (126 MHz, $CDCl_3$) δ 154.29, 153.92, 151.32, 151.26, 147.53, 147.40, 144.50, 144.45, 141.69, 134.11, 126.99, 126.79, 126.51, 123.39, 121.57, 118.54, 118.47, 118.45, 111.97, 111.85, 111.37, 111.19, 111.10, 110.91, 110.83, 109.70, 109.68, 109.60, 78.10, 77.89, 77.79, 76.12, 75.92, 70.78, 44.07, 37.17, 36.98, 36.11, 31.55, 30.83, 29.69, 29.66, 29.62, 29.34, 26.67, 26.60, 26.39, 25.96, 22.67, 18.45, 17.86, 17.67, 14.17, -4.12.

HRMS – (ES+, m/z) [M+H]+ calcd. for $C_{27}H_{45}O_3Si$, 445.3138; found, 445.3120

IR – (ATR, neat, cm1): 3403 (br), 2956 (s), 2929 (m), 2858 (s), 1612 (s), 1563 (s), 1105 (m), 1074 (m), 835 (m), 778 (m)

Synthesis of *2-(3-hydroxy-4-methylpent-4-en-1-yl)-2-methyl-7-pentyl-2H-chromen-5-ol*

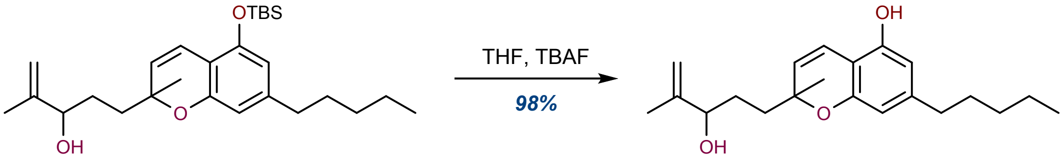

To a solution of 2-(3-hydroxy-4-methylpent-4-en-1-yl)-2-methyl-7-pentyl-2H-chromen-5-ol (100 mg, 0.225 mmol, 1 eq.) in THF (4.5 mL, 0.05M) was added a 0.1M solution of TBAF (248 µL, 0.248 mmol, 1.1 eq.) in THF. Reaction was left to stir overnight or until consumption of starting material. Reaction was quenched with saturated aq. ammonium chloride (8 mL) and extracted with EtOAc (3 × 10 mL). Extract was then dried with $MgSO_4$, filtered, and reconcentrated. The crude oil was purified by flash column chromatography using an eluent system of 5:1 then 4:1 Hexane:EtOAc. Product was isolated as a greenish oil (73 mg, 0.221 mmol, 98%).

R_f – 0.4 (3:1 hexane:EtOAc)

^{1}HNMR – ^{1}H NMR (500 MHz, $CDCl_3$) δ 6.64 (d, J = 10.0 Hz, 1H), 6.23 (s, 1H), 6.12 (s, 1H), 5.69 – 5.58 (m, 2H), 5.46 (d, J = 10.0 Hz, 1H), 5.21 – 5.17 (s, 1H), 2.47 – 2.30 (m, 4H), 1.58 – 1.50 (m, 3H), 1.41 (s, 3H), 1.34 – 1.27 (m, 5H), 1.22 (d, J = 8.5 Hz, 5H), 0.88 (t, J = 6.9 Hz, 3H).

^{13}CNMR - ^{13}C NMR (126 MHz, $CDCl_3$) δ 153.91, 151.38, 147.32-147.18, 144.95, 127.05-126.88, 117.31-117.30, 111.54-111.31, 109.03-109.01, 107.99-107.06, 106.99, 78.29-78.11, 76.20-76.05, 37.15-36.94, 36.03, 31.61, 30.76, 29.55-29.27, 26.61-26.36, 22.66, 17.84-17.68, 14.15.

HRMS – (ES+, m/z) [M+H]+ calcd. for $C_{21}H_{31}O_3$, 331.2273; found, 331.2261

IR – (ATR, neat, cm1): 3352 (br), 2955 (m), 2928 (m), 2857 (s), 1706 (s), 1622 (m), 1577 (m), 1430 (m), 1057 (m)

Synthesis of *(E)-2-(5-hydroxy-4-methylpent-3-en-1-yl)-2-methyl-7-pentyl-2H-chromen-5-ol*

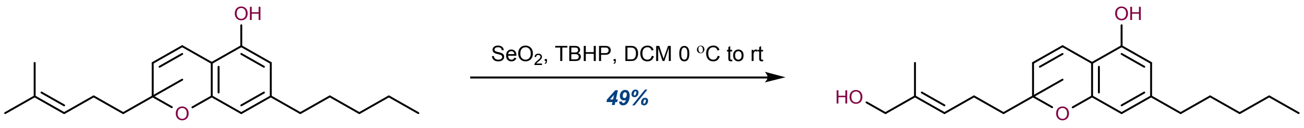

A solution of 5M TBHP in decane (337 µL, 1.69 mmol, 3.6 eq) and SeO_2 (10 mg, 0.09 mmol, 0.2 eq.) in DCM (3.3 mL, 0.14M) was stirred for 2 minutes at 0°C. A solution of CBC (147 mg, 0.468 mmol, 1 eq.) in DCM (3.3 mL, 0.14M) was added dropwise slowly to the stirring solution. Solution was allowed to stir and warmup to room temperature for 3.5 hours or until consumption of starting material by TLC. Reaction was filtered through a plug of celite and reconcentrated under reduced pressure. Crude material was purified by flash column chromatography using a gradient of 4:1 to 3:1 to 2:1 hexane:EtOAc. Product was isolated as a dark reddish oily residue (76 mg, 0.23 mmol, 49%).

R_f – 0.4 (3:1 hexane: EtOAc)

^{1}HNMR – ^{1}H NMR (500 MHz, $CDCl_3$) δ 6.64 (d, J = 9.9 Hz, 1H), 6.22 (s, 1H), 6.12 (s, 1H), 5.93 (s, 1H), 5.45 (d, J = 10.1 Hz, 1H), 5.39 (t, J = 7.4 Hz, 1H), 3.97 (s, 2H), 2.42 (t, J = 7.7 Hz, 2H), 2.23 – 2.10 (m, 2H), 1.80 – 1.64 (m, 2H), 1.62 (s, 3H), 1.55 (p, J = 7.5 Hz, 2H), 1.38 (s, 3H), 1.35 – 1.24 (m, 5H), 0.88 (t, J = 6.7 Hz, 3H).

^{13}CNMR - ^{13}C NMR (126 MHz, $CDCl_3$) δ 152.89, 150.41, 143.73, 133.43, 125.62, 125.52, 116.19, 107.62, 106.76, 105.90, 77.12, 68.01, 39.67, 34.89, 30.46, 29.61, 25.52, 24.18-18.78, 13.00, 12.56, -0.01.

HRMS – (ES+, m/z) [M-OH]+ calcd. for $C_{21}H_{29}O_2$, 313.2168; found, 313.2159

IR – (ATR, neat, cm1): 331.30 (br), 2957.10 (s), 2927.05 (m), 2857.20 (s), 1622.26 (m), 1576.94 (s), 1430.11 (m), 1077.72 (m)

Synthesis of *(1-(3,5-bis((tert-butyldimethylsilyl)oxy)phenyl)pentan-1-ol)*

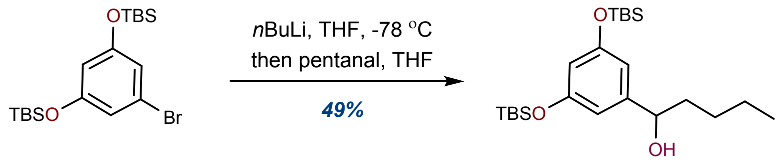

((5-bromo-1,3-phenylene)bis(oxy))bis(tert-butyldimethylsilane) (1.08g, 2.59mmol, 1 eq.) was suspended in THF (26 mL, 0.1M) allowed to cool to -78 ˚C under N_2 atmosphere. A 1.6M solution of nBuLi in hexanes (1.94 mL, 3.11mmol, 1.2 eq.) was slowly added to reaction vessel and allowed to stir for 10 minutes. Next, a solution of pentanal (410 µL, 3.89mmol, 1.5 eq.) in THF (3.9 mL, 1.0 M) was added dropwise to the reaction vessel and allowed to stir for 2 minutes, before solution was removed from dry ice bath and allowed to warm to room temperature. Solution stirred for 1.5 h at room temperature, then cooled to 0 ˚C and added to a solution of saturated aqueous ammonium chloride (25 mL). Crude was extracted with EtOAc (3 × 20 mL), dried with magnesium sulfate, and reconcentrated under reduced pressure. Crude mixture was purified via flash column chromatography in 20:1 hexane:EtOAc. Pure product was isolated as a clear oil (543 mg, 1.28mmol, 49%) and the remaining material was isolated as recovered starting material.

R_f – 0.2 (15:1 hexane: EtOAc)

^{1}HNMR – ^{1}H NMR (500 MHz, $CDCl_3$) δ 6.45 (d, J = 2.2 Hz, 2H), 6.24 (t, J = 2.2 Hz, 1H), 4.53 (dd, J = 7.2, 6.0 Hz, 1H), 1.80 – 1.60 (m, 3H), 1.39 – 1.28 (m, 1H), 0.97 (s, 18H), 0.87 (t, J = 7.1 Hz, 3H), 0.19 (s, 12H).

^{13}CNMR - 126 MHz, $CDCl_3$) δ 156.68, 147.32, 111.27-111.15, 74.63, 38.80, 28.02, 25.84, 22.75, 18.37, 14.17, -4.24.

HRMS – (ES+, m/z) [M+H]+ calcd. for $C_{23}H_{45}O_3Si_2$, 425.2907; found, 425.2898

IR – (ATR, neat, cm1): 3378 (br), 2956 (s), 2930 (s), 2859 (s), 1590 (m), 1450 (m), 1253 (m), 1160 (m), 830 (m), 779 (m)

Synthesis of 5-(1-hydroxypentyl)benzene-1,3-diol

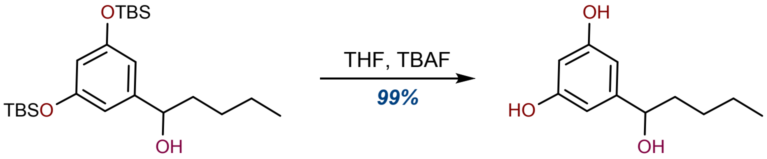

To a solution of (1-(3,5-bis((tert-butyldimethylsilyl)oxy)phenyl)pentan-1-ol) (138 mg, 0.326 mmol) in THF (6.5 mL, 0.05M) was added a 0.1M solution of TBAF (717 µL, 0.717 mmol, 2.2 eq.) in THF. Reaction was left to stir overnight or until consumption of starting material. Reaction was quenched with saturated aq. ammonium chloride (8 mL) and extracted with EtOAc (3 x 10 mL). Extract was then dried with $MgSO_4$, filtered, and reconcentrated. The crude material was filtered through a short plug of silica (4:1 then 1:1 hexane:EtOAc) then concentrated, to afford a clear oil (64 mg, 0.326 mmol, 99%) which was taken directly to the next step.

Synthesis of *7-(1-hydroxypentyl)-2-methyl-2-(4-methylpent-3-en-1-yl)-2H-chromen-5-ol*

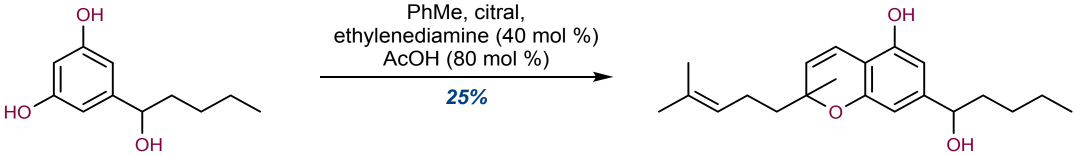

To a solution of (1-(3,5-bis((tert-butyldimethylsilyl)oxy)phenyl)pentan-1-ol) (64 mg, 0.326mmol, 1 eq.) and citral (67 µL, 0.391mmol, 1.2 eq.) in toluene (3.3 mL, 0.1M) was added ethylenediamine (9 µL, 0.13mmol, 0.4 eq.) at room temperature. The solution was then heated to reflux for 24 hours until consumption of starting arene. After completion, the solution was added to ammonium chloride (5 mL) and extracted with excess DCM (3 × 5 mL). The crude was dried with magnesium sulfate and concentrated under reduced pressure. The crude product was then purified by flash column chromatography using hexane:EtOAc, 8:1 to 4:1 to 1:1. Pure product was isolated as a clear oil (27 mg, 0.081mmol, 25%).

R_f – 0.4 (3:1 hexane:EtOAc)

^{1}HNMR – ^{1}H NMR (500 MHz, $CDCl_3$) δ 6.64 (d, J = 10.0 Hz, 1H), 6.40 (d, J = 7.3 Hz, 1H), 6.30 (d, J = 5.5 Hz, 1H), 6.26 (s, 1H), 5.51 (d, J = 10.0 Hz, 1H), 5.08 (t, J = 7.2 Hz, 1H), 4.47 (t, J = 6.7 Hz, 1H), 2.44 (s, 1H), 2.10 (q, J = 9.0, 7.7 Hz, 2H), 1.76 – 1.68 (m, 2H), 1.65 (s, 3H), 1.57 (s, 3H), 1.37 (d, J = 2.8 Hz, 3H), 1.35 – 1.19 (m, 4H), 0.86 (t, J = 7.0 Hz, 3H).

^{13}CNMR - ^{13}C NMR (126 MHz, $CDCl_3$) δ 155.91-152.01, 154.11-149.31, 145.86, 131.57, 127.68, 124.00, 114.87-118.77, 110.08-107.18, 113.23-99.73, 109.99-99.98, 78.26, 76.14-73.34, 40.94, 37.99, 27.79, 28.67-23.57, 25.55, 24.12-21.02, 22.43, 17.50, 13.86.

HRMS – (ES+, m/z) [M+H]+ calcd. for $C_{21}H_{31}O_3$, 331.2273; found, 331.2260

IR – (ATR, neat, cm1): 3339 (br), 2960 (m), 2929 (m), 2860 (s), 1623 (m), 1580 (s), 1433 (m), 1057 (m)

1.3 ^{1}H NMR and ^{13}C NMR spectra

All the NMR data have been uploaded to the archive

https://depositions.np-mrd.org/request-data/0b44814f-9e10-4cdf-ad51-29dfadee46a0

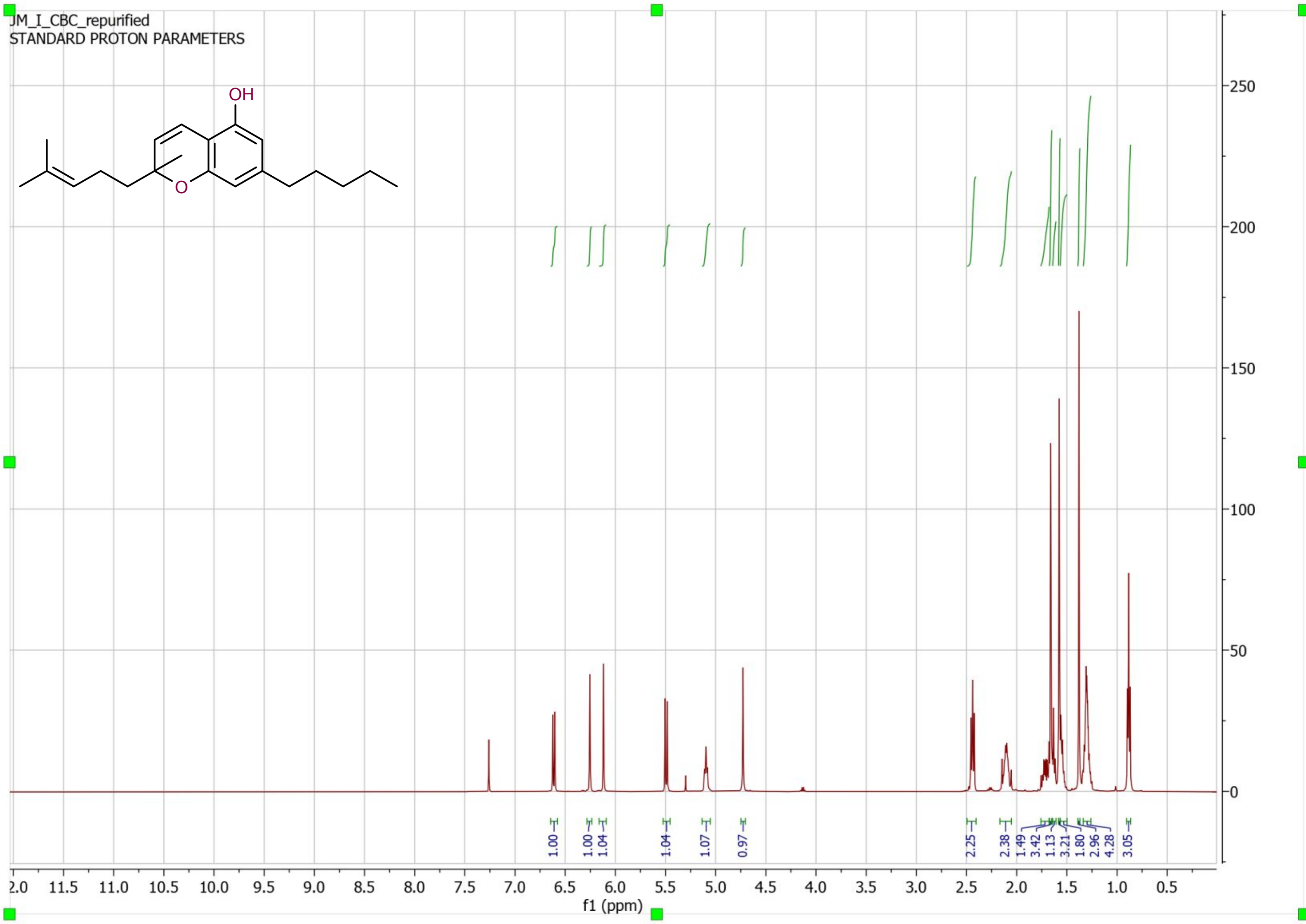

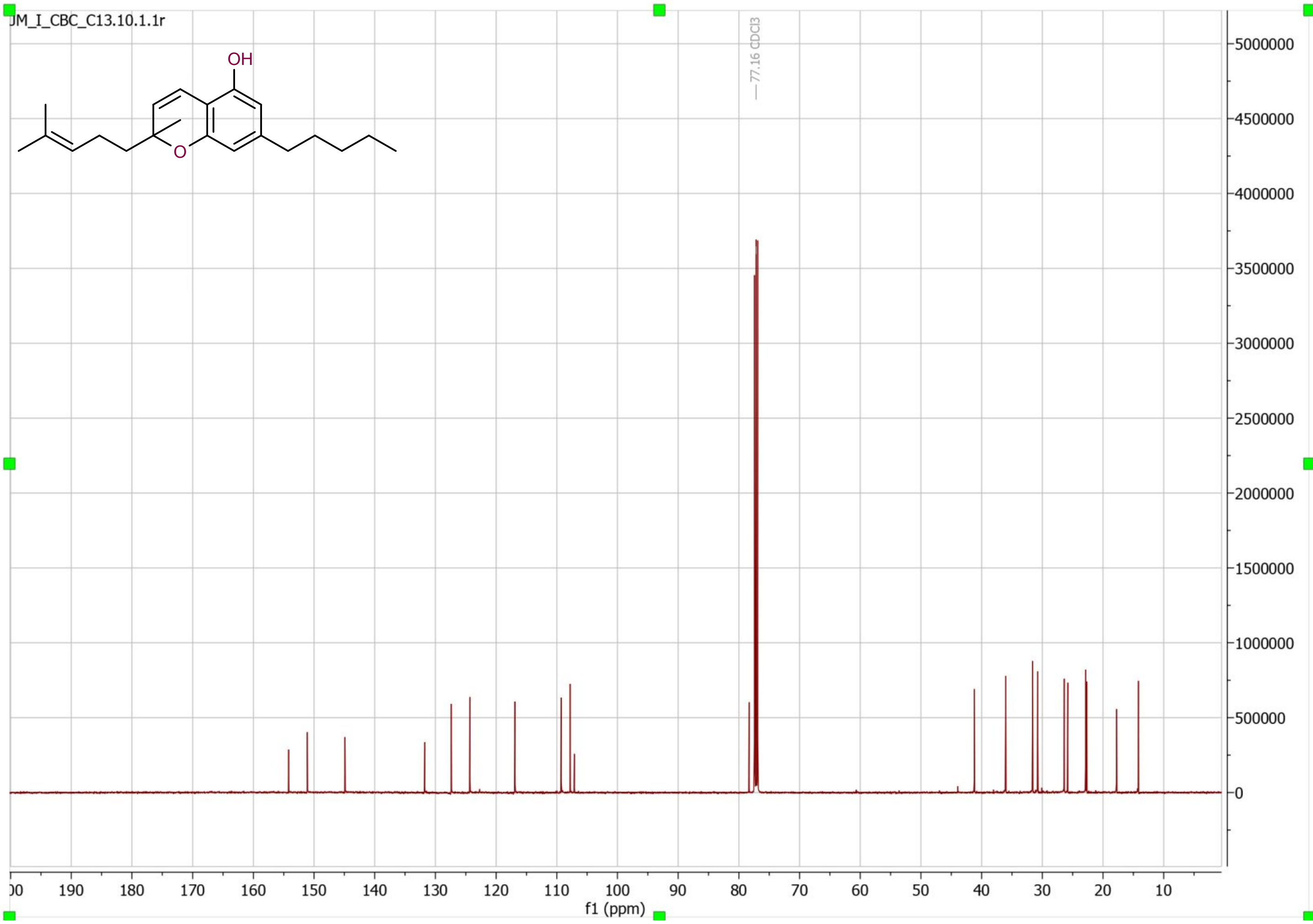
JM_I_CBC_C13.10.1.1r
OH
O
77.16 CDCl3
f1 (ppm)

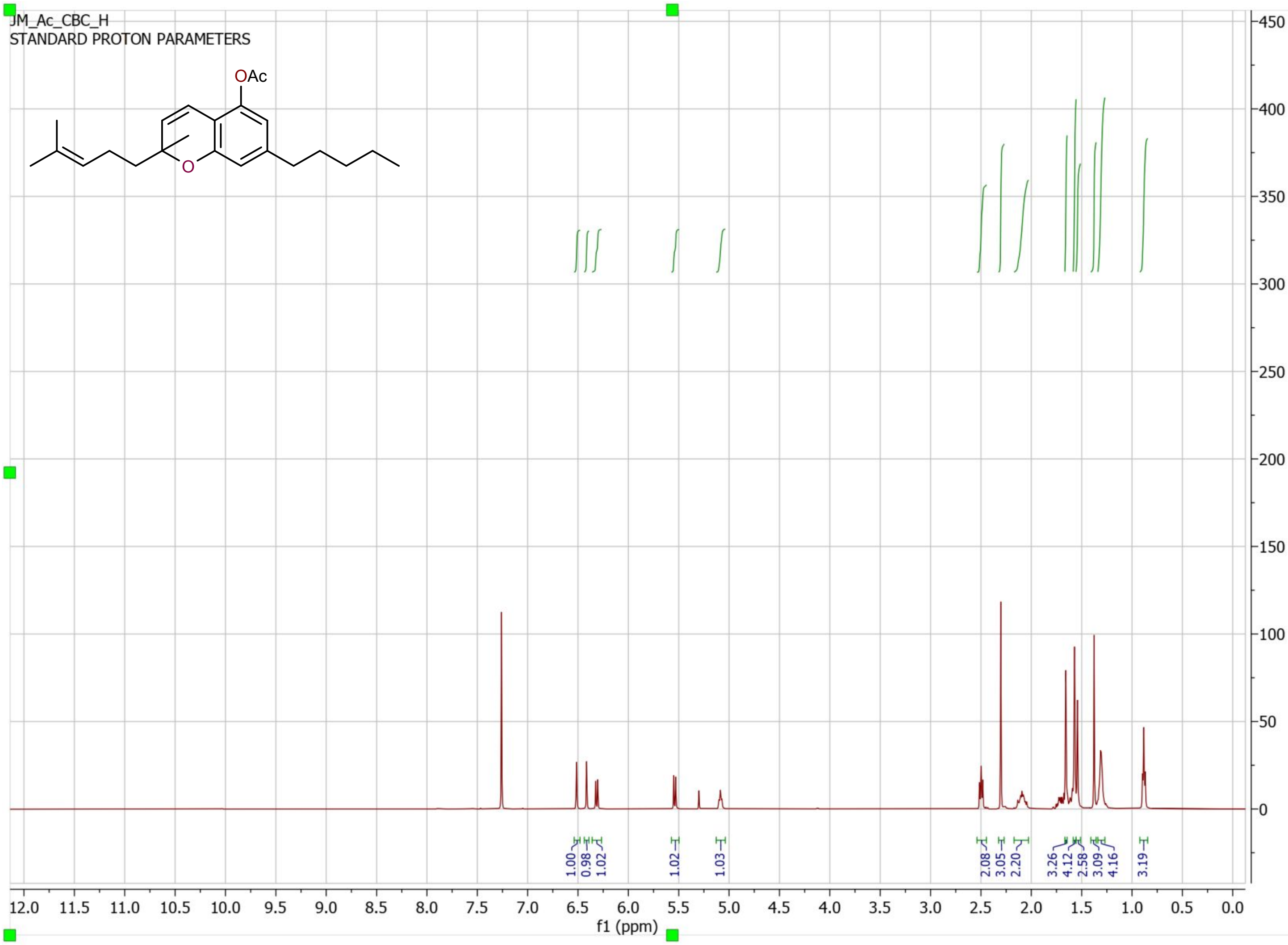

JM_Ac_CBC_H
STANDARD PROTON PARAMETERS
OAc
O
1.00
0.98
1.02
1.02
1.03
2.08
3.05
2.20
3.26
4.12
2.58
3.09
4.16
3.19
f1 (ppm)

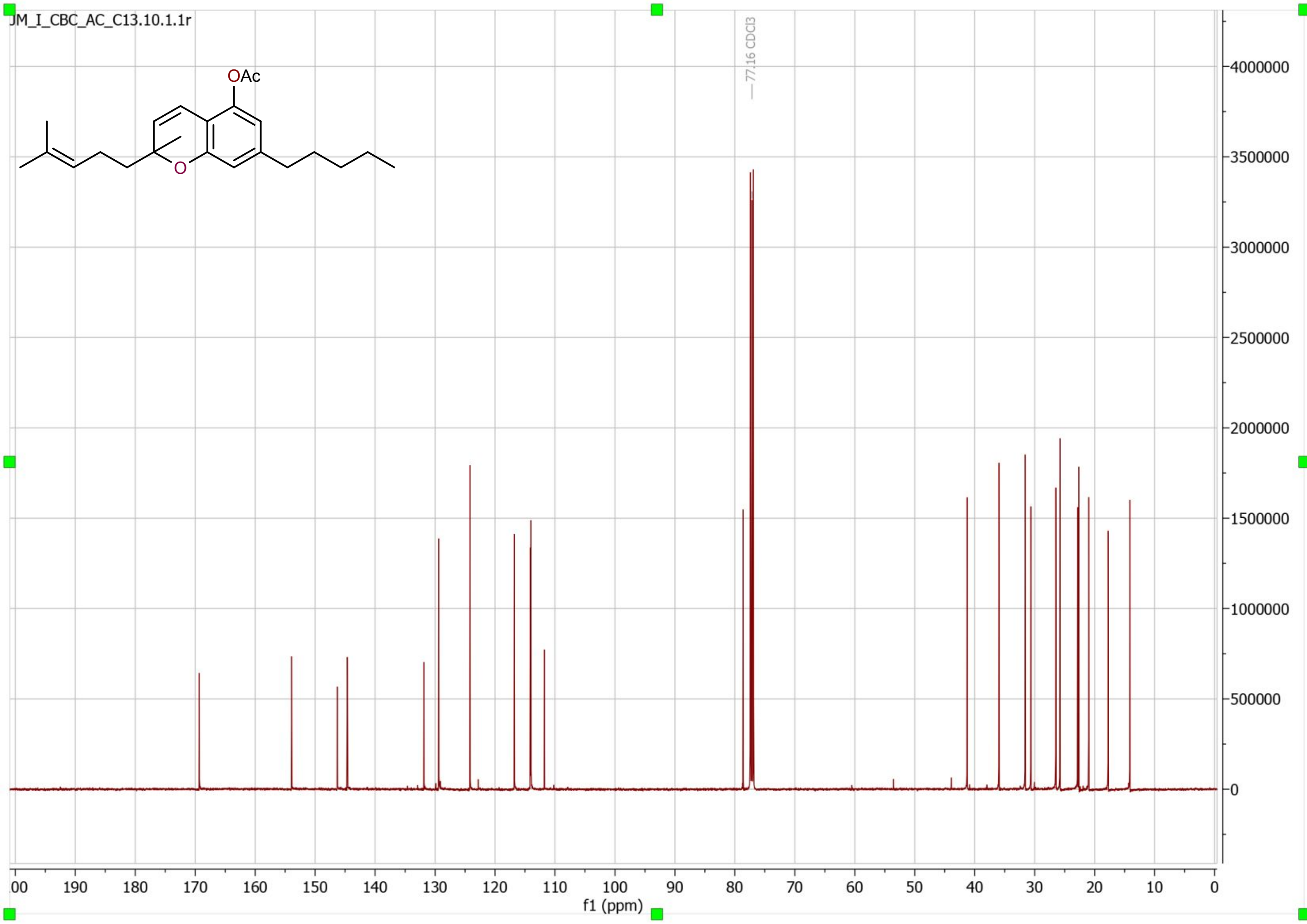

JM_I_CBC_AC_C13.10.1.1r
OAc
O
77.16 CDCl3
f1 (ppm)

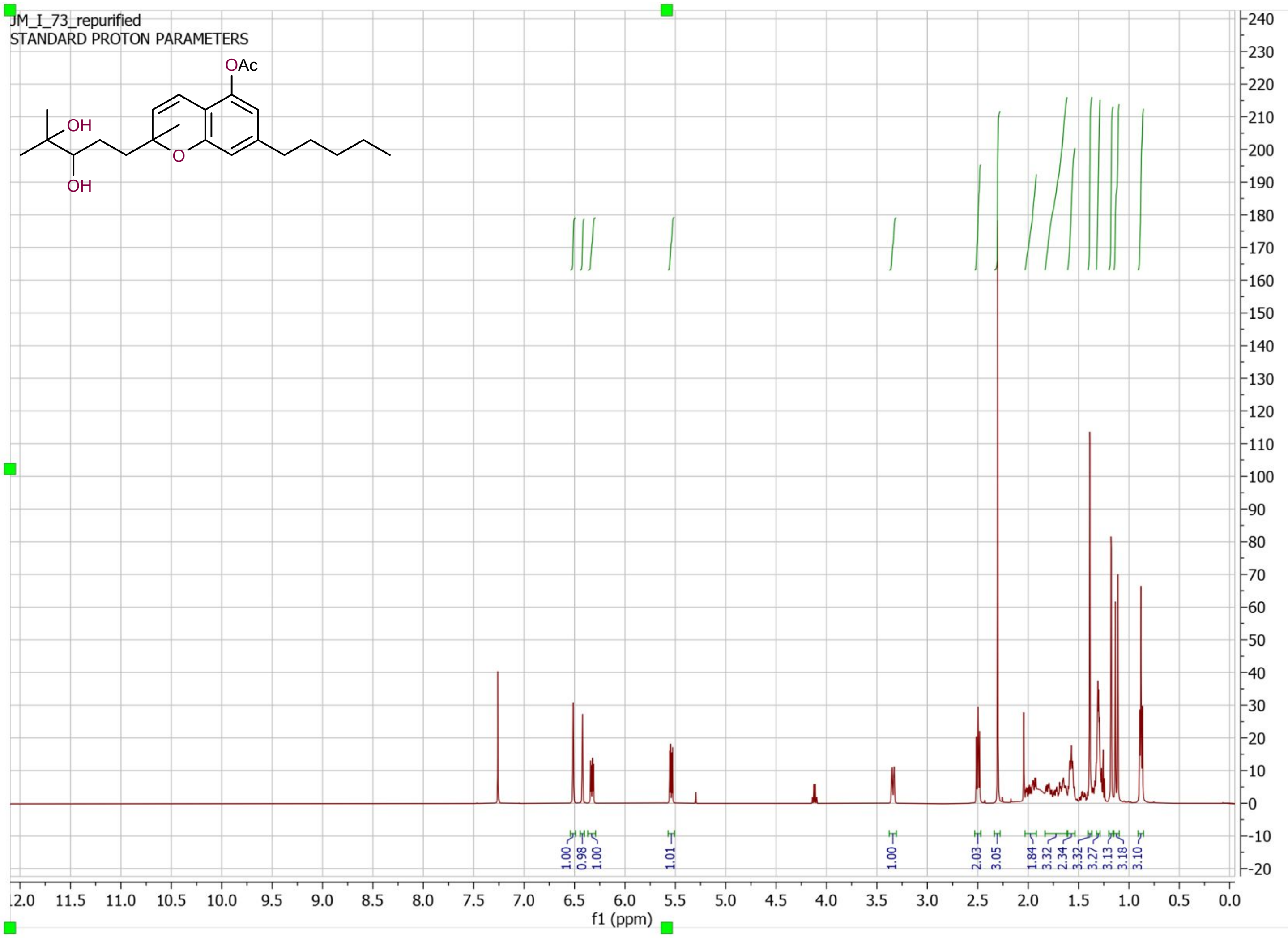
JM_I_73_repurified
STANDARD PROTON PARAMETERS
OAc
OH
OH
O
1.00
0.98
1.00
1.01
1.00
2.03
3.05
1.84
3.32
2.34
3.32
3.27
3.13
3.18
3.10
f1 (ppm)

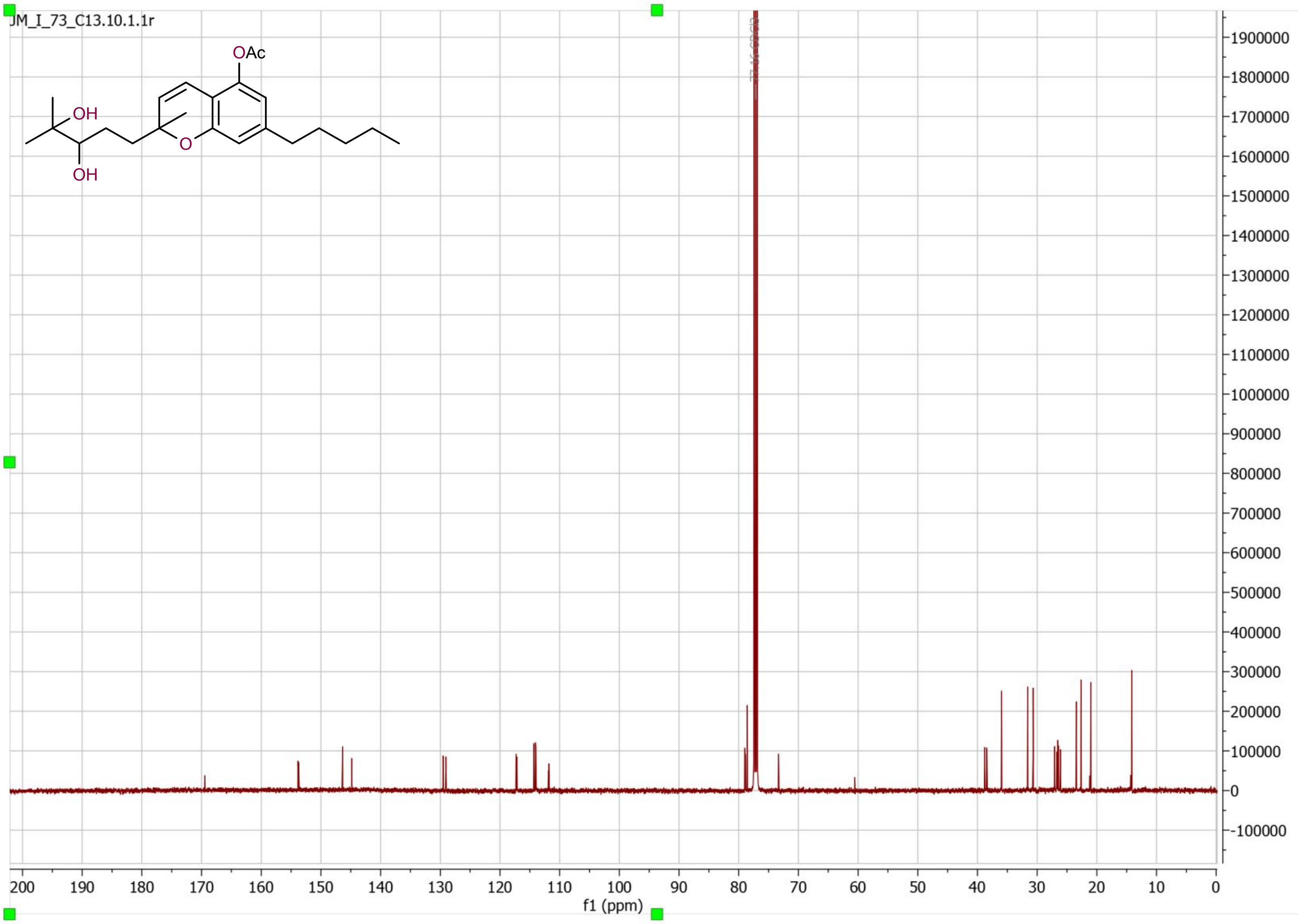
JM_I_73_C13.10.1.1r
OAc
OH
OH
O
f1 (ppm)

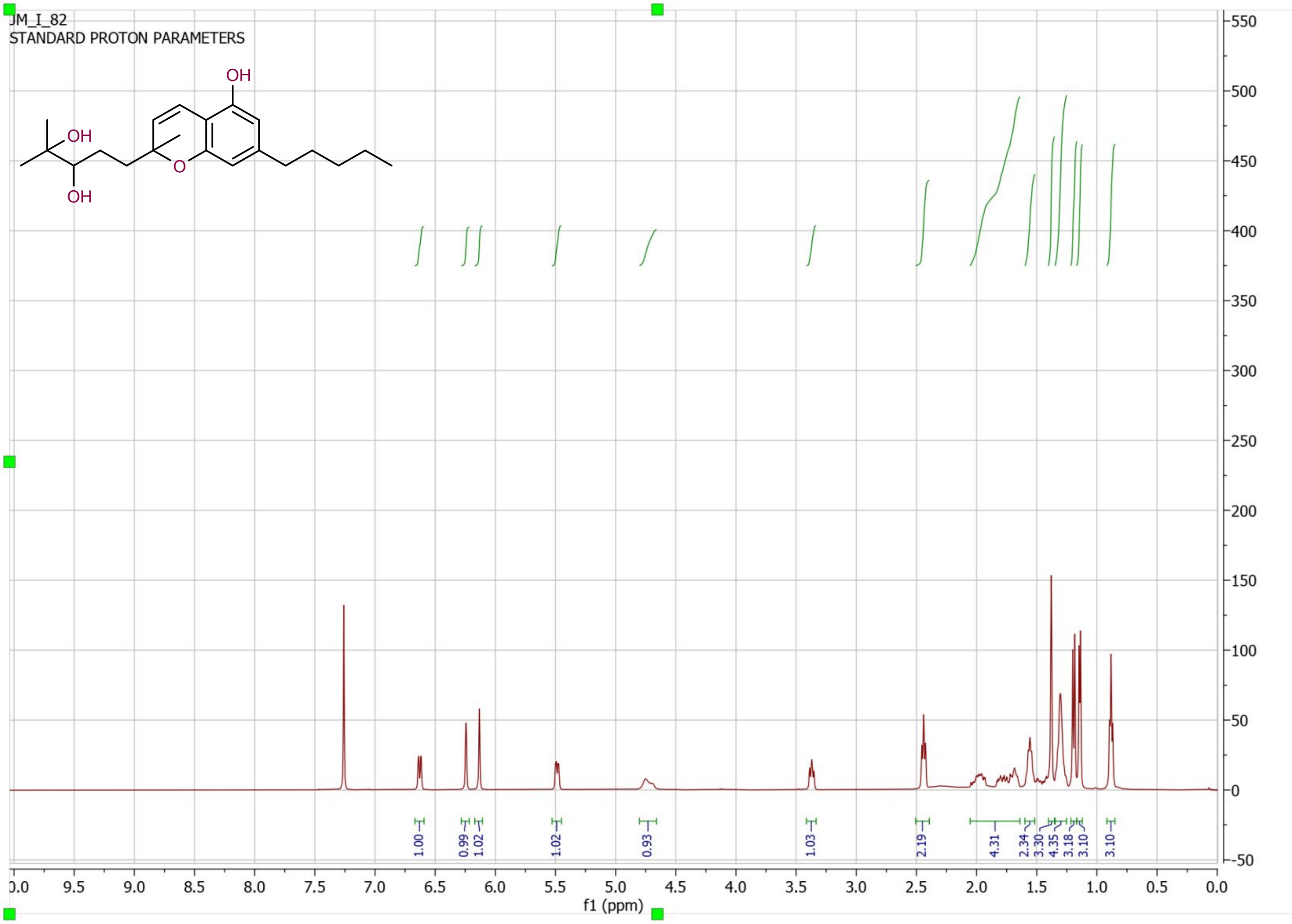
JM_I_82
STANDARD PROTON PARAMETERS
OH
OH
OH
O
1.00
0.99
1.02
1.02
0.93
1.03
2.19
4.31
2.34
3.30
4.35
3.18
3.10
3.10
f1 (ppm)

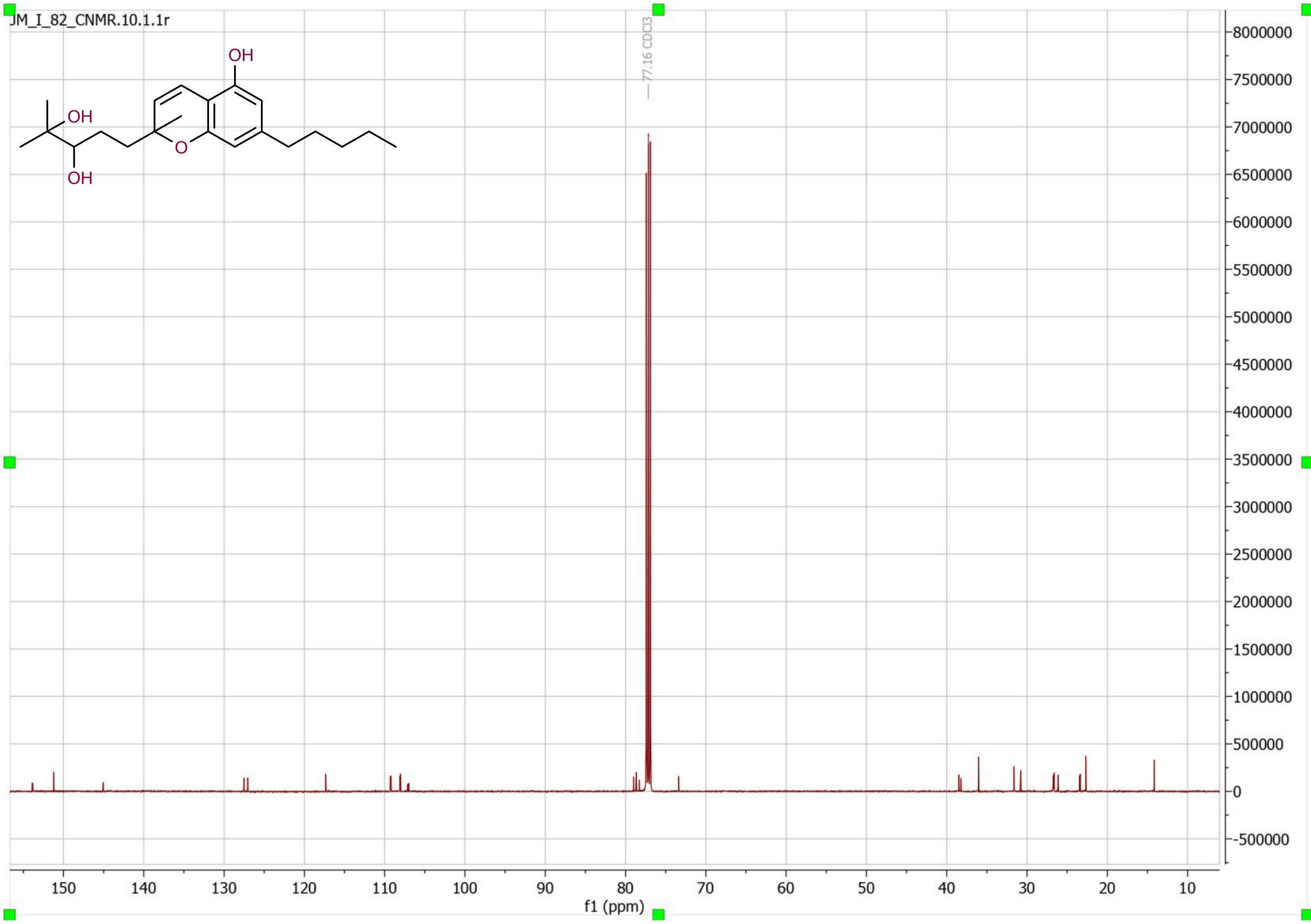
JM_I_82_CNMR.10.1.1r
77.16 CDCl3
OH
OH
OH
O
f1 (ppm)

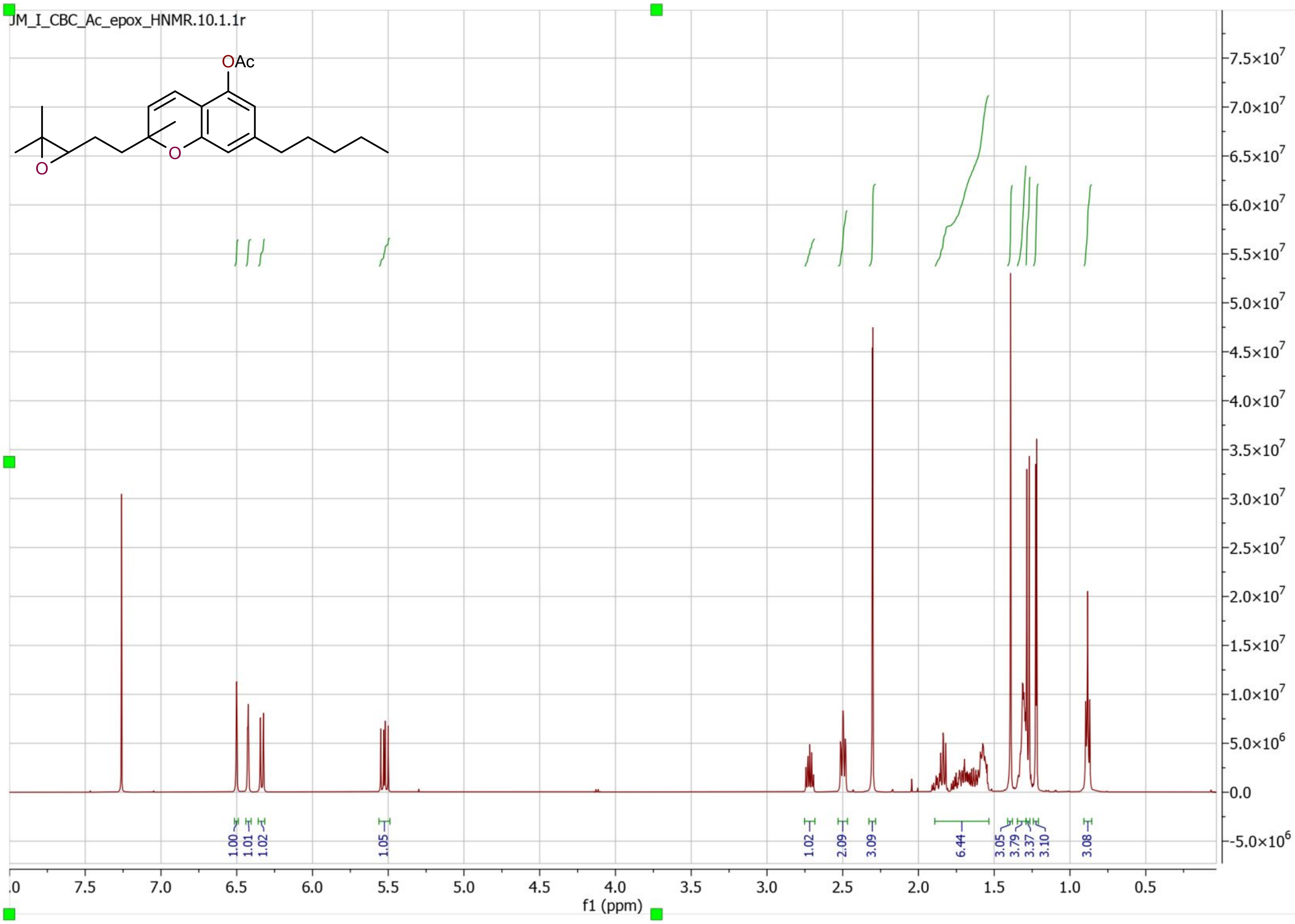
JM_I_CBC_Ac_epox_HNMR.10.1.1r
OAc
O
O
f1 (ppm)
1.00
1.01
1.02
1.05
1.02
2.09
3.09
6.44
3.05
3.79
3.37
3.10
3.08

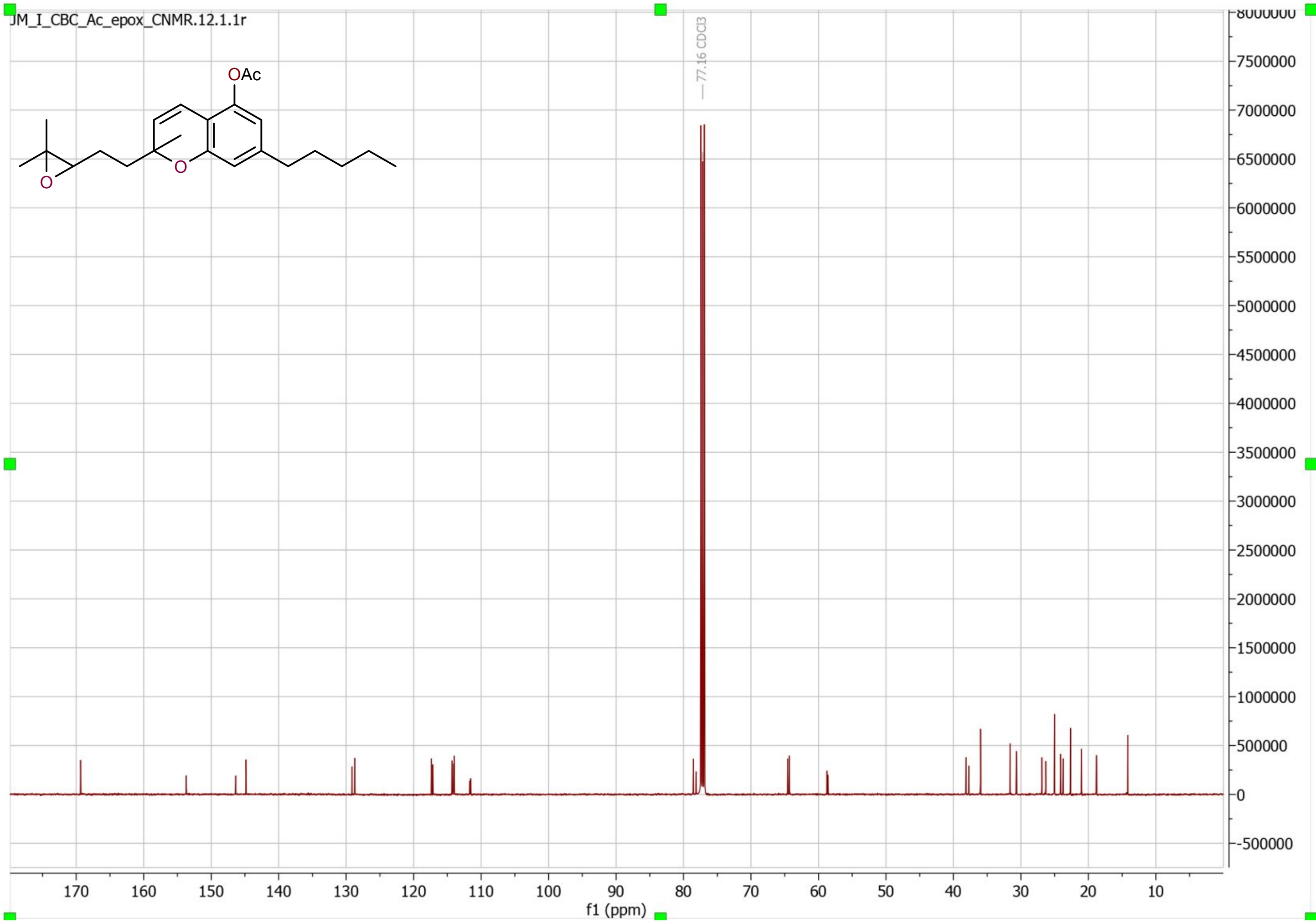

JM_I_CBC_Ac_epox_CNMR.12.1.1r
OAc
O
O
77.16 CDCl3
f1 (ppm)

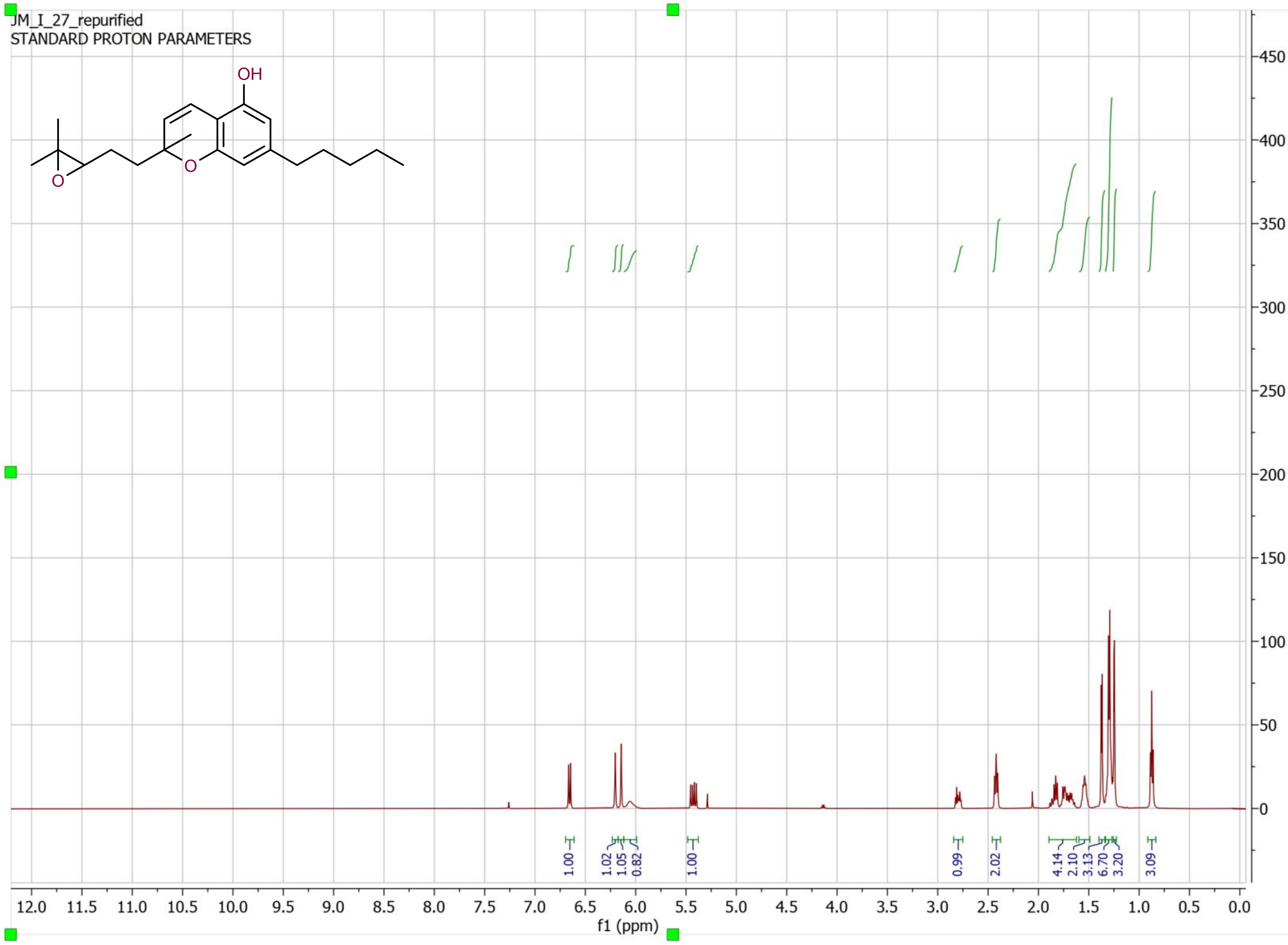
JM_I_27_repurified
STANDARD PROTON PARAMETERS
OH
O
O
1.00
1.02
1.05
0.82
1.00
0.99
2.02
4.14
2.10
3.13
6.70
3.20
3.09
12.0 11.5 11.0 10.5 10.0 9.5 9.0 8.5 8.0 7.5 7.0 6.5 6.0 5.5 5.0 4.5 4.0 3.5 3.0 2.5 2.0 1.5 1.0 0.5 0.0
f1 (ppm)
450
400
350
300
250
200
150
100
50
0

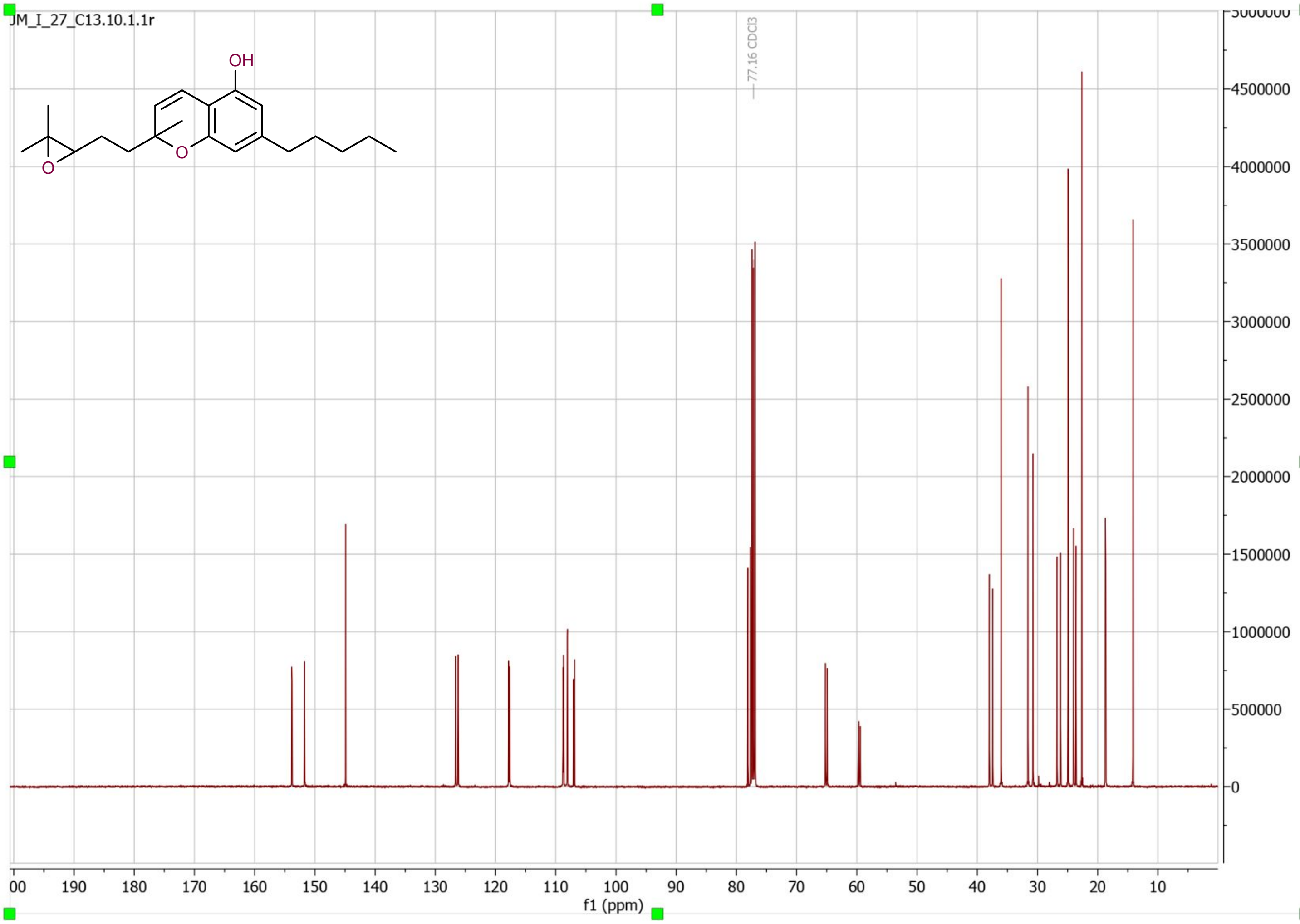
JM_I_27_C13.10.1.1r
OH
O
O
77.16 CDCl3
f1 (ppm)
5000000
4500000
4000000
3500000
3000000
2500000
2000000
1500000
1000000
500000
0
00
190
180
170
160
150
140
130
120
110
100
90
80
70
60
50
40
30
20
10

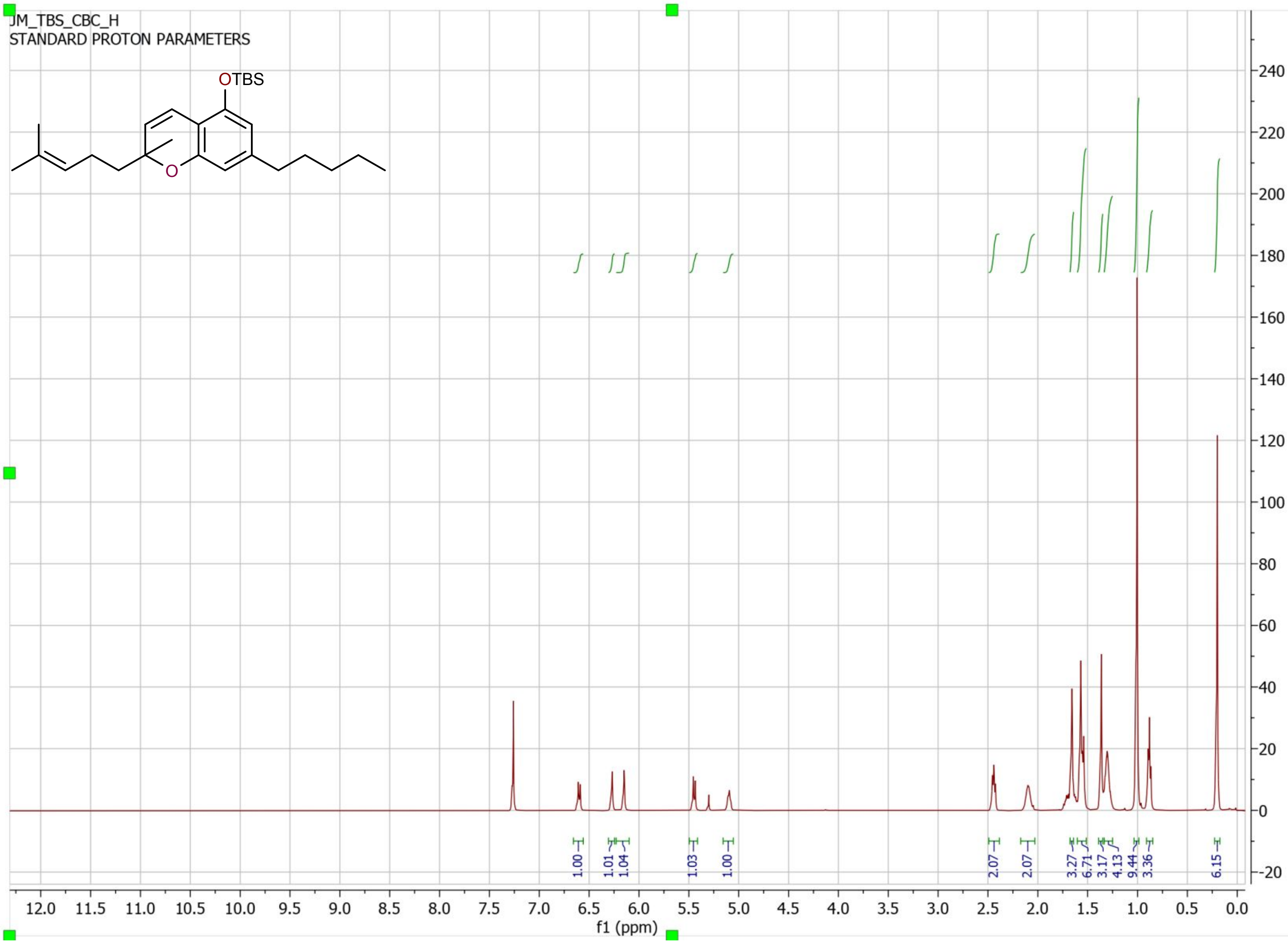

JM_TBS_CBC_H
STANDARD PROTON PARAMETERS
OTBS
O
1.00
1.01
1.04
1.03
1.00
2.07
2.07
3.27
6.71
3.17
4.13
9.44
3.36
6.15
f1 (ppm)

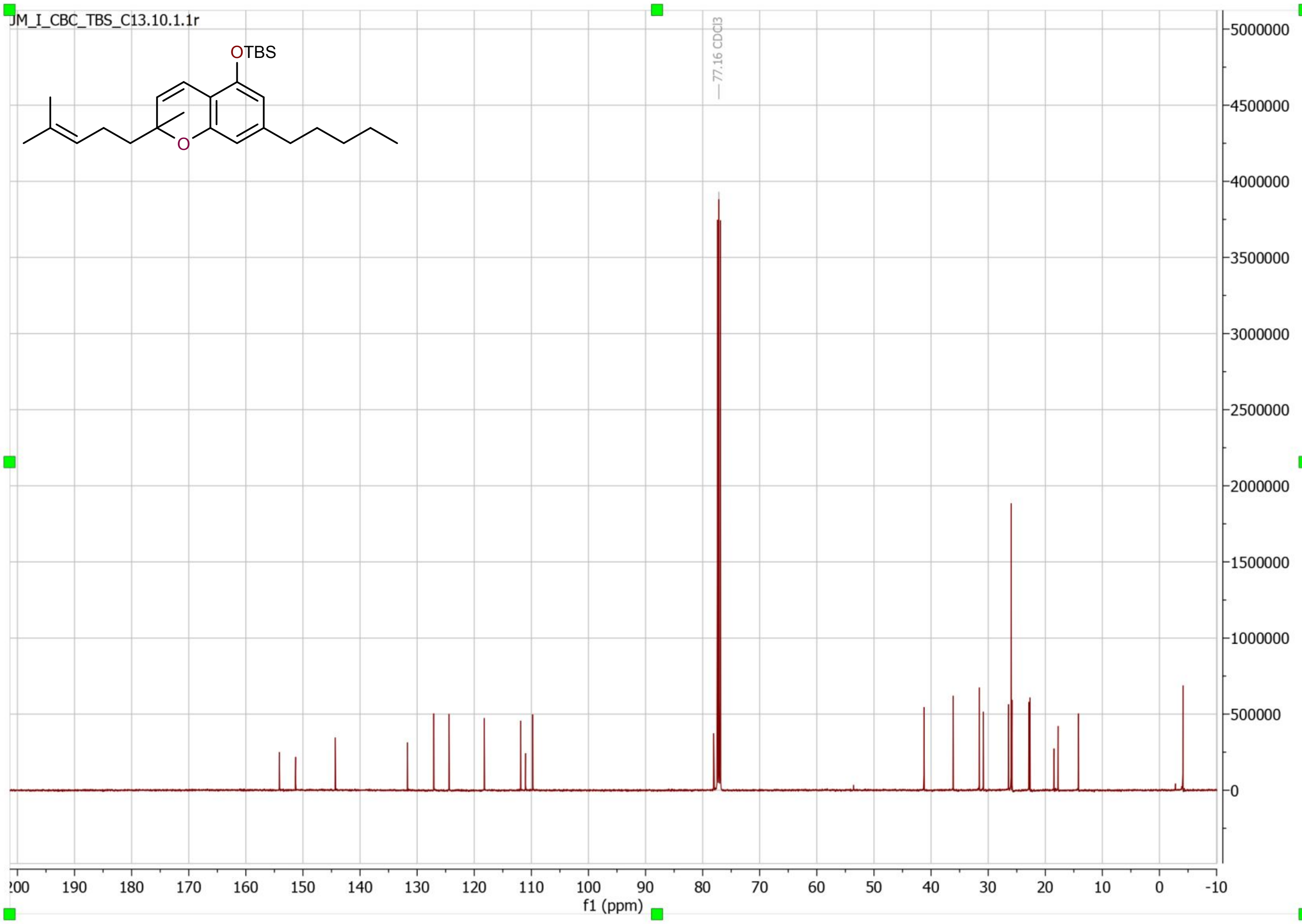

JM_I_CBC_TBS_C13.10.1.1r
OTBS
O
77.16 CDCl3
f1 (ppm)

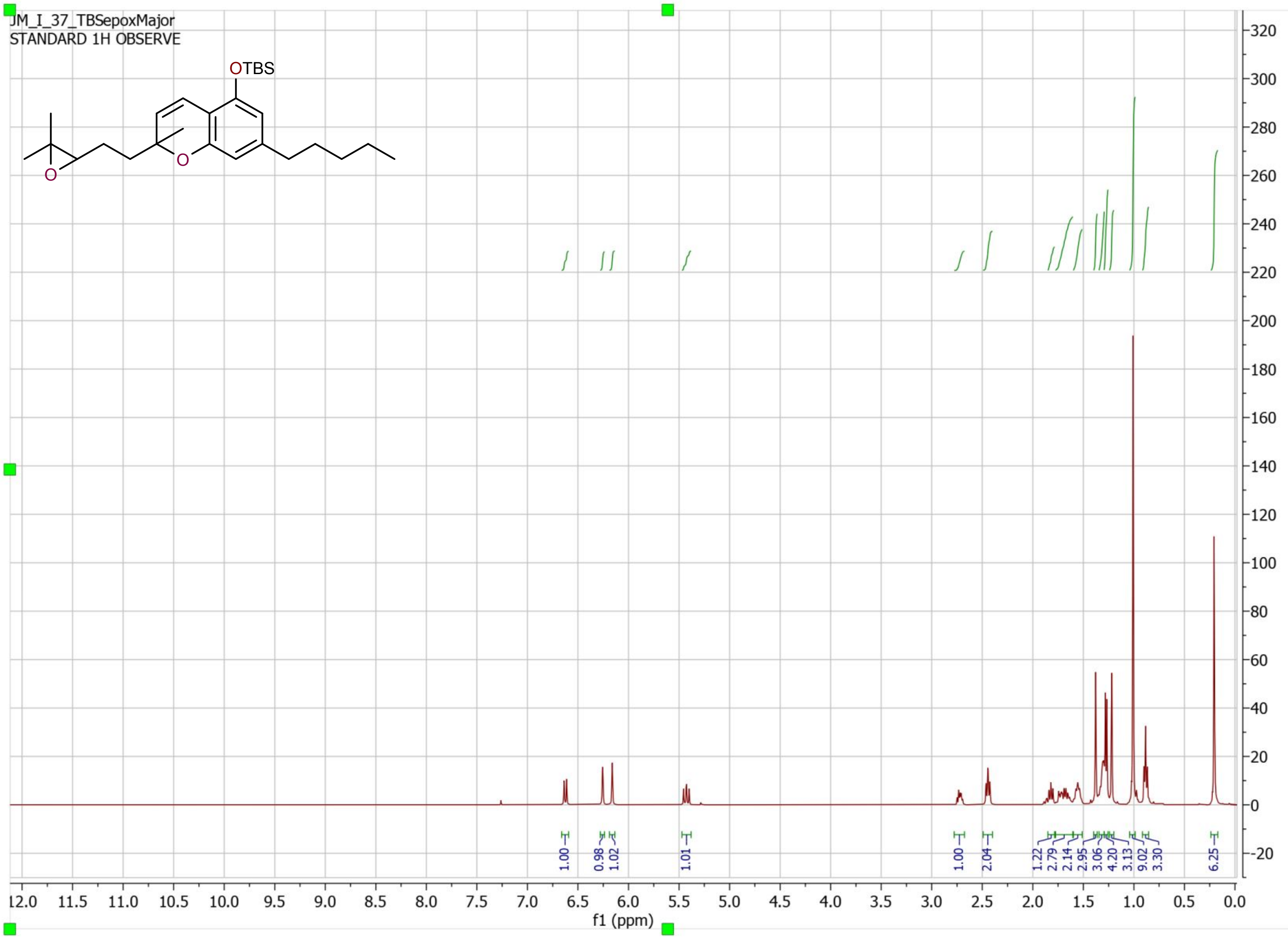

JM_I_37_TBSepoxMajor
STANDARD 1H OBSERVE
OTBS
O
O
1.00
0.98
1.02
1.01
1.00
2.04
1.22
2.79
2.14
2.95
3.06
4.20
3.13
9.02
3.30
6.25
f1 (ppm)

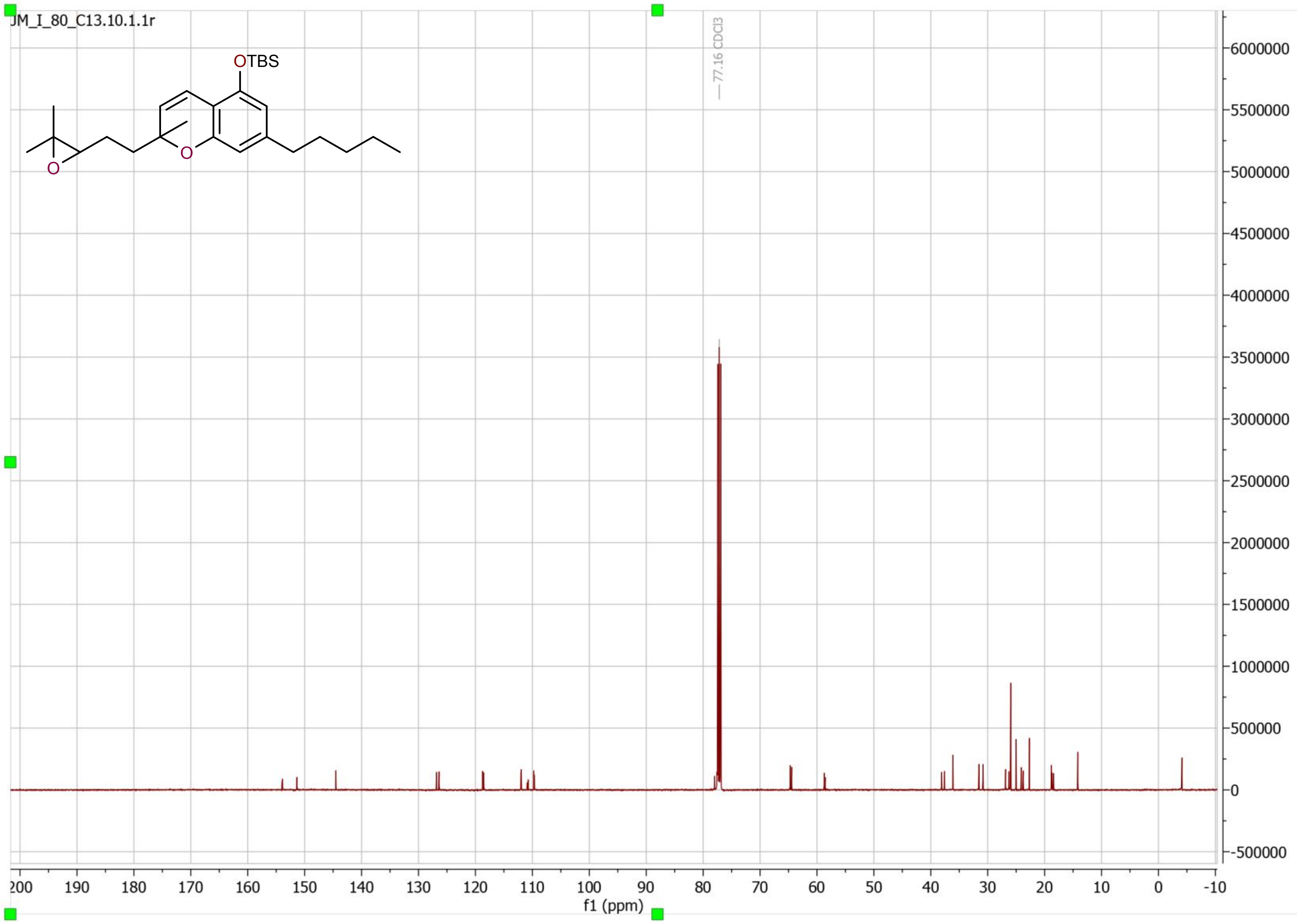

JM_I_80_C13.10.1.1r
OTBS
O
O
77.16 CDCl3
f1 (ppm)

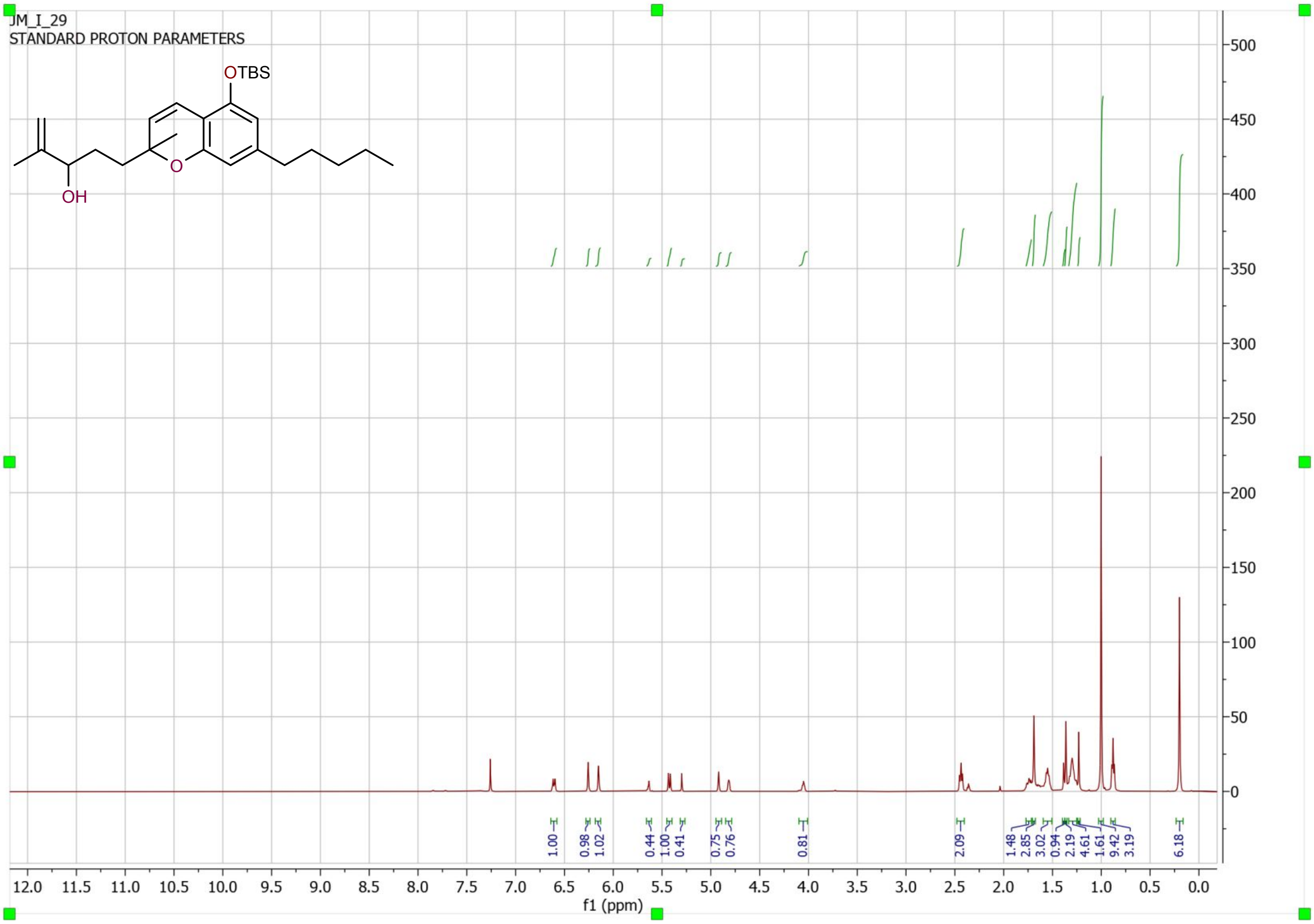
JM_I_29
STANDARD PROTON PARAMETERS
OTBS
O
OH
f1 (ppm)

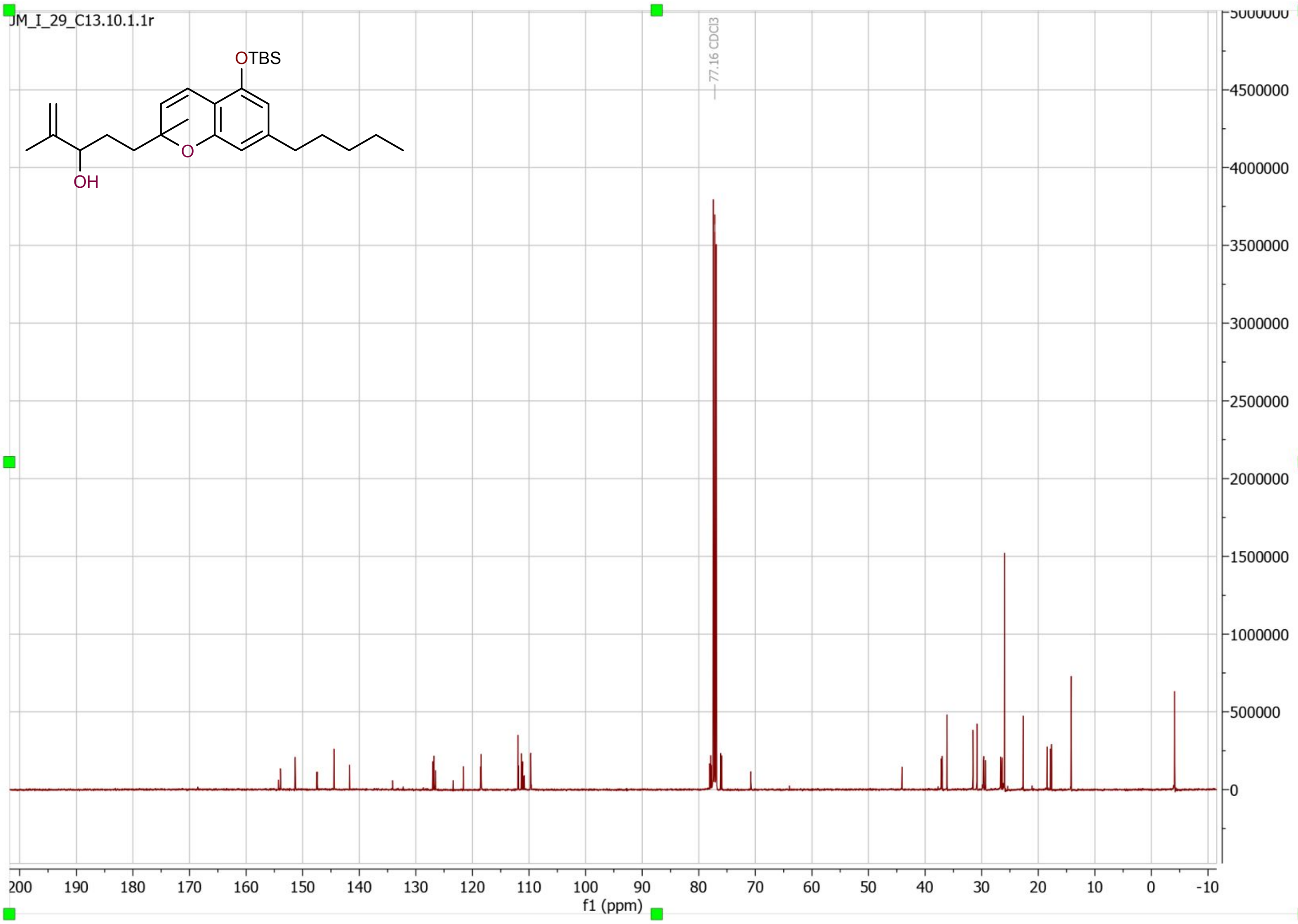
JM_I_29_C13.10.1.1r
OTBS
O
OH
77.16 CDCl3
5000000
4500000
4000000
3500000
3000000
2500000
2000000
1500000
1000000
500000
0
200 190 180 170 160 150 140 130 120 110 100 90 80 70 60 50 40 30 20 10 0 -10
f1 (ppm)

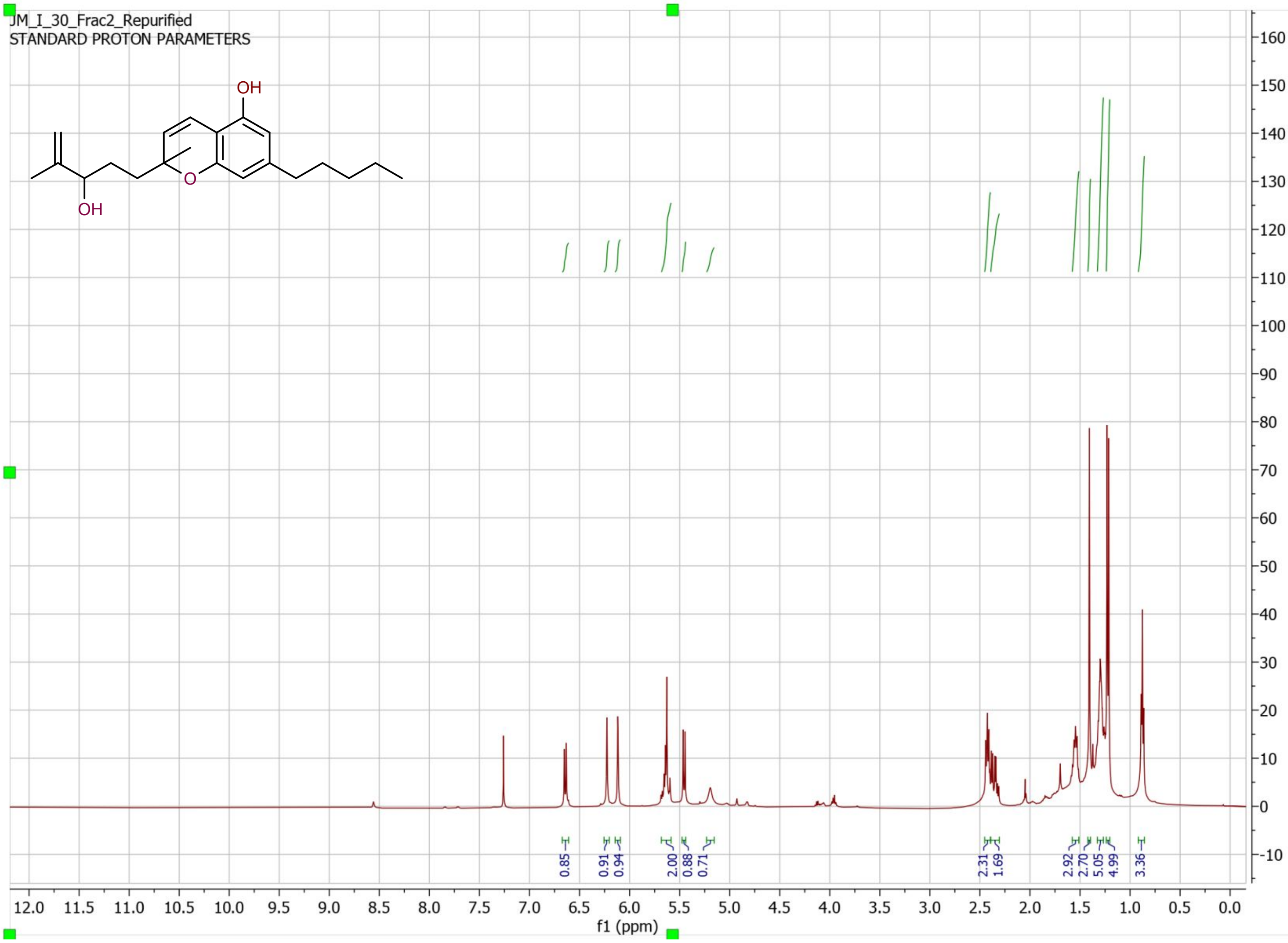
JM_I_30_Frac2_Repurified
STANDARD PROTON PARAMETERS
OH
O
OH
0.85
0.91
0.94
2.00
0.88
0.71
2.31
1.69
2.92
2.70
5.05
4.99
3.36
f1 (ppm)

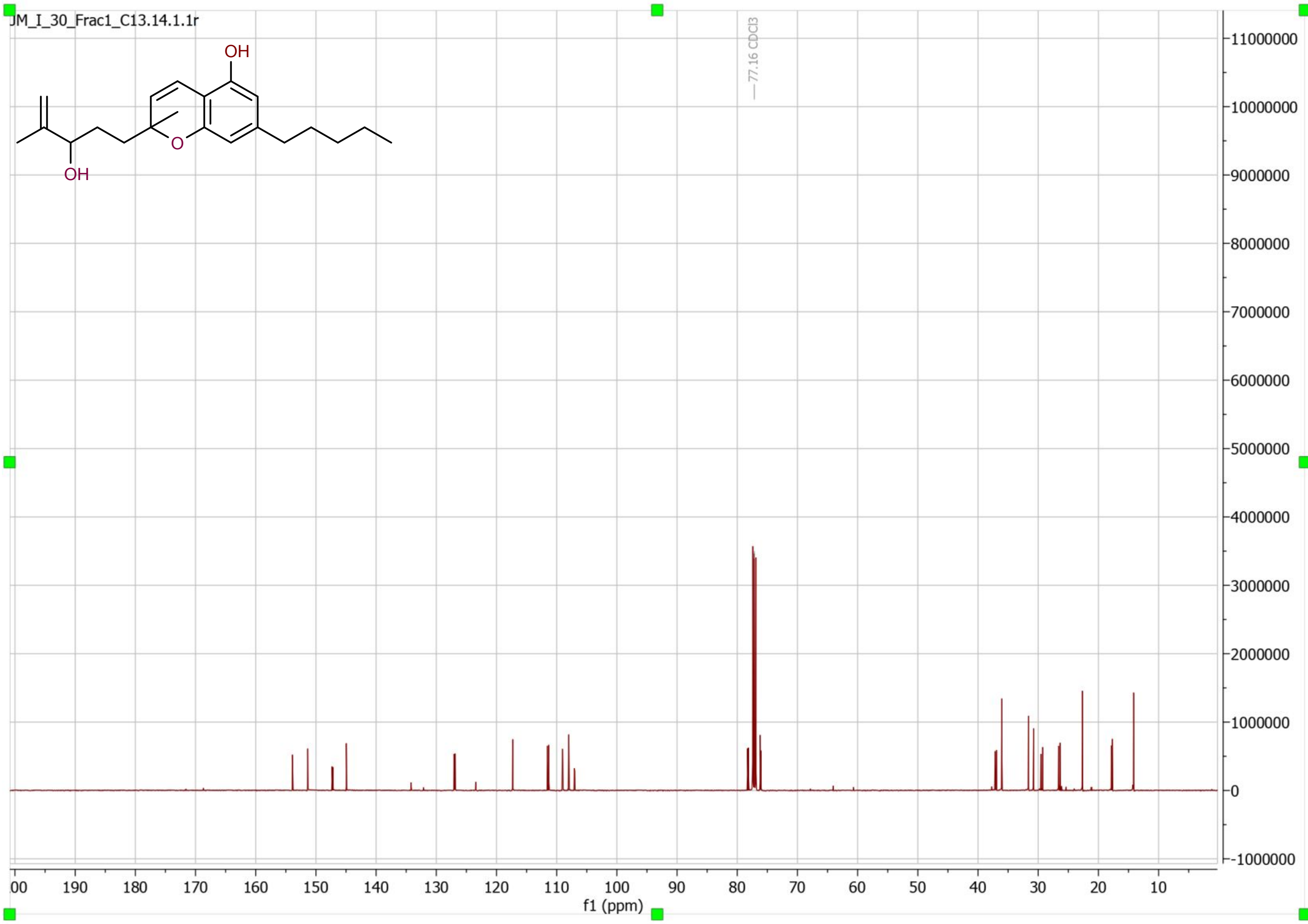
JM_I_30_Frac1_C13.14.1.1r
OH
OH
O
77.16 CDCl3
f1 (ppm)

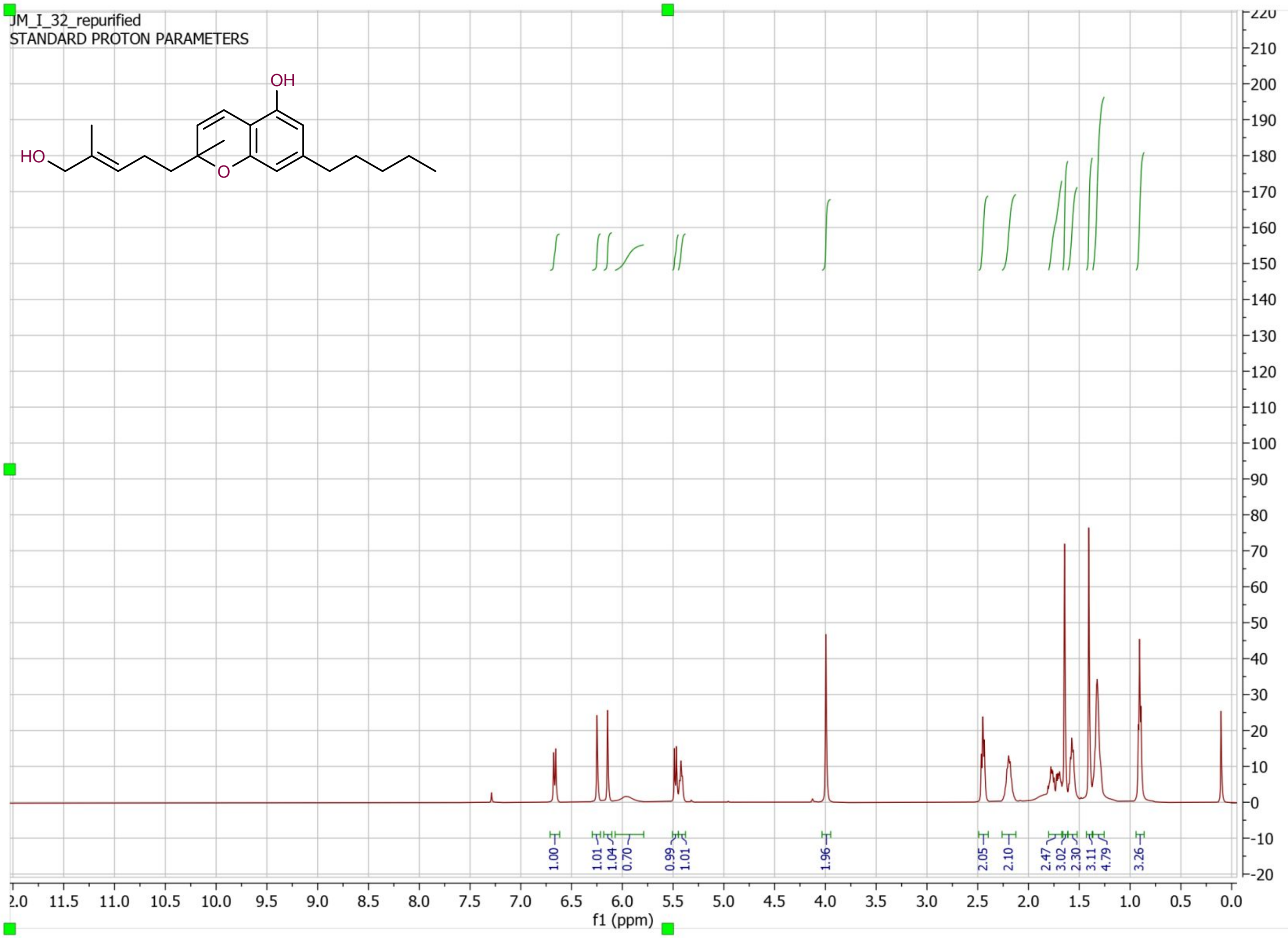

JM_I_32_repurified
STANDARD PROTON PARAMETERS
OH
HO
O
1.00
1.01
1.04
0.70
0.99
1.01
1.96
2.05
2.10
2.47
3.02
2.30
3.11
4.79
3.26
f1 (ppm)

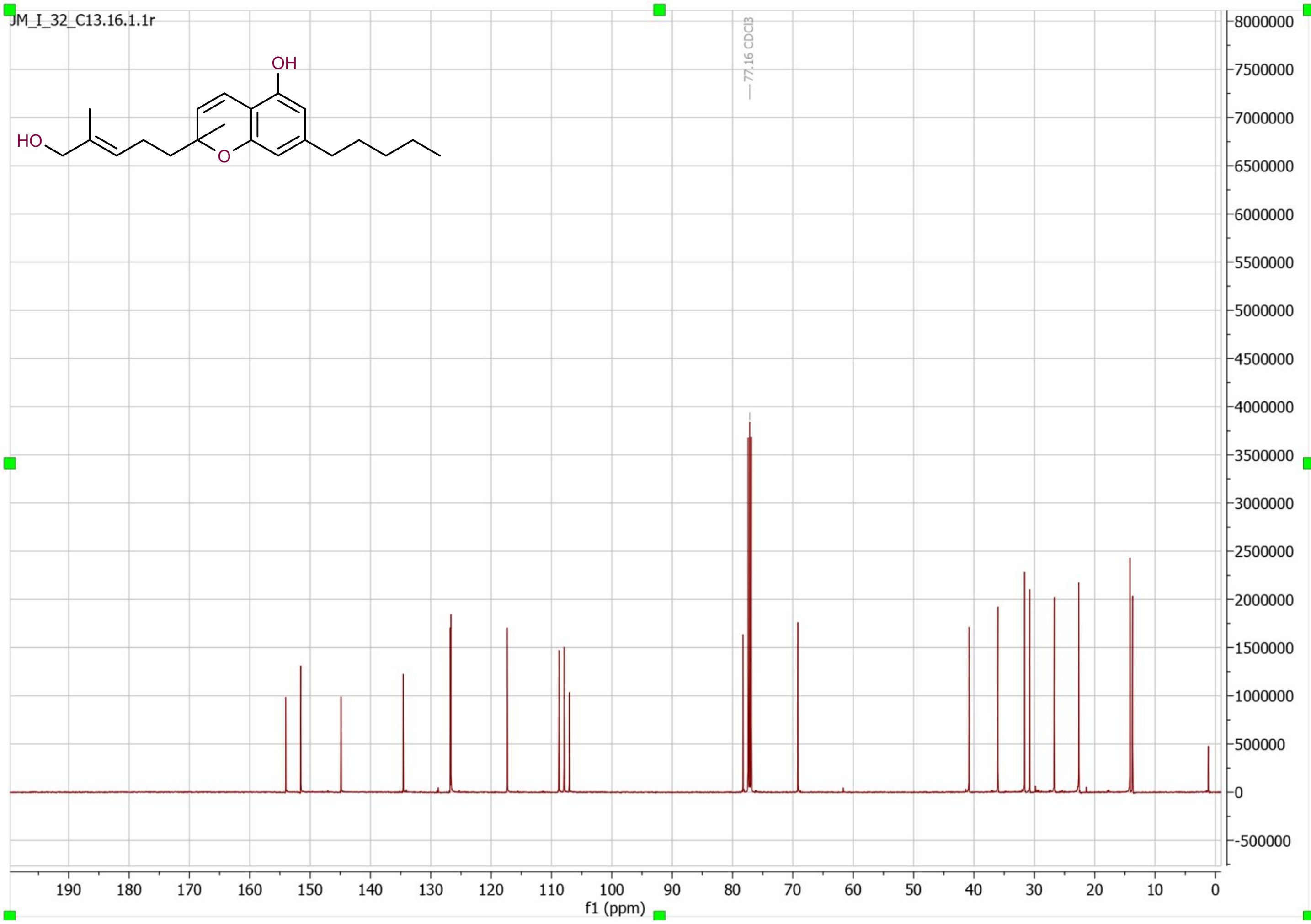

JM_I_32_C13.16.1.1r
OH
HO
O
77.16 CDCl3
8000000
7500000
7000000
6500000
6000000
5500000
5000000
4500000
4000000
3500000
3000000
2500000
2000000
1500000
1000000
500000
0
-500000
190
180
170
160
150
140
130
120
110
100
90
80
70
60
50
40
30
20
10
0
f1 (ppm)

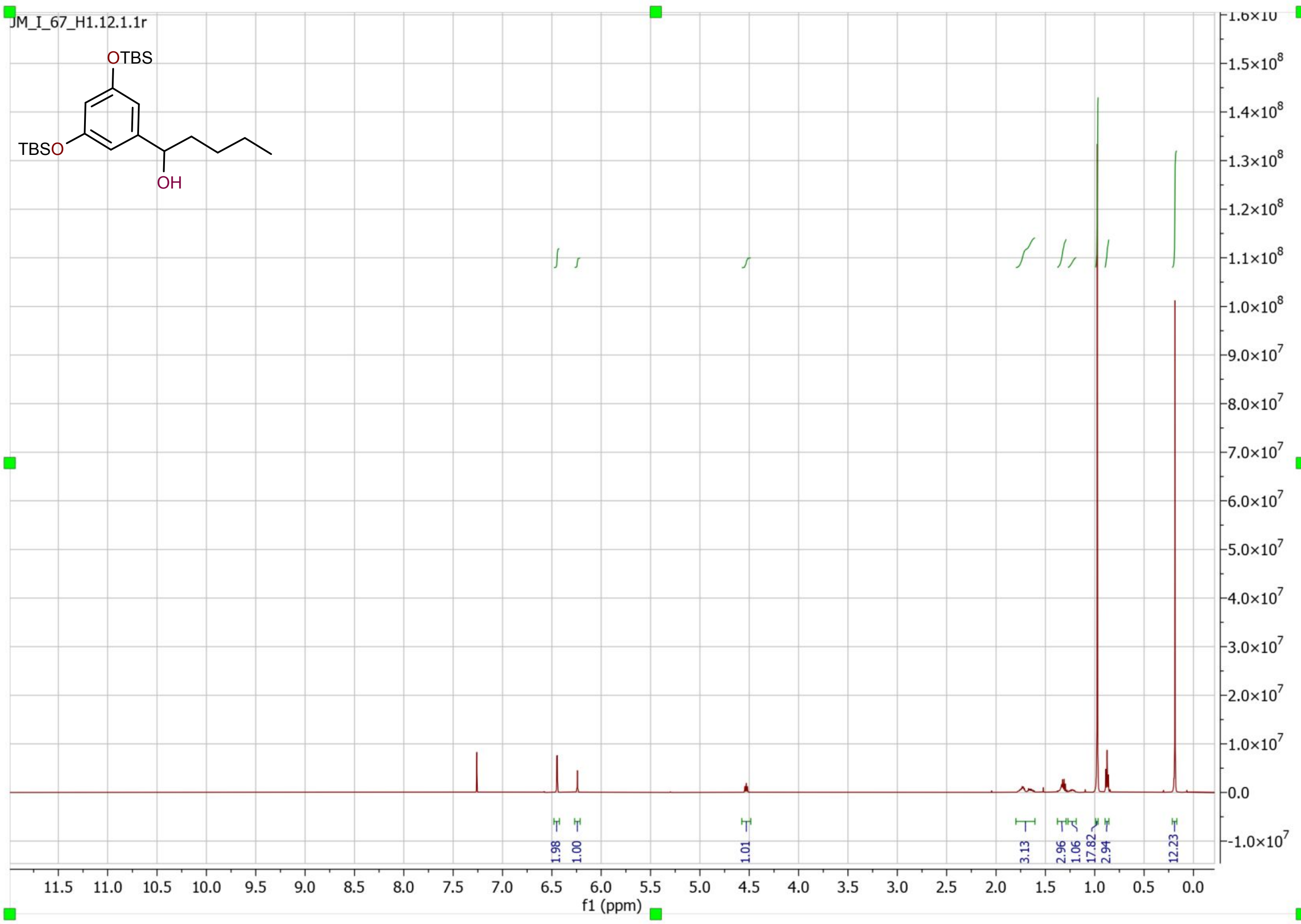
JM_I_67_H1.12.1.1r
OTBS
TBSO
OH
1.98
1.00
1.01
3.13
2.96
1.06
17.82
2.94
12.23
f1 (ppm)

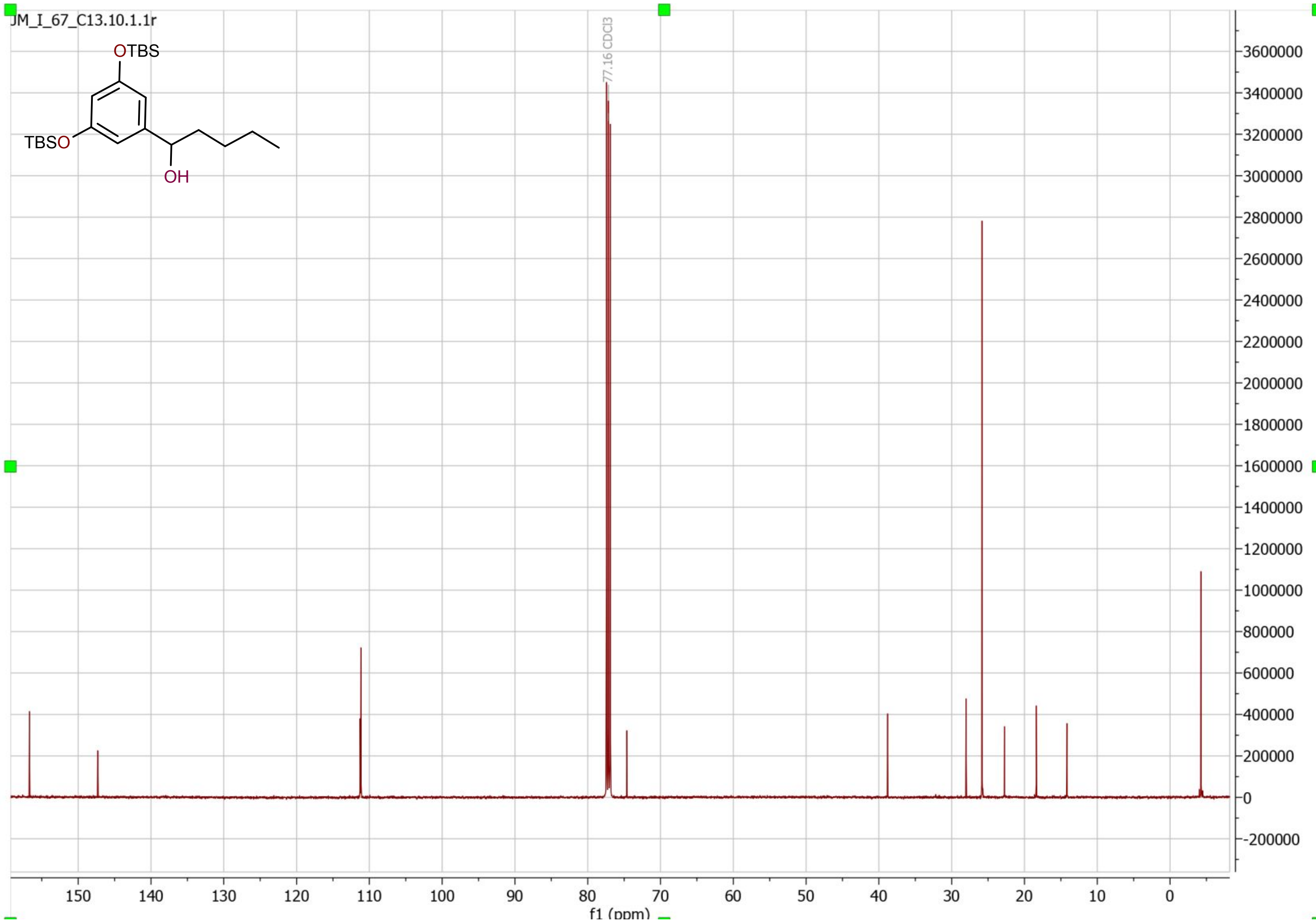
JM_I_67_C13.10.1.1r
OTBS
TBSO
OH
77.16 CDCl3
3600000
3400000
3200000
3000000
2800000
2600000
2400000
2200000
2000000
1800000
1600000
1400000
1200000
1000000
800000
600000
400000
200000
0
-200000
150
140
130
120
110
100
90
80
70
60
50
40
30
20
10
0
f1 (ppm)

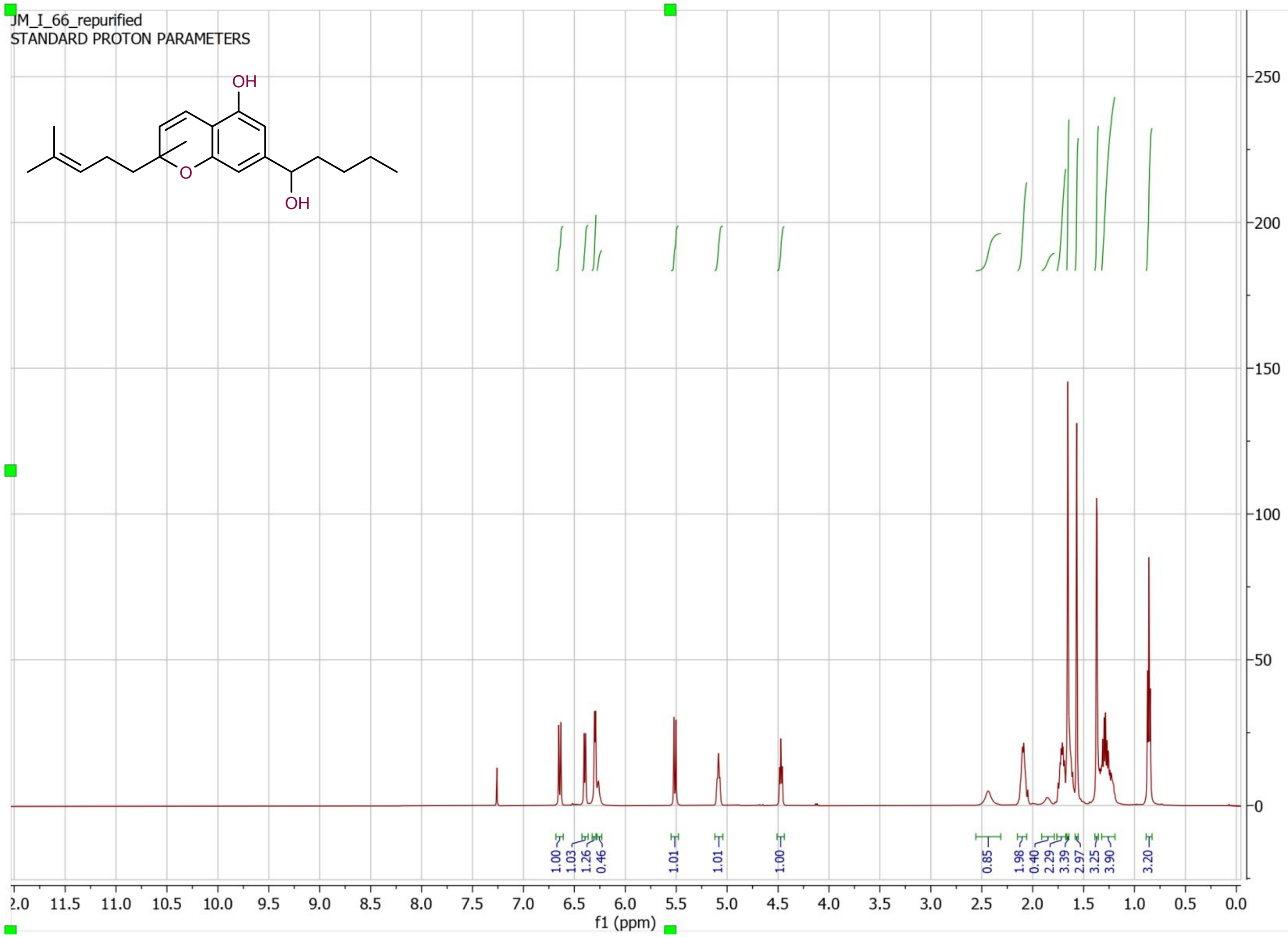
JM_I_66_repurified
STANDARD PROTON PARAMETERS
OH
O
OH
1.00
1.03
1.26
0.46
1.01
1.01
1.00
0.85
1.98
0.40
2.29
3.39
2.97
3.25
3.90
3.20
f1 (ppm)

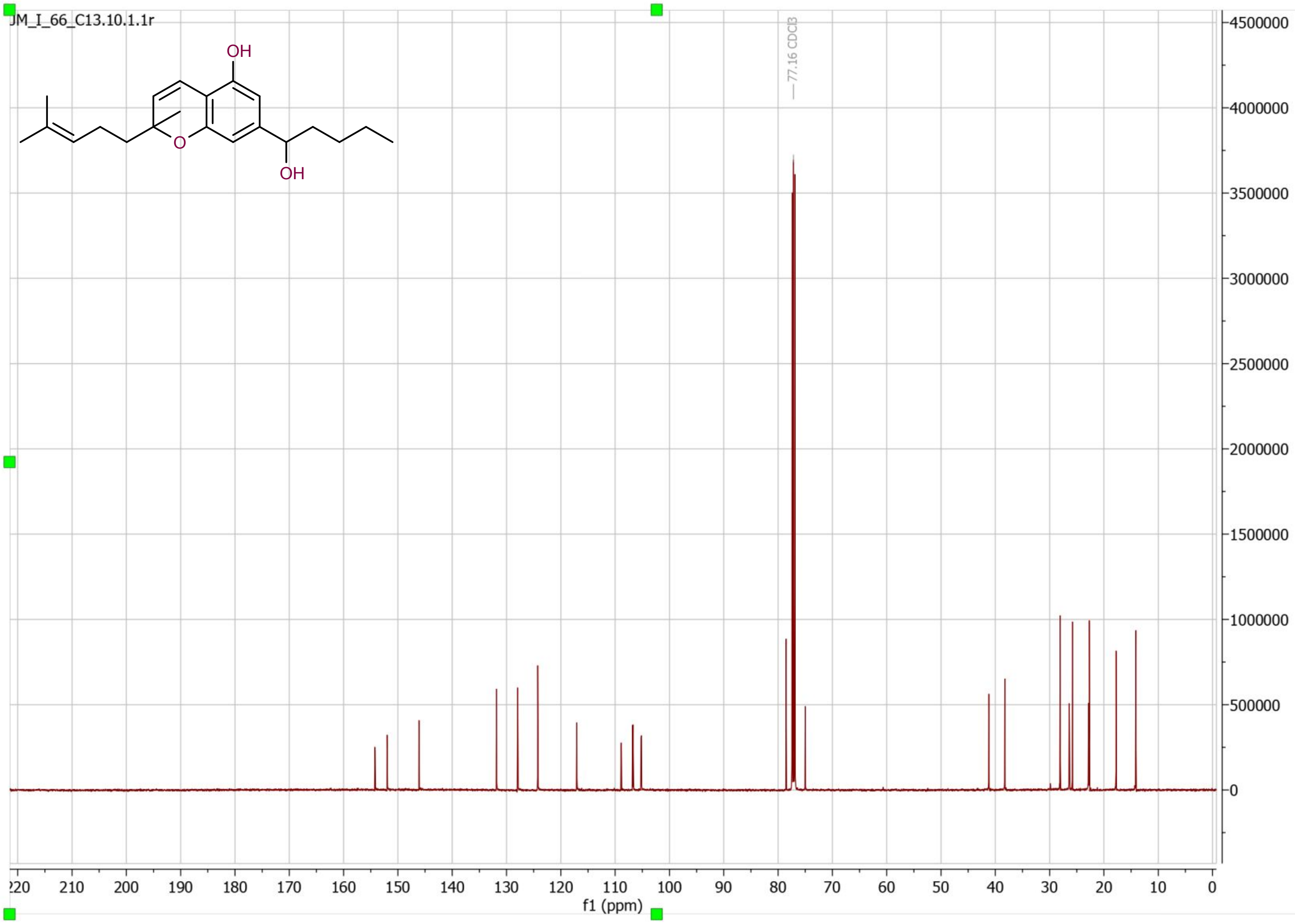
JM_I_66_C13.10.1.1r
OH
O
OH
77.16 CDCl3
4500000
4000000
3500000
3000000
2500000
2000000
1500000
1000000
500000
0
220
210
200
190
180
170
160
150
140
130
120
110
100
90
80
70
60
50
40
30
20
10
0
f1 (ppm)

1.4 Mass Analysis

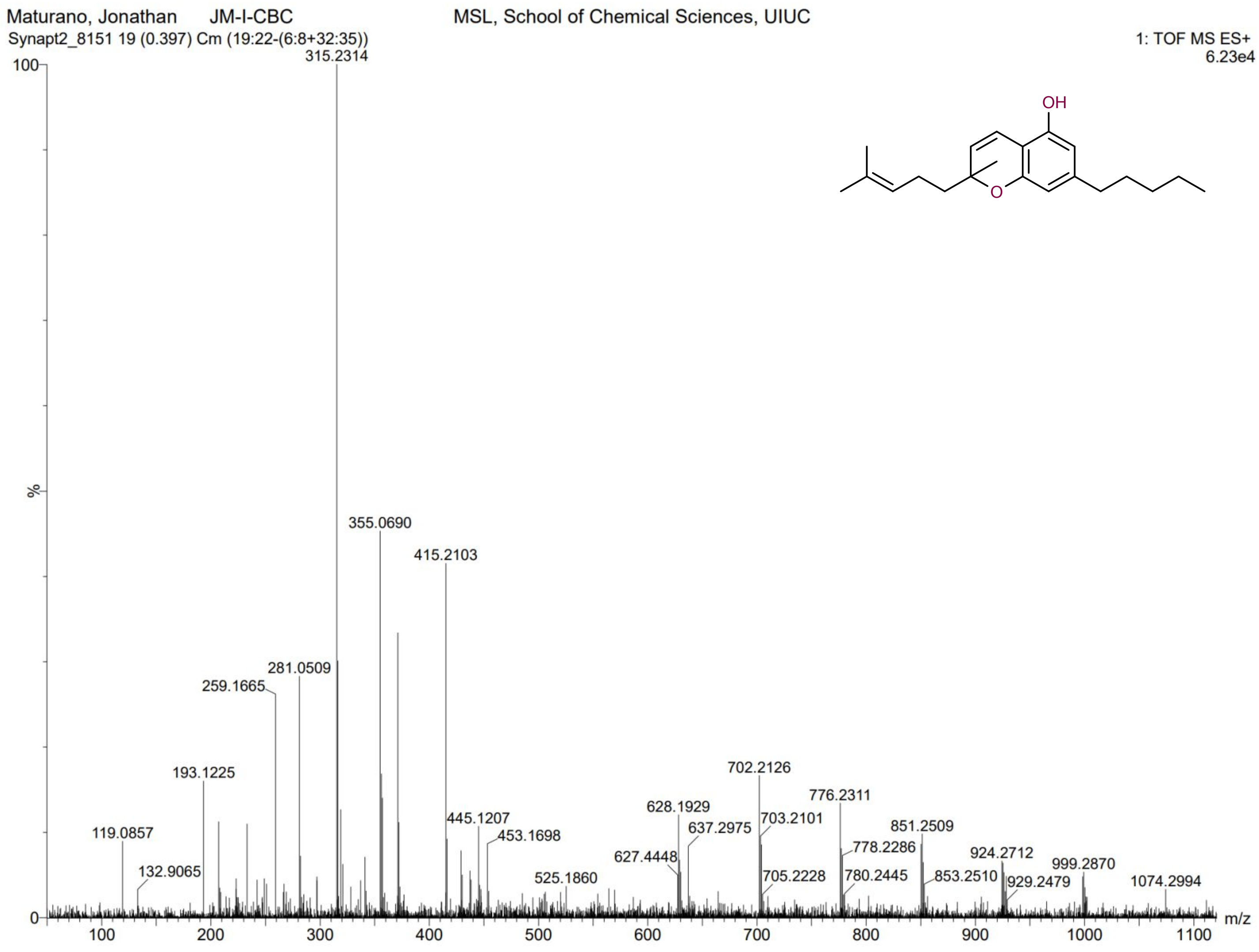

Elemental Composition Report

Single Mass Analysis

Tolerance = 5.0 PPM / DBE: min = -1.5, max = 200.0
Element prediction: Off
Number of isotope peaks used for i-FIT = 9

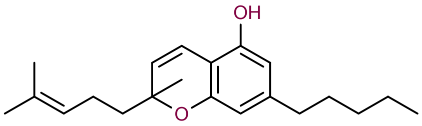

Monoisotopic Mass, Even Electron Ions
98 formula(e) evaluated with 2 results within limits (up to 50 closest results for each mass)
Elements Used:
C: 0-120 H: 0-180 O: 0-5 Na: 0-1 Si: 0-1

Maturano, Jonathan JM-I-CBC MSL, School of Chemical Sciences, UIUC
Synapt2_8151 19 (0.397) Cm (19:22-(6:8+32:35)) 1: TOF MS ES+
6.23e+004

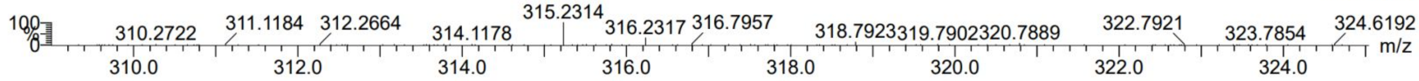

Minimum:				-1.5				
Maximum:		5.0	5.0	200.0				
Mass	Calc. Mass	mDa	PPM	DBE	i-FIT	Norm	Conf(%)	Formula
315.2314	315.2324	-1.0	-3.2	6.5	593.6	0.925	39.66	C21 H31 O2
	315.2300	1.4	4.4	3.5	593.2	0.505	60.34	C19 H32 O2 Na

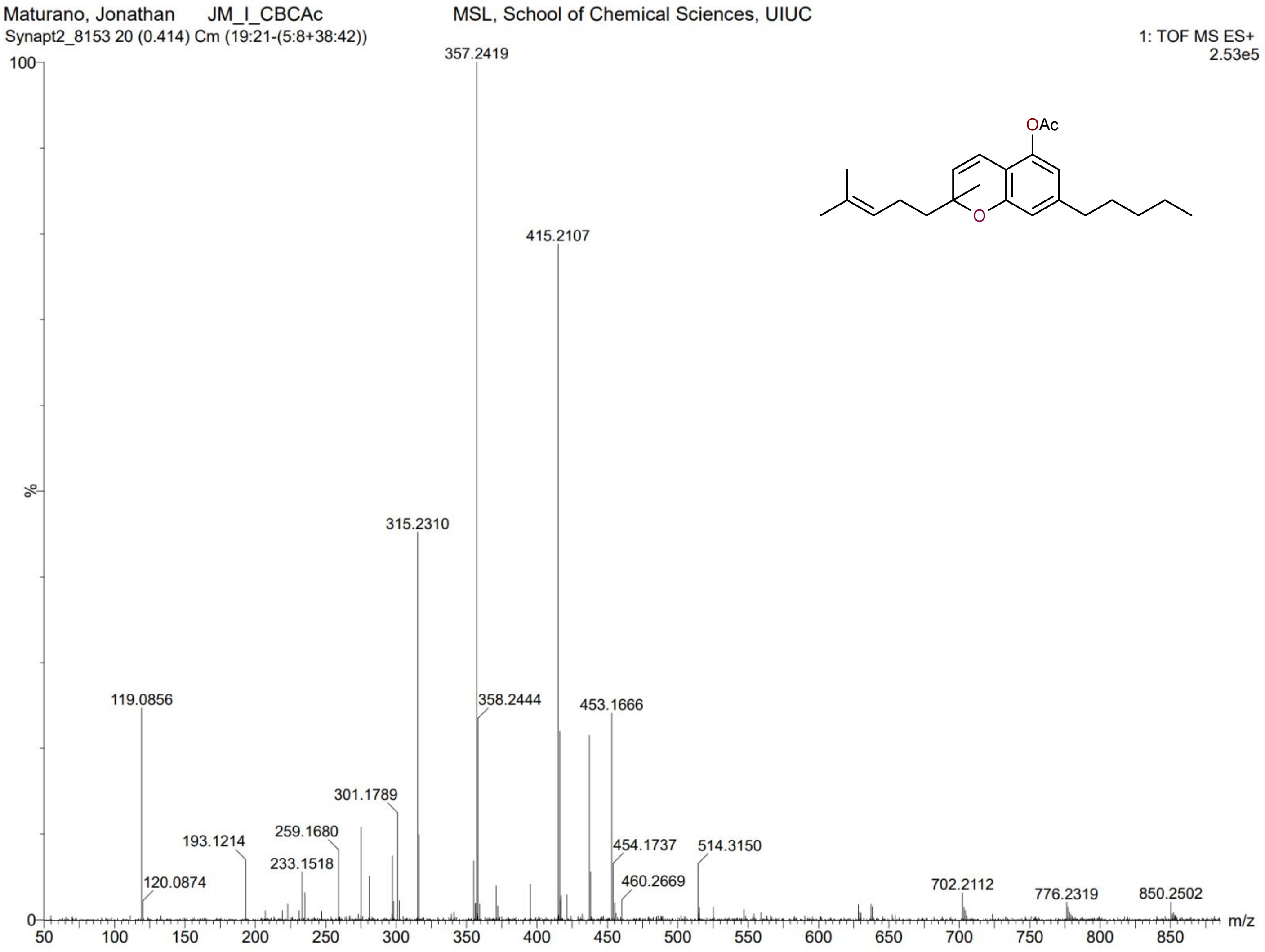

Maturano, Jonathan JM_I_CBCAc
MSL, School of Chemical Sciences, UIUC
Synapt2_8153 20 (0.414) Cm (19:21-(5:8+38:42))
1: TOF MS ES+
2.53e5
%
m/z
357.2419
415.2107
315.2310
119.0856
358.2444
453.1666
301.1789
193.1214
259.1680
233.1518
120.0874
454.1737
514.3150
460.2669
702.2112
776.2319
850.2502
OAc
O

Elemental Composition Report

Single Mass Analysis

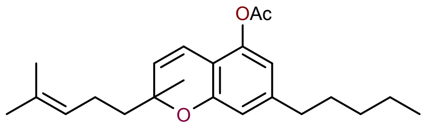

Tolerance = 5.0 PPM / DBE: min = -1.5, max = 200.0
Element prediction: Off
Number of isotope peaks used for i-FIT = 9

Monoisotopic Mass, Even Electron Ions
106 formula(e) evaluated with 2 results within limits (up to 50 closest results for each mass)
Elements Used:
C: 0-120 H: 0-180 O: 0-5 Na: 0-1 Si: 0-1
Maturano, Jonathan JM_I_CBCAc MSL, School of Chemical Sciences, UIUC
Synapt2_8153 20 (0.414) Cm (19:21-(5:8+38:42)) 1: TOF MS ES+ 2.53e+005

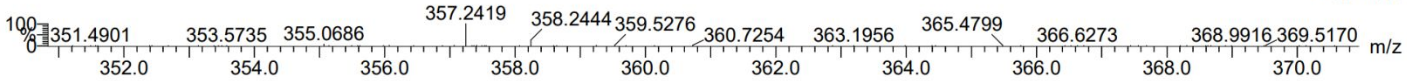

Minimum:				-1.5				
Maximum:		5.0	5.0	200.0				

Mass	Calc. Mass	mDa	PPM	DBE	i-FIT	Norm	Conf(%)	Formula
357.2419	357.2430	-1.1	-3.1	7.5	271.6	1.689	18.48	C23 H33 O3
	357.2406	1.3	3.6	4.5	270.1	0.204	81.52	C21 H34 O3 Na

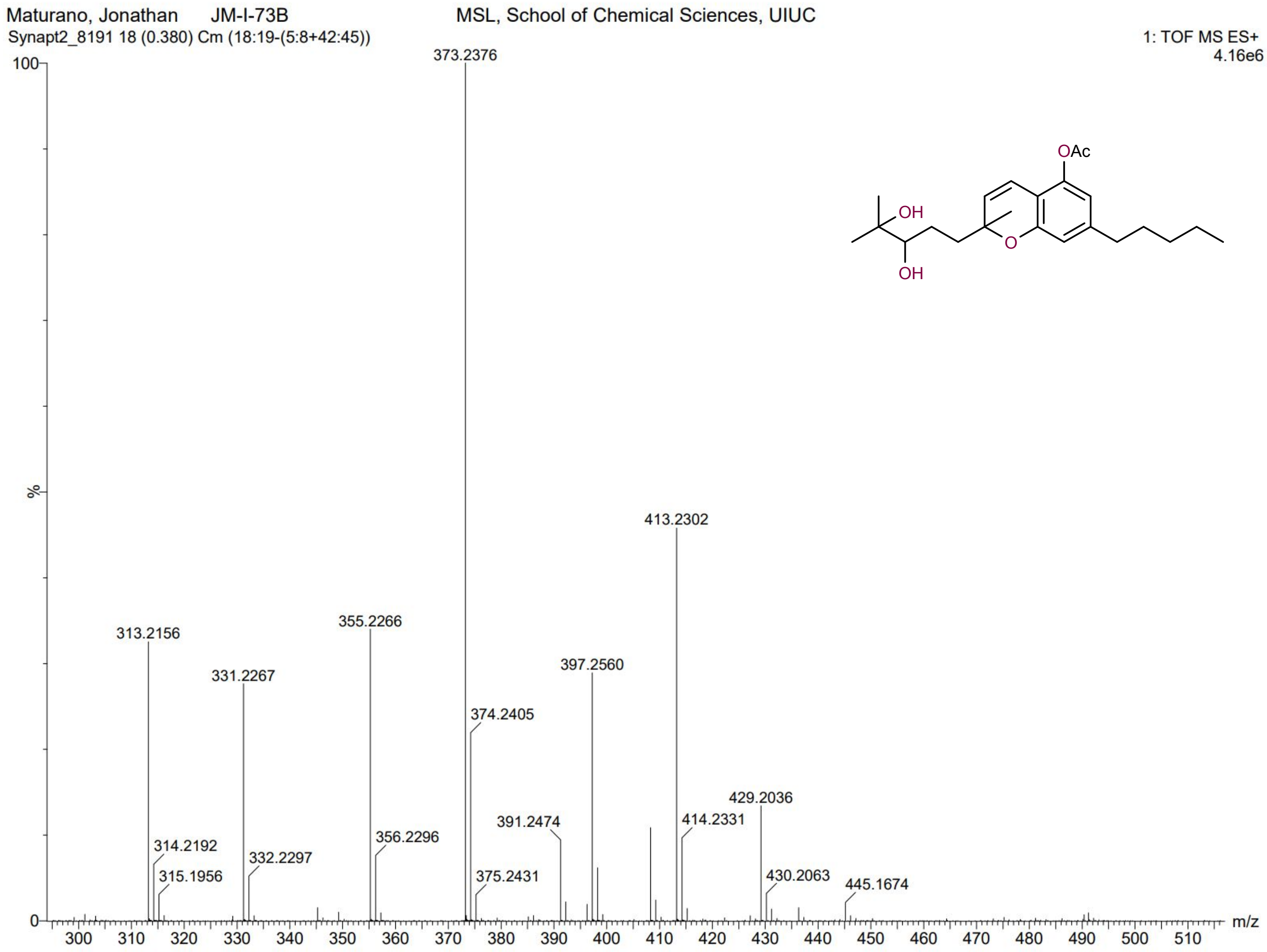

Maturano, Jonathan JM-I-73B
MSL, School of Chemical Sciences, UIUC
Synapt2_8191 18 (0.380) Cm (18:19-(5:8+42:45))
1: TOF MS ES+
4.16e6
OAc
OH
OH
O
373.2376
413.2302
313.2156
355.2266
331.2267
397.2560
374.2405
429.2036
391.2474
414.2331
356.2296
314.2192
332.2297
315.1956
375.2431
430.2063
445.1674
%
m/z

Elemental Composition Report

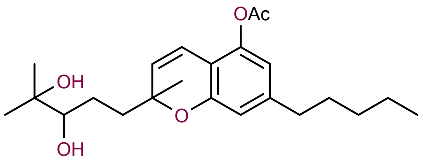

Single Mass Analysis

Tolerance = 5.0 PPM / DBE: min = -1.5, max = 200.0
Element prediction: Off
Number of isotope peaks used for i-FIT = 9

Monoisotopic Mass, Even Electron Ions
206 formula(e) evaluated with 1 results within limits (up to 50 closest results for each mass)
Elements Used:
C: 0-120 H: 0-180 N: 0-5 O: 0-6
Maturano, Jonathan JM-I-73B MSL, School of Chemical Sciences, UIUC
Synapt2_8191 18 (0.380) Cm (18:19-(5:8+42:45)) 1: TOF MS ES+
3.91e+005

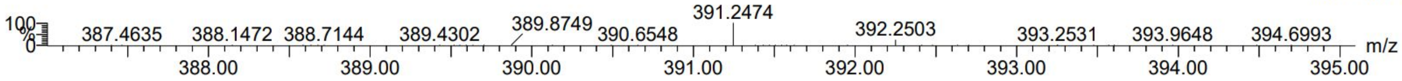

Minimum:				-1.5				
Maximum:		5.0	5.0	200.0				
Mass	Calc. Mass	mDa	PPM	DBE	i-FIT	Norm	Conf(%)	Formula
391.2474	391.2484	-1.0	-2.6	6.5	265.2	n/a	n/a	C23 H35 O5

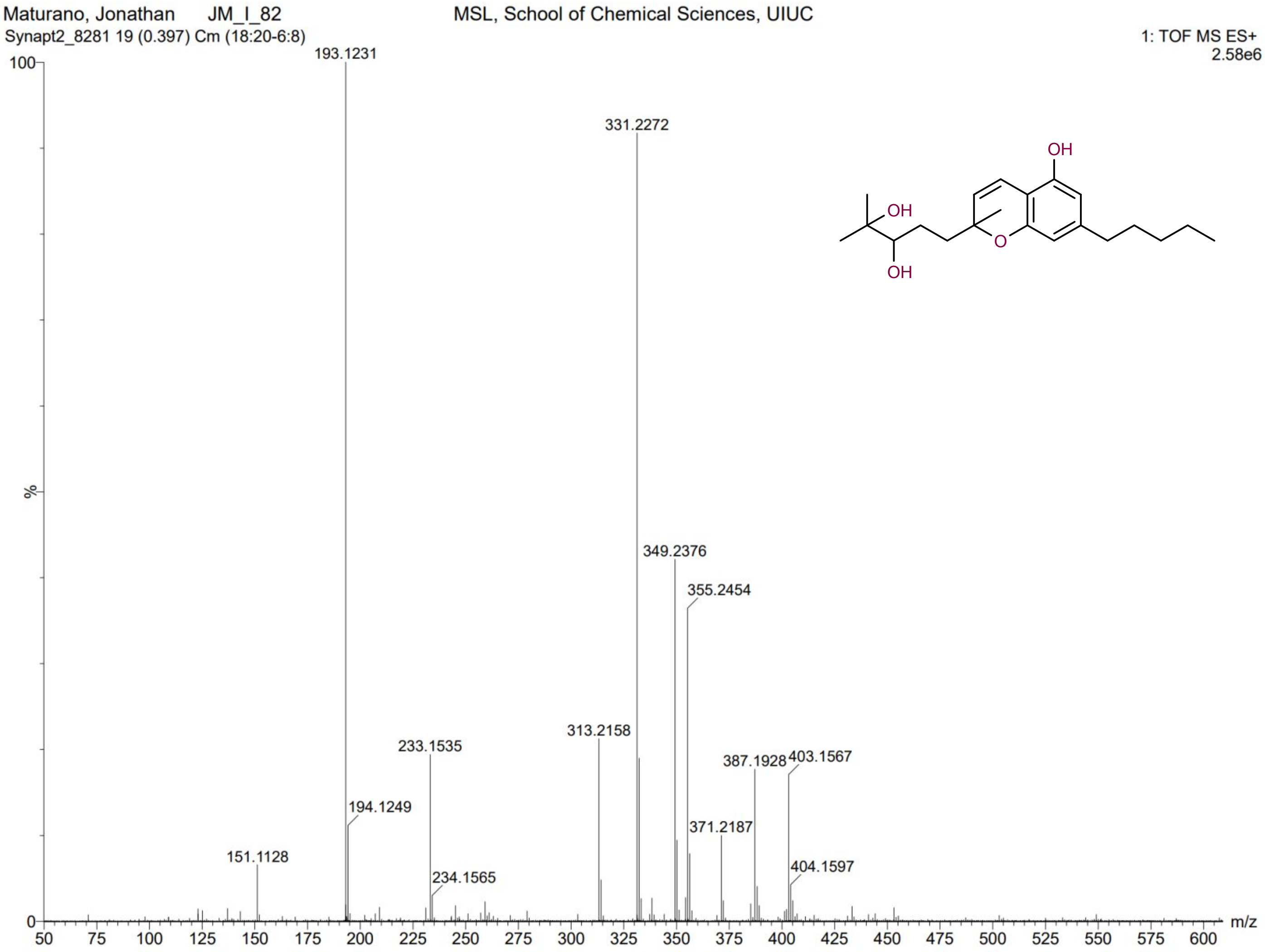

Maturano, Jonathan JM_I_82
MSL, School of Chemical Sciences, UIUC
Synapt2_8281 19 (0.397) Cm (18:20-6:8)
1: TOF MS ES+
2.58e6
193.1231
331.2272
349.2376
355.2454
313.2158
233.1535
387.1928
403.1567
194.1249
371.2187
151.1128
404.1597
234.1565
OH
OH
OH
O
%
m/z

Elemental Composition Report

Single Mass Analysis

Tolerance = 5.0 PPM / DBE: min = -1.5, max = 200.0
Element prediction: Off
Number of isotope peaks used for i-FIT = 9

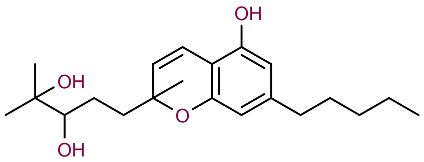

Monoisotopic Mass, Even Electron Ions
355 formula(e) evaluated with 3 results within limits (up to 50 closest results for each mass)
Elements Used:
C: 0-120 H: 0-180 N: 0-6 O: 0-5 Na: 0-1
Maturano, Jonathan JM_I_82 MSL, School of Chemical Sciences, UIUC
Synapt2_8281 19 (0.397) Cm (18:20-6:8)

1: TOF MS ES+
1.09e+006

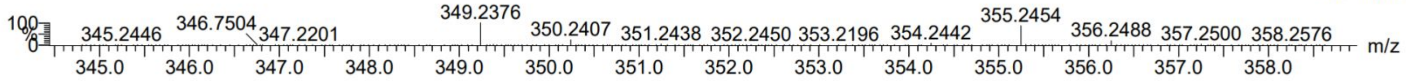

Minimum:				-1.5				
Maximum:		5.0	5.0	200.0				
Mass	Calc. Mass	mDa	PPM	DBE	i-FIT	Norm	Conf(%)	Formula
349.2376	349.2379	-0.3	-0.9	5.5	1804.4	0.859	42.34	C21 H33 O4
	349.2368	0.8	2.3	7.5	1804.7	1.173	30.94	C20 H30 N4 Na
	349.2392	-1.6	-4.6	10.5	1804.9	1.320	26.71	C22 H29 N4

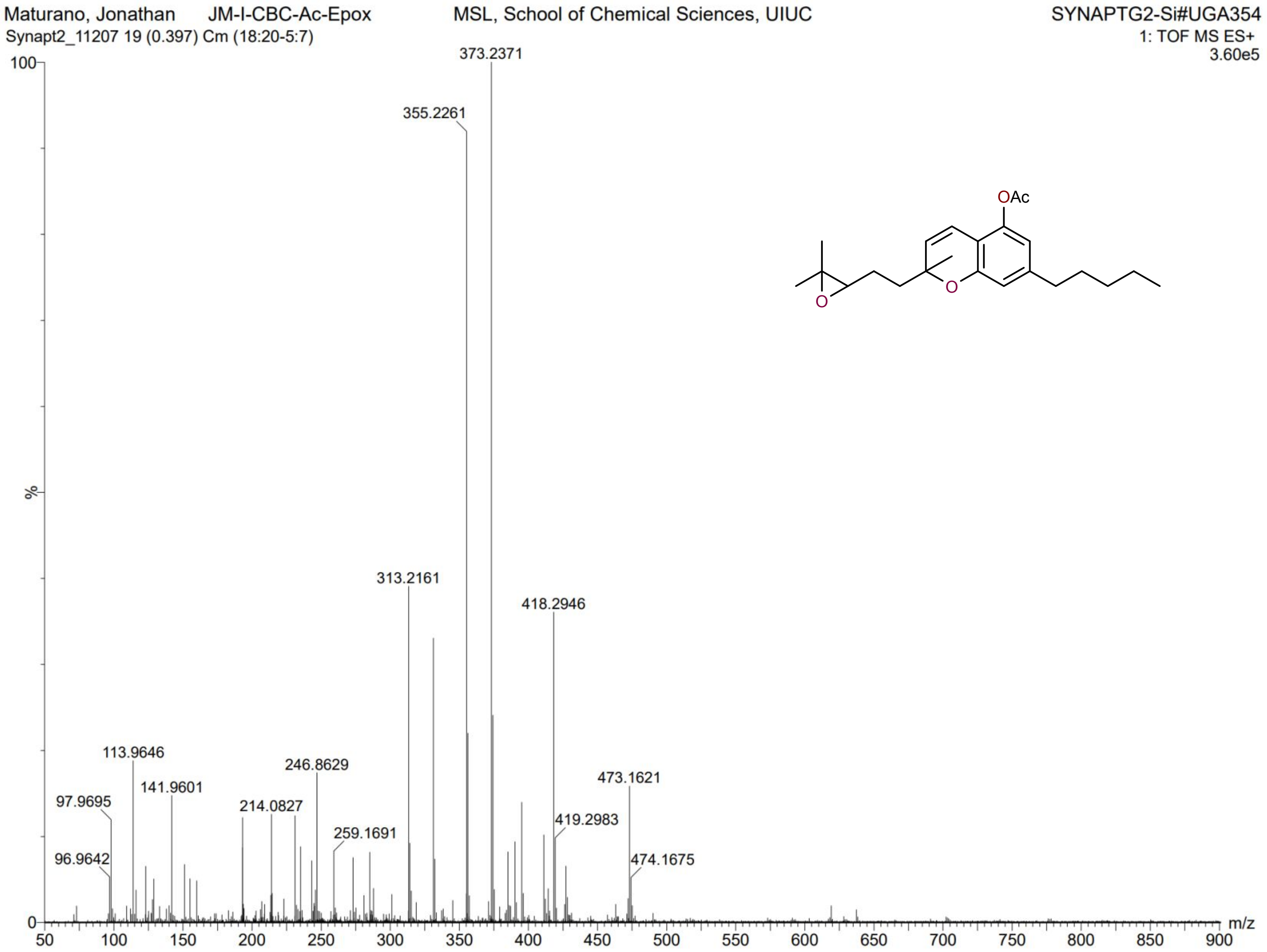

Maturano, Jonathan JM-I-CBC-Ac-Epox MSL, School of Chemical Sciences, UIUC SYNAPTG2-Si#UGA354
Synapt2_11207 19 (0.397) Cm (18:20-5:7)
1: TOF MS ES+
3.60e5
373.2371
355.2261
313.2161
418.2946
113.9646
246.8629
473.1621
141.9601
97.9695
214.0827
419.2983
259.1691
96.9642
474.1675
%
m/z
OAc
O
O

Elemental Composition Report

Single Mass Analysis

Tolerance = 5.0 PPM / DBE: min = -1.5, max = 200.0
Element prediction: Off
Number of isotope peaks used for i-FIT = 9

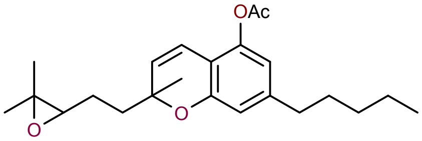

Monoisotopic Mass, Even Electron Ions
171 formula(e) evaluated with 1 results within limits (up to 50 closest results for each mass)
Elements Used:
C: 0-50 H: 0-100 N: 0-5 O: 0-5

Maturano, Jonathan JM-I-CBC-Ac-Epox MSL, School of Chemical Sciences, UIUC SYNAPTG2-Si#UGA354
Synapt2_11207 19 (0.397) Cm (18:20-5:7) 1: TOF MS ES+ 3.60e+005

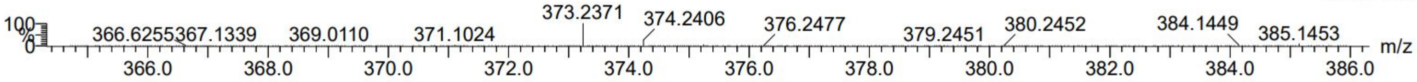

Minimum:				-1.5				
Maximum:		5.0	5.0	200.0				
Mass	Calc. Mass	mDa	PPM	DBE	i-FIT	Norm	Conf(%)	Formula
373.2371	373.2379	-0.8	-2.1	7.5	1542.2	n/a	n/a	C23 H33 O4

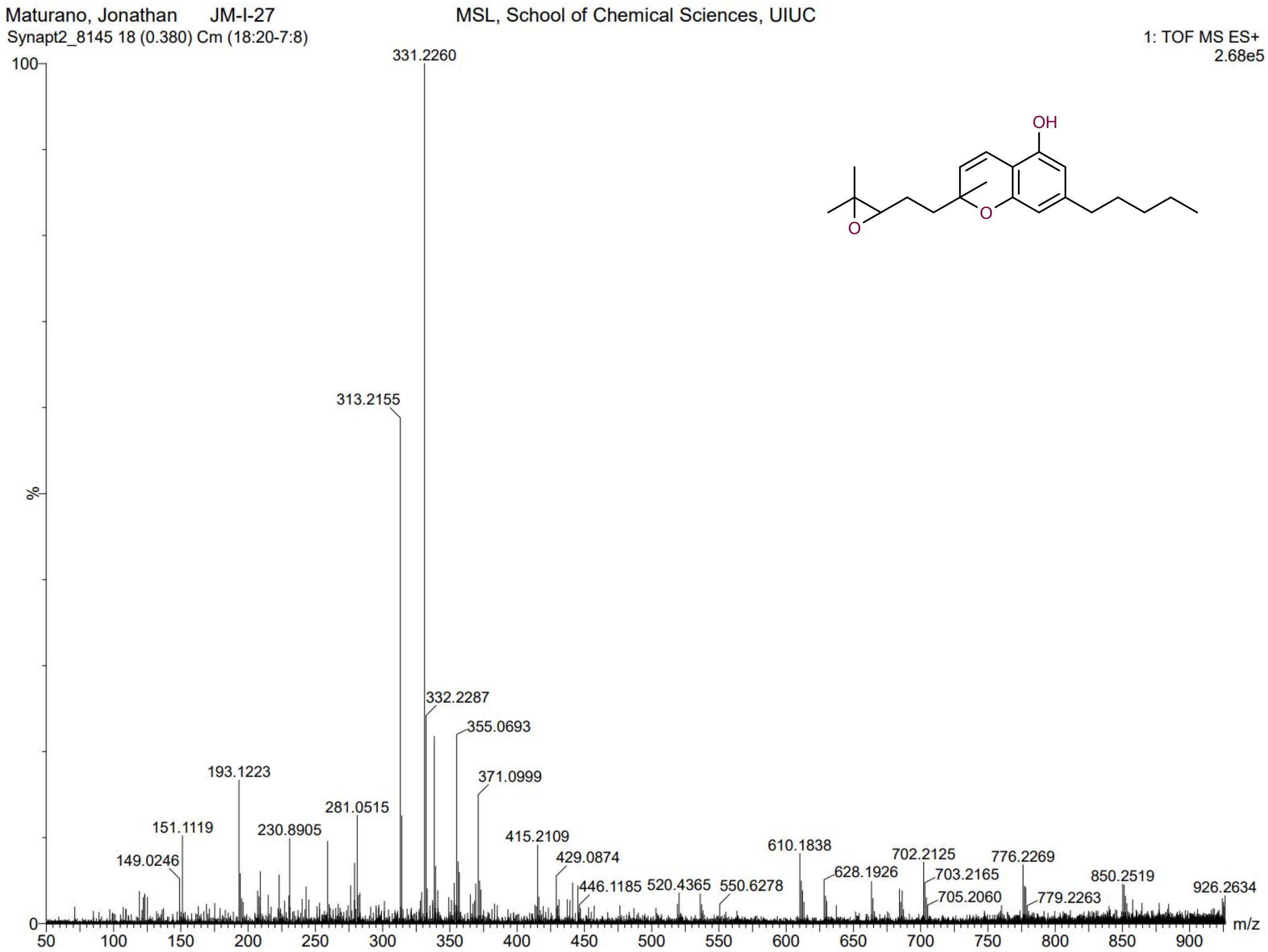

Maturano, Jonathan JM-I-27
MSL, School of Chemical Sciences, UIUC
Synapt2_8145 18 (0.380) Cm (18:20-7:8)
1: TOF MS ES+
2.68e5
OH
O
O
100
%
0
331.2260
313.2155
332.2287
355.0693
193.1223
371.0999
281.0515
151.1119
230.8905
415.2109
610.1838
149.0246
429.0874
702.2125
776.2269
628.1926
703.2165
850.2519
446.1185
520.4365
550.6278
926.2634
705.2060
779.2263
m/z
50
100
150
200
250
300
350
400
450
500
550
600
650
700
750
800
850
900

Elemental Composition Report

Single Mass Analysis

Tolerance = 5.0 PPM / DBE: min = -1.5, max = 200.0
Element prediction: Off
Number of isotope peaks used for i-FIT = 9

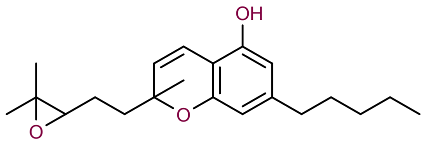

Monoisotopic Mass, Even Electron Ions
293 formula(e) evaluated with 2 results within limits (up to 50 closest results for each mass)
Elements Used:
C: 0-120 H: 0-180 N: 0-5 O: 0-5 Na: 0-1
Maturano, Jonathan JM-I-27 MSL, School of Chemical Sciences, UIUC
Synapt2_8145 18 (0.380) Cm (18:20-7:8) 1: TOF MS ES+ 2.68e+005

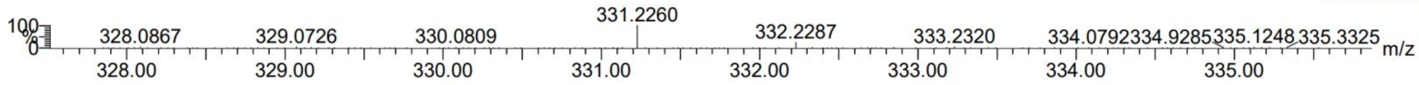

Minimum:				-1.5				
Maximum:		5.0	5.0	200.0				
Mass	Calc. Mass	mDa	PPM	DBE	i-FIT	Norm	Conf(%)	Formula
331.2260	331.2249	1.1	3.3	3.5	716.9	1.467	23.05	C19 H32 O3 Na
	331.2273	-1.3	-3.9	6.5	715.7	0.262	76.95	C21 H31 O3

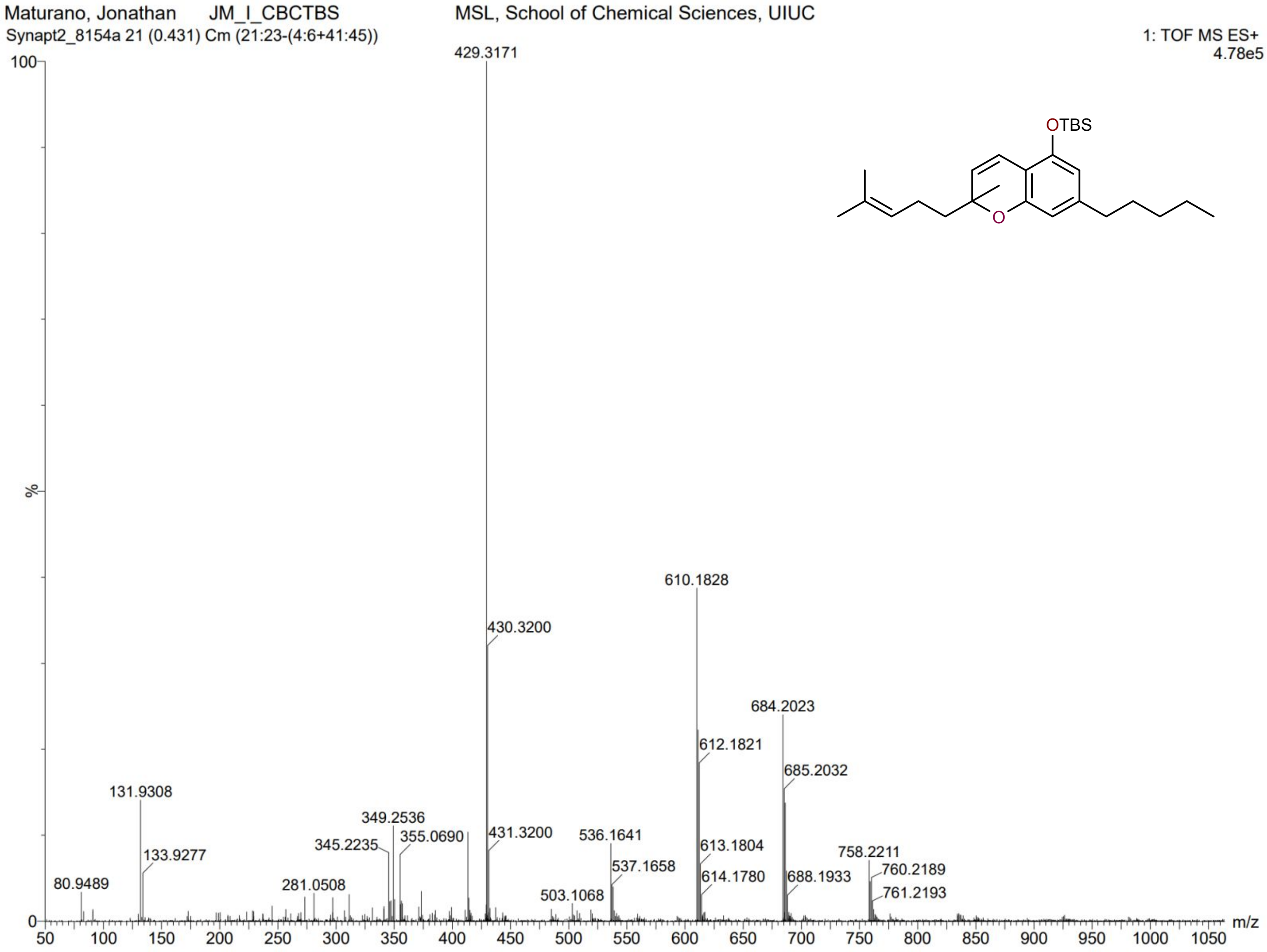
Maturano, Jonathan JM_I_CBCTBS
MSL, School of Chemical Sciences, UIUC
Synapt2_8154a 21 (0.431) Cm (21:23-(4:6+41:45))
1: TOF MS ES+
4.78e5
429.3171
430.3200
610.1828
612.1821
684.2023
685.2032
131.9308
133.9277
80.9489
349.2536
345.2235
355.0690
431.3200
536.1641
537.1658
503.1068
281.0508
613.1804
614.1780
688.1933
758.2211
760.2189
761.2193
%
m/z
OTBS
O

Elemental Composition Report

Single Mass Analysis

Tolerance = 5.0 PPM / DBE: min = -1.5, max = 200.0
Element prediction: Off
Number of isotope peaks used for i-FIT = 9

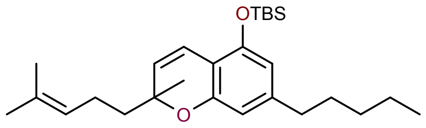

Monoisotopic Mass, Even Electron Ions
378 formula(e) evaluated with 3 results within limits (up to 50 closest results for each mass)
Elements Used:
C: 0-120 H: 0-180 N: 0-2 O: 0-5 Na: 0-1 Si: 0-1

Maturano, Jonathan JM_I_CBCTBS MSL, School of Chemical Sciences, UIUC
Synapt2_8154a 21 (0.431) Cm (21:23-(4:6+41:45))
1: TOF MS ES+
4.78e+005

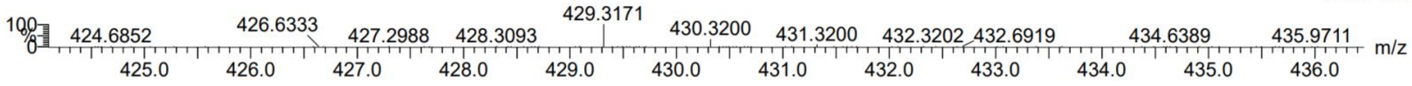

Minimum:				-1.5				
Maximum:		5.0	5.0	200.0				
Mass	Calc. Mass	mDa	PPM	DBE	i-FIT	Norm	Conf(%)	Formula
429.3171	429.3165	0.6	1.4	3.5	563.2	0.010	98.99	C25 H46 O2 Na Si
	429.3157	1.4	3.3	11.5	570.2	7.005	0.09	C31 H41 O
	429.3189	-1.8	-4.2	6.5	567.8	4.687	0.92	C27 H45 O2 Si

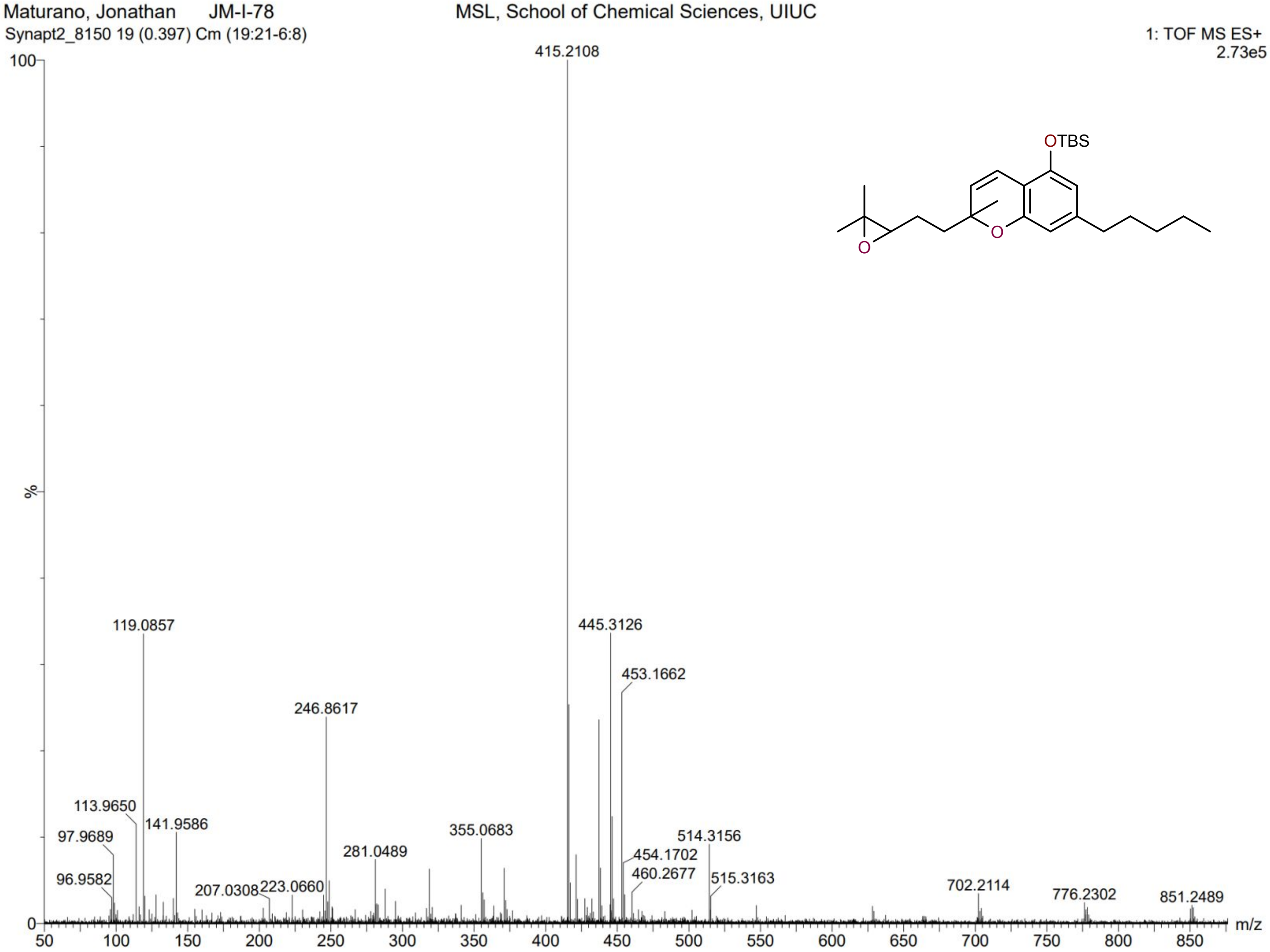

Maturano, Jonathan JM-I-78
MSL, School of Chemical Sciences, UIUC
Synapt2_8150 19 (0.397) Cm (19:21-6:8)
1: TOF MS ES+
2.73e5
415.2108
OTBS
O
O
119.0857
445.3126
453.1662
246.8617
113.9650
141.9586
97.9689
355.0683
514.3156
281.0489
454.1702
460.2677
96.9582
207.0308
223.0660
515.3163
702.2114
776.2302
851.2489
100
%
0
m/z
50
100
150
200
250
300
350
400
450
500
550
600
650
700
750
800
850

Elemental Composition Report

Single Mass Analysis

Tolerance = 5.0 PPM / DBE: min = -1.5, max = 200.0
Element prediction: Off
Number of isotope peaks used for i-FIT = 9

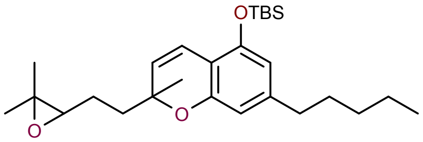

Monoisotopic Mass, Even Electron Ions
131 formula(e) evaluated with 3 results within limits (up to 50 closest results for each mass)
Elements Used:
C: 0-120 H: 0-180 O: 0-5 Na: 0-1 Si: 0-1

Maturano, Jonathan JM-I-78 MSL, School of Chemical Sciences, UIUC
Synapt2_8150 19 (0.397) Cm (19:21-6:8) 1: TOF MS ES+
9.17e+004

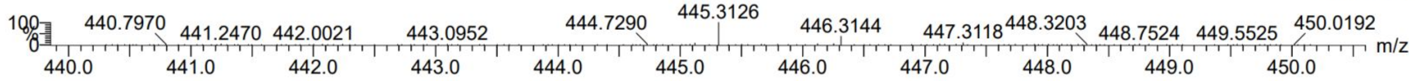

Minimum:				-1.5				
Maximum:		5.0	5.0	200.0				

Mass	Calc. Mass	mDa	PPM	DBE	i-FIT	Norm	Conf(%)	Formula
445.3126	445.3114	1.2	2.7	3.5	791.7	0.597	55.05	C25 H46 O3 Na Si
	445.3138	-1.2	-2.7	6.5	792.2	1.103	33.20	C27 H45 O3 Si
	445.3107	1.9	4.3	11.5	793.3	2.141	11.76	C31 H41 O2

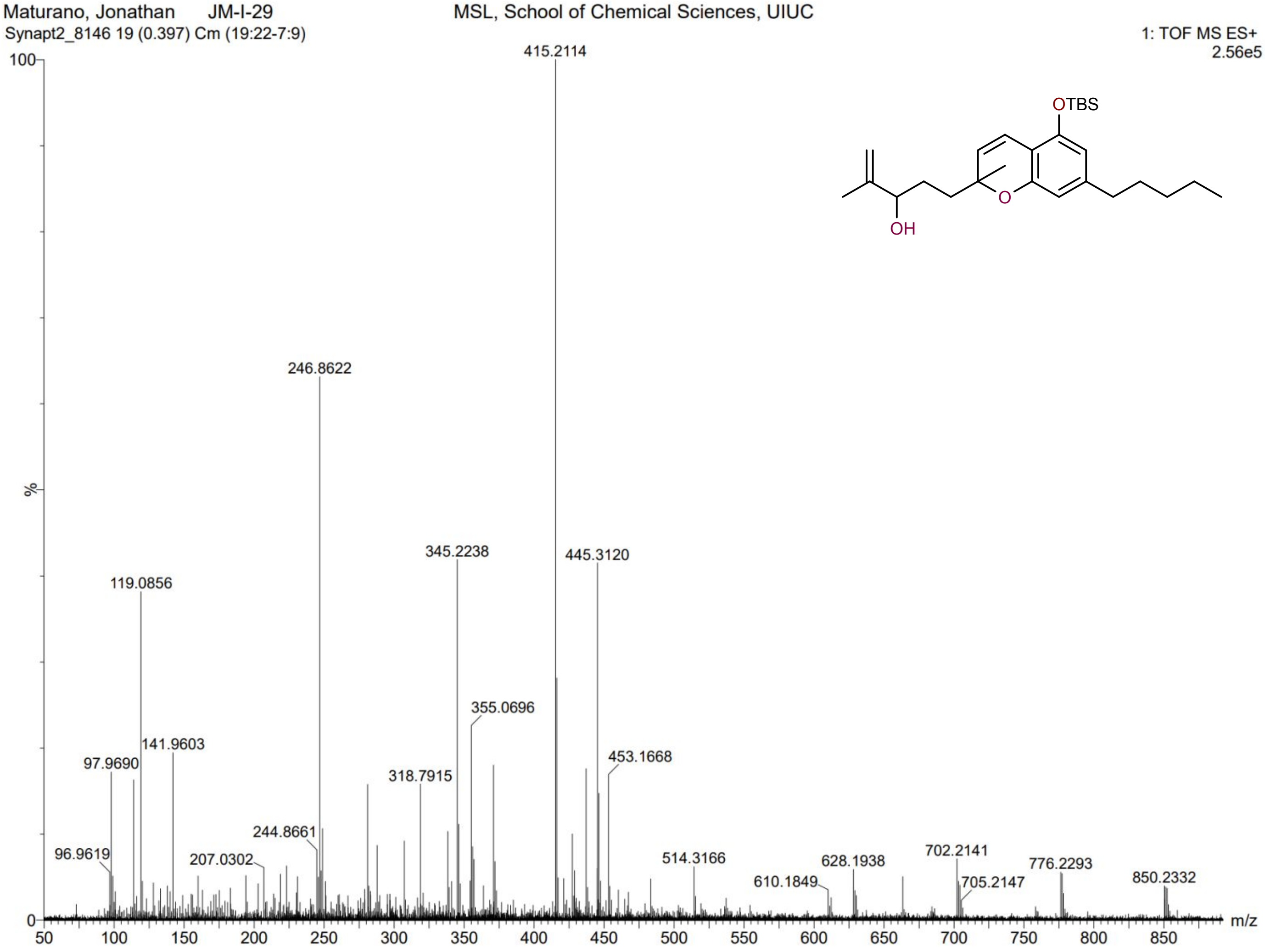
Maturano, Jonathan JM-I-29
MSL, School of Chemical Sciences, UIUC
Synapt2_8146 19 (0.397) Cm (19:22-7:9)
1: TOF MS ES+
2.56e5
415.2114
246.8622
345.2238
445.3120
119.0856
355.0696
141.9603
97.9690
453.1668
318.7915
244.8661
96.9619
207.0302
514.3166
628.1938
610.1849
702.2141
705.2147
776.2293
850.2332
%
m/z
OTBS
O
OH

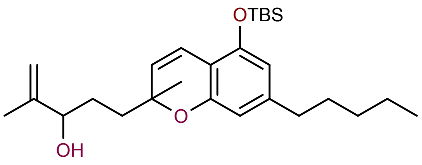
OTBS
O
OH

Elemental Composition Report

Page 1

Single Mass Analysis

Tolerance = 5.0 PPM / DBE: min = -1.5, max = 200.0
Element prediction: Off
Number of isotope peaks used for i-FIT = 9

Monoisotopic Mass, Even Electron Ions
131 formula(e) evaluated with 3 results within limits (up to 50 closest results for each mass)
Elements Used:
C: 0-120 H: 0-180 O: 0-5 Na: 0-1 Si: 0-1

Maturano, Jonathan JM-I-29 MSL, School of Chemical Sciences, UIUC
Synapt2_8146 19 (0.397) Cm (19:22-7:9) 1: TOF MS ES+
1.06e+005

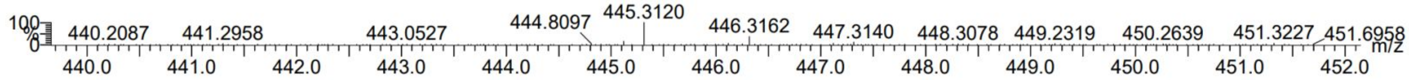

Minimum:				-1.5				
Maximum:		5.0	5.0	200.0				
Mass	Calc. Mass	mDa	PPM	DBE	i-FIT	Norm	Conf(%)	Formula
445.3120	445.3114	0.6	1.3	3.5	1327.7	0.770	46.30	C25 H46 O3 Na Si
	445.3107	1.3	2.9	11.5	1332.3	5.378	0.46	C31 H41 O2
	445.3138	-1.8	-4.0	6.5	1327.5	0.630	53.24	C27 H45 O3 Si

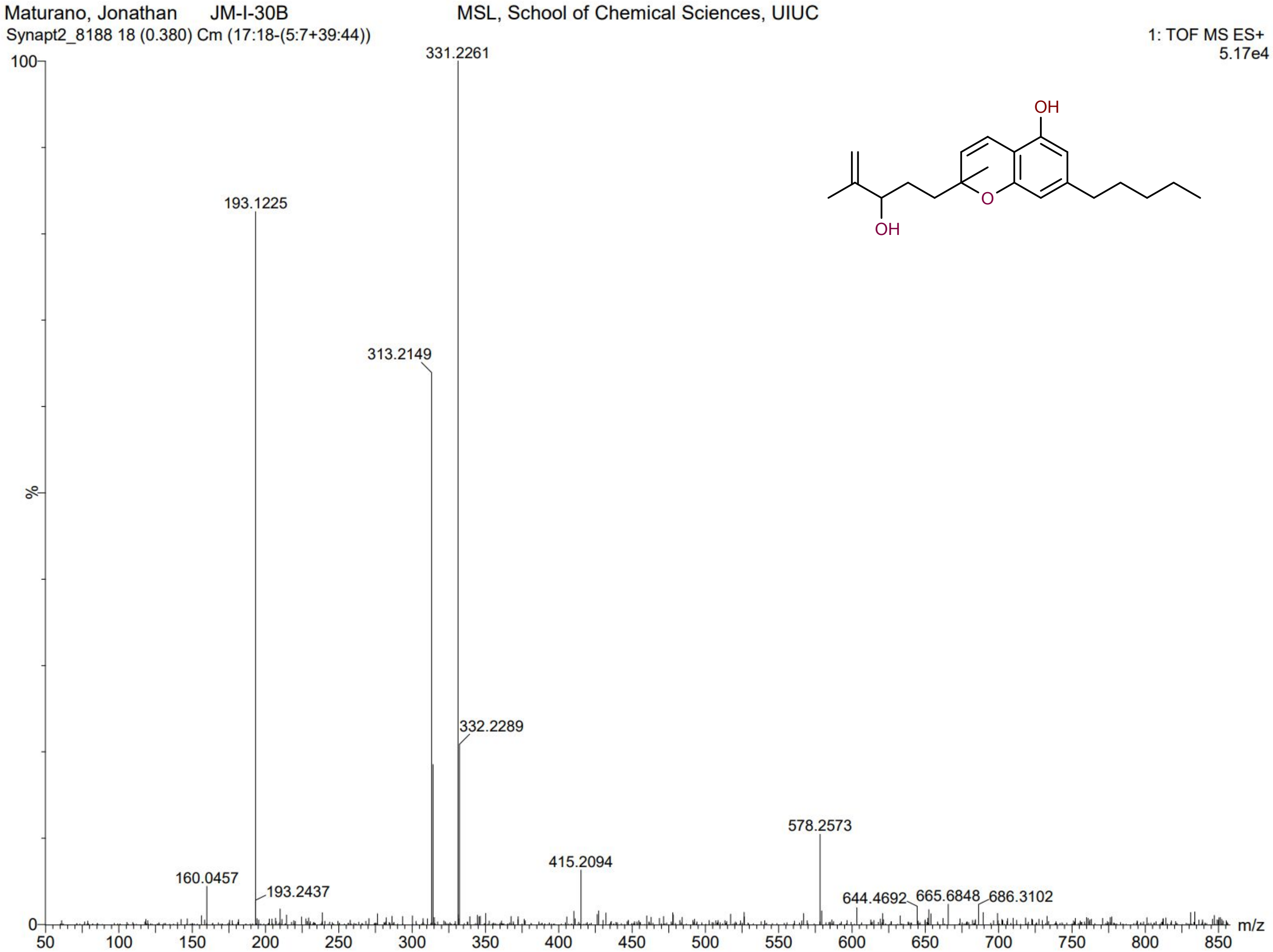
Maturano, Jonathan JM-I-30B
MSL, School of Chemical Sciences, UIUC
Synapt2_8188 18 (0.380) Cm (17:18-(5:7+39:44))
1: TOF MS ES+
5.17e4
331.2261
193.1225
313.2149
332.2289
578.2573
415.2094
160.0457
193.2437
644.4692
665.6848
686.3102
OH
O
OH
%
m/z

Elemental Composition Report

Single Mass Analysis

Tolerance = 5.0 PPM / DBE: min = -1.5, max = 200.0
Element prediction: Off
Number of isotope peaks used for i-FIT = 9

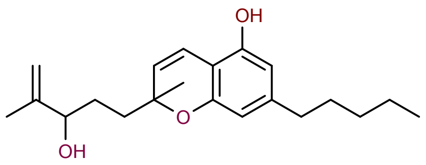

Monoisotopic Mass, Even Electron Ions
176 formula(e) evaluated with 1 results within limits (up to 50 closest results for each mass)
Elements Used:
C: 0-120 H: 0-180 N: 0-5 O: 0-6

Maturano, Jonathan JM-I-30B MSL, School of Chemical Sciences, UIUC
Synapt2_8188 18 (0.380) Cm (17:18-(5:7+39:44)) 1: TOF MS ES+ 5.17e+004

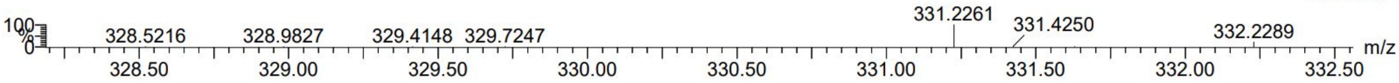

Minimum:				-1.5				
Maximum:		5.0	5.0	200.0				
Mass	Calc. Mass	mDa	PPM	DBE	i-FIT	Norm	Conf(%)	Formula
331.2261	331.2273	-1.2	-3.6	6.5	90.3	n/a	n/a	C21 H31 O3

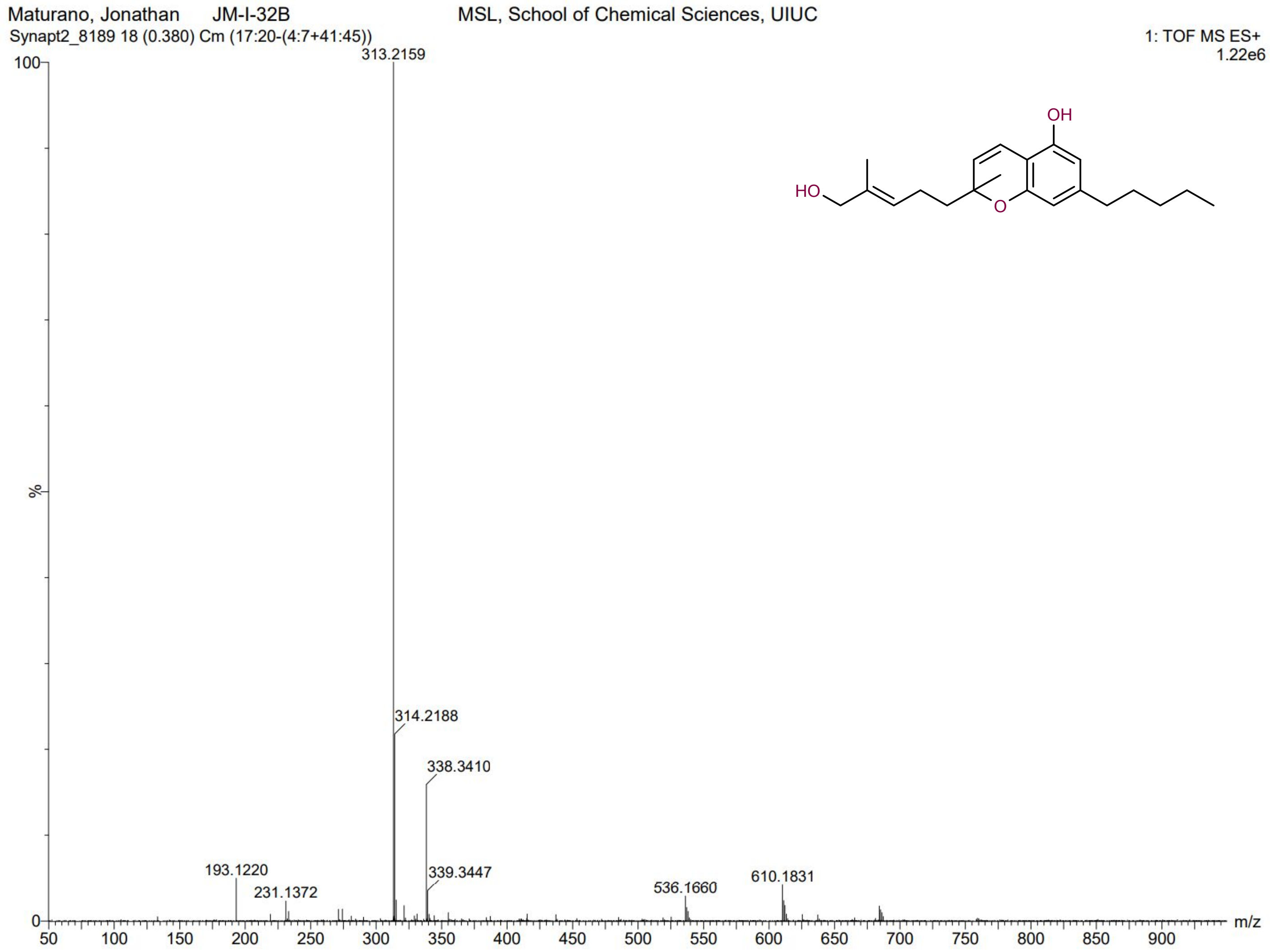

Maturano, Jonathan JM-I-32B
MSL, School of Chemical Sciences, UIUC
Synapt2_8189 18 (0.380) Cm (17:20-(4:7+41:45))
1: TOF MS ES+
1.22e6
313.2159
314.2188
338.3410
339.3447
193.1220
231.1372
536.1660
610.1831
%
m/z
OH
HO
O

Elemental Composition Report

Single Mass Analysis

Tolerance = 5.0 PPM / DBE: min = -1.5, max = 200.0
Element prediction: Off
Number of isotope peaks used for i-FIT = 9

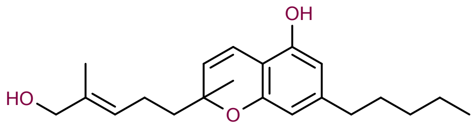

Monoisotopic Mass, Even Electron Ions
142 formula(e) evaluated with 2 results within limits (up to 50 closest results for each mass)
Elements Used:
C: 0-120 H: 0-180 N: 0-2 O: 0-5 Na: 0-1

Maturano, Jonathan JM_I_32 MSL, School of Chemical Sciences, UIUC
Synapt2_8152 19 (0.397) Cm (18:20-(5:9+41:46)) 1: TOF MS ES+ 1.16e+005

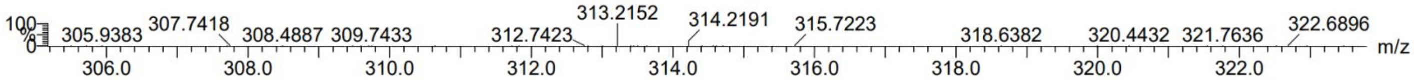

Mass	Calc. Mass	mDa	PPM	DBE	i-FIT	Norm	Conf(%)	Formula
Minimum:				-1.5				
Maximum:		5.0	5.0	200.0				
313.2152	313.2143	0.9	2.9	4.5	195.7	0.711	49.12	C19 H30 O2 Na
	313.2168	-1.6	-5.1	7.5	195.7	0.676	50.88	C21 H29 O2

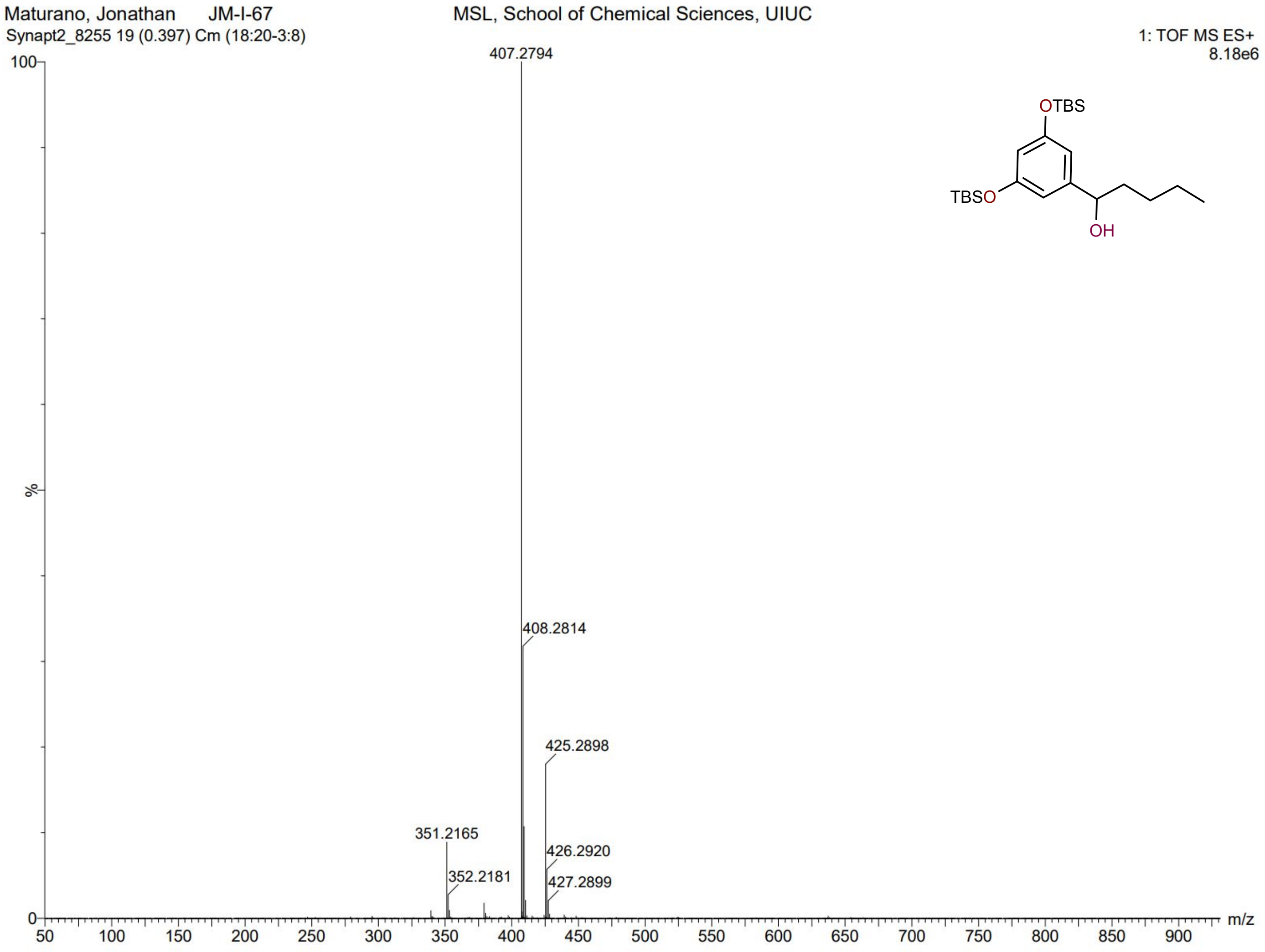

Maturano, Jonathan JM-I-67
MSL, School of Chemical Sciences, UIUC
Synapt2_8255 19 (0.397) Cm (18:20-3:8)
1: TOF MS ES+
8.18e6
407.2794
408.2814
425.2898
351.2165
426.2920
352.2181
427.2899
OTBS
TBSO
OH
%
m/z

Elemental Composition Report

Single Mass Analysis

Tolerance = 5.0 PPM / DBE: min = -1.5, max = 200.0
Element prediction: Off
Number of isotope peaks used for i-FIT = 9

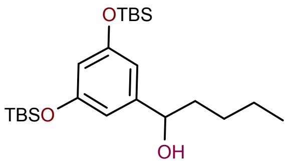

Monoisotopic Mass, Even Electron Ions
138 formula(e) evaluated with 2 results within limits (up to 50 closest results for each mass)
Elements Used:
C: 0-120 H: 0-180 O: 0-6 Na: 0-1 Si: 1-2

Maturano, Jonathan JM-I-67 MSL, School of Chemical Sciences, UIUC
Synapt2_8255 19 (0.397) Cm (18:20-3:8) 1: TOF MS ES+ 1.47e+006

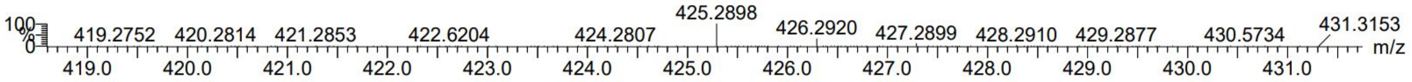

Mass	Calc. Mass	mDa	PPM	DBE	i-FIT	Norm	Conf(%)	Formula
Minimum:				-1.5				
Maximum:		5.0	5.0	200.0				
425.2898	425.2907	-0.9	-2.1	3.5	851.8	1.267	28.16	C23 H45 O3 Si2
	425.2883	1.5	3.5	0.5	850.9	0.331	71.84	C21 H46 O3 Na Si2

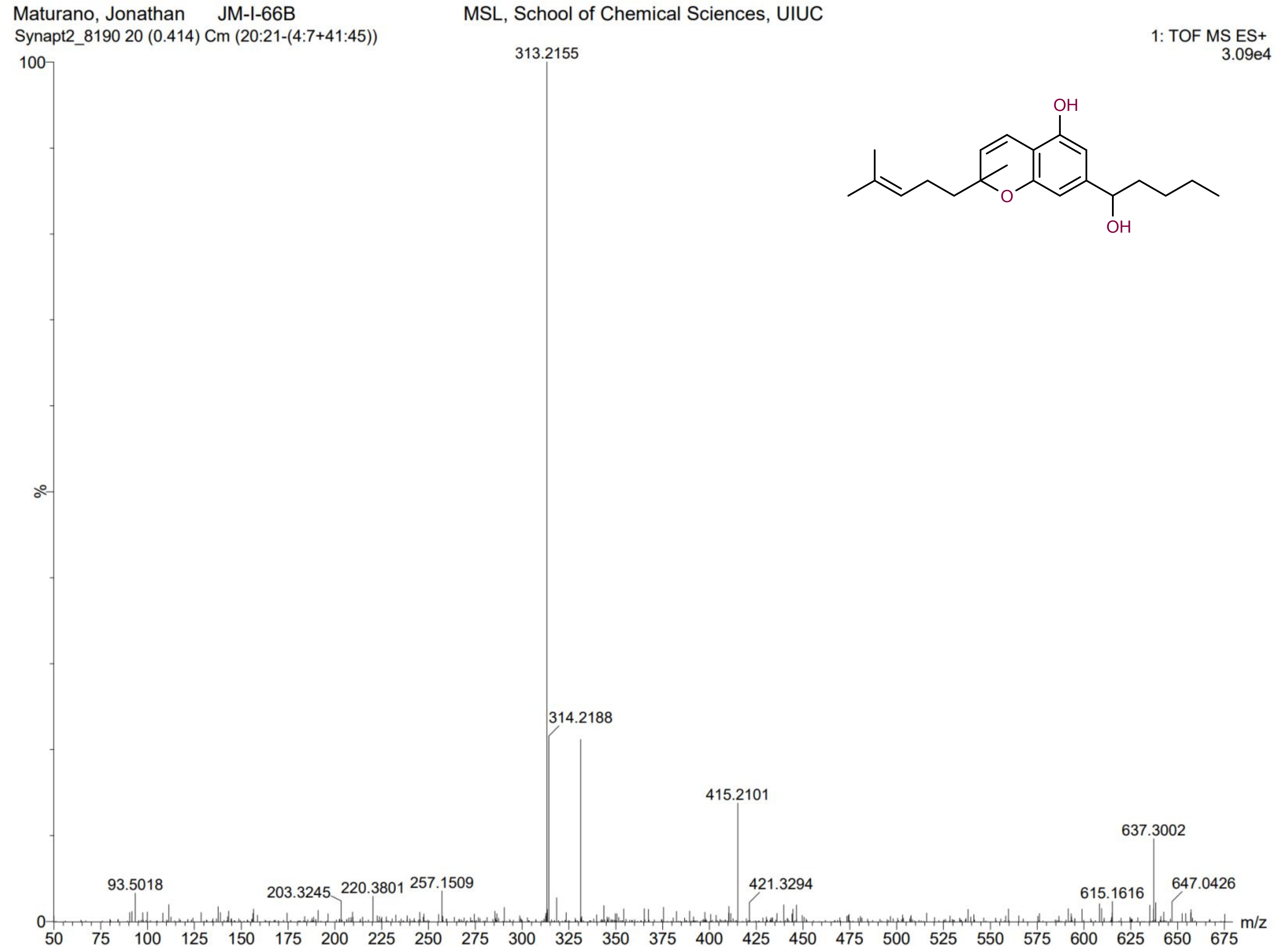

Elemental Composition Report

Page 1

Single Mass Analysis

Tolerance = 5.0 PPM / DBE: min = -1.5, max = 200.0
Element prediction: Off
Number of isotope peaks used for i-FIT = 9

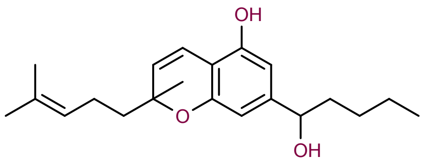

Monoisotopic Mass, Even Electron Ions
176 formula(e) evaluated with 1 results within limits (up to 50 closest results for each mass)
Elements Used:
C: 0-120 H: 0-180 N: 0-5 O: 0-6

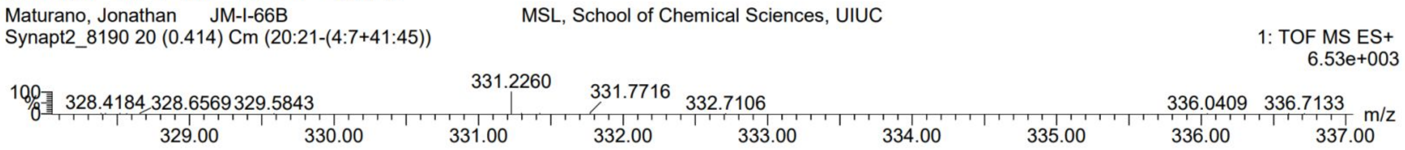

Minimum:				-1.5				
Maximum:		5.0	5.0	200.0				
Mass	Calc. Mass	mDa	PPM	DBE	i-FIT	Norm	Conf(%)	Formula
331.2260	331.2273	-1.3	-3.9	6.5	98.2	n/a	n/a	C21 H31 O3

2. Table of comparison for Binding parameters

Table S1: Binding of CBC to CYPs in absence of CPR

**CBC +**	$\mathbf{A_{max}}$	$\boldsymbol{K}_{\mathbf{d(app)}}$ **(in μM)**
CYP2J2	0.04 ± 0.00	3.06 ± 1.01
CYP2C8	0.15 ± 0.00	38.12 ± 1.28
CYP2D6	0.09 ± 0.01	11.44 ± 0.60
CYP3A4	0.11 ± 0.00	9.57 ± 2.14

Table S2: Binding of CBC to CYPs in presence of CPR

**CBC + CPR+**	$\mathbf{A_{max}}$	$\boldsymbol{K}_{\mathbf{d(app)}}$ **(in μM)**
CYP2J2	0.09 ± 0.00	1.19 ± 0.06
CYP2C8	0.14 ± 0.00	8.23 ± 0.08
CYP2D6	0.04 ± 0.00	3.12 ± 0.16
CYP3A4	0.03 ± 0.00	1.66 ± 0.26

3. Possible fragmentation pattern of CBC and metabolites

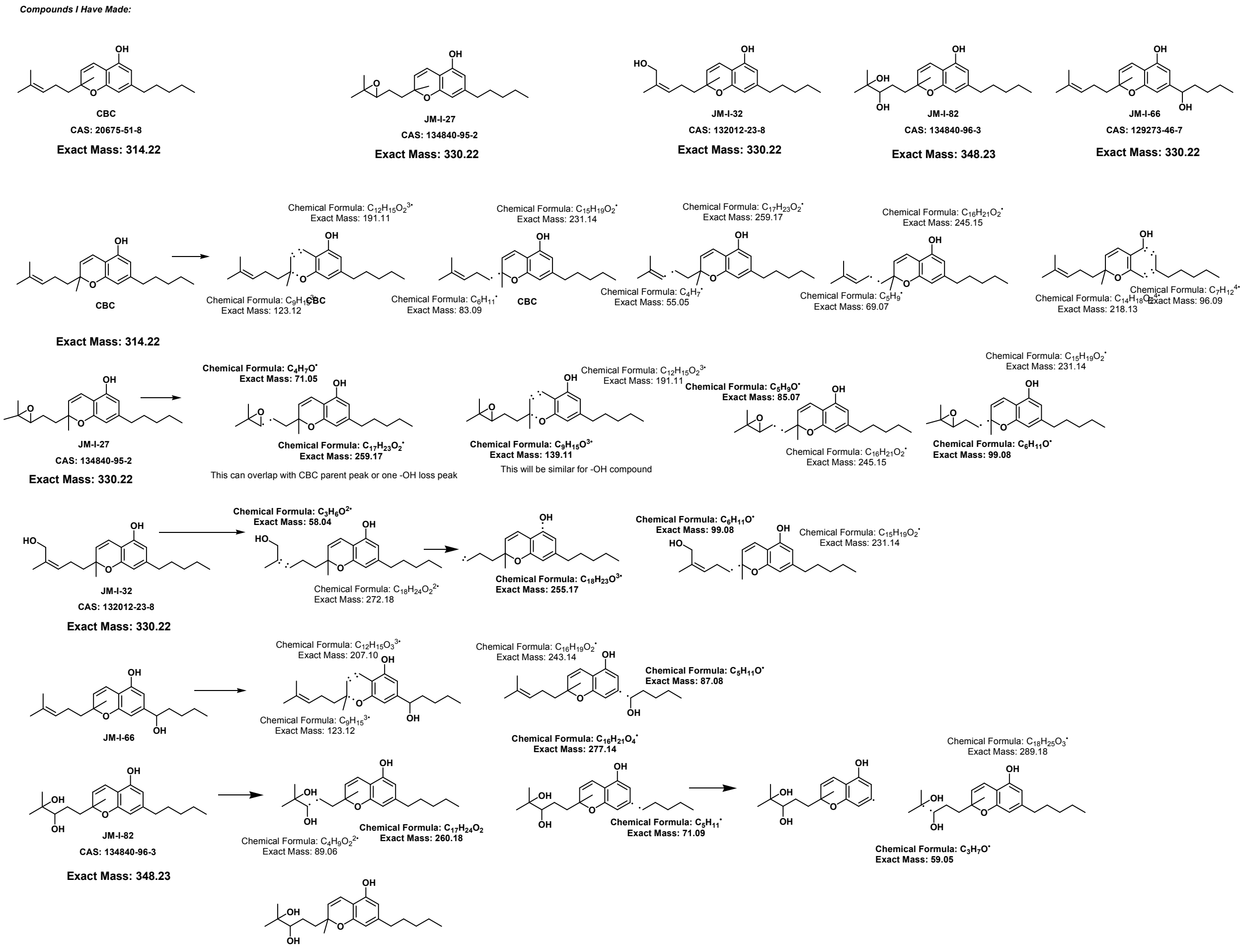

Figure S1: Possible fragments formed from CBC and its metabolites

4. LC-UV/MS of CBC and standard metabolites

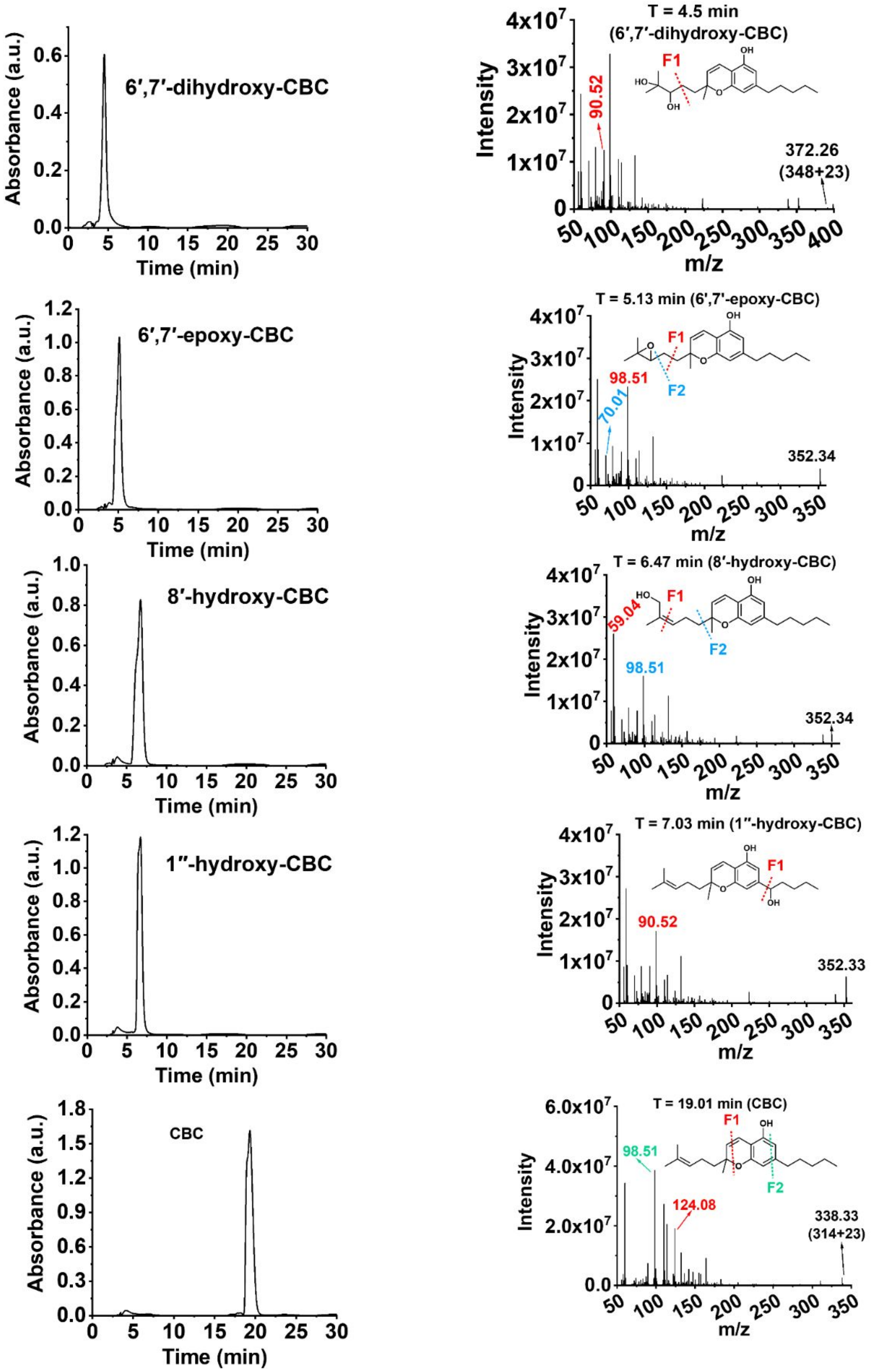

Figure S2: LC-UV chromatogram and corresponding mass fragmentation of CBC and its metabolites

5. LC/UV-MS of CBC metabolites in presence of Human Liver microsome

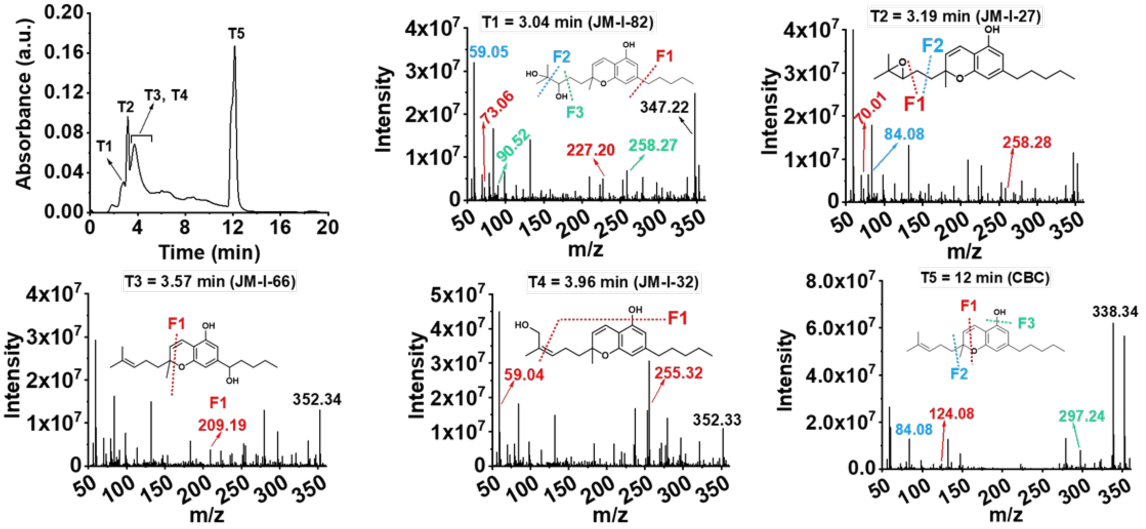

Figure S3: LC-UV chromatogram of CBC metabolites in presence of human liver microsome (HLM)and corresponding mass fragmentation of CBC and its metabolites

6. CBC Metabolism by different CYPs

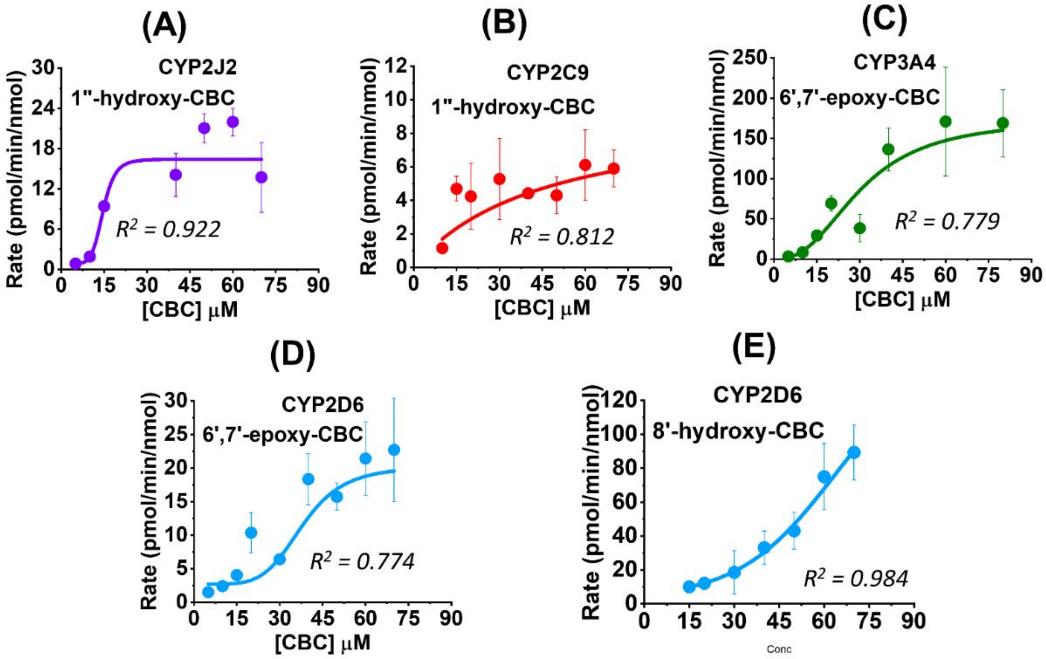

Figure S4. **Metabolism of CBC by different CYPs:** Formation of 1″-hydroxy-CBC by (A) CYP2J2 and (B) CYP2C8; 6′,7′-epoxy-CBC by (C) CYP3A4 and (D) CYP2D6; 8′-hydroxy-CBC by (E) CYP2D6. Data has been fitted to either Michaelis-Menten or Hill equation, error bars represent ±SEM, and R^2 values for fittings are shown.

7. Biological Study

1.5 NO assay, IL-6 assay and MTT Assay

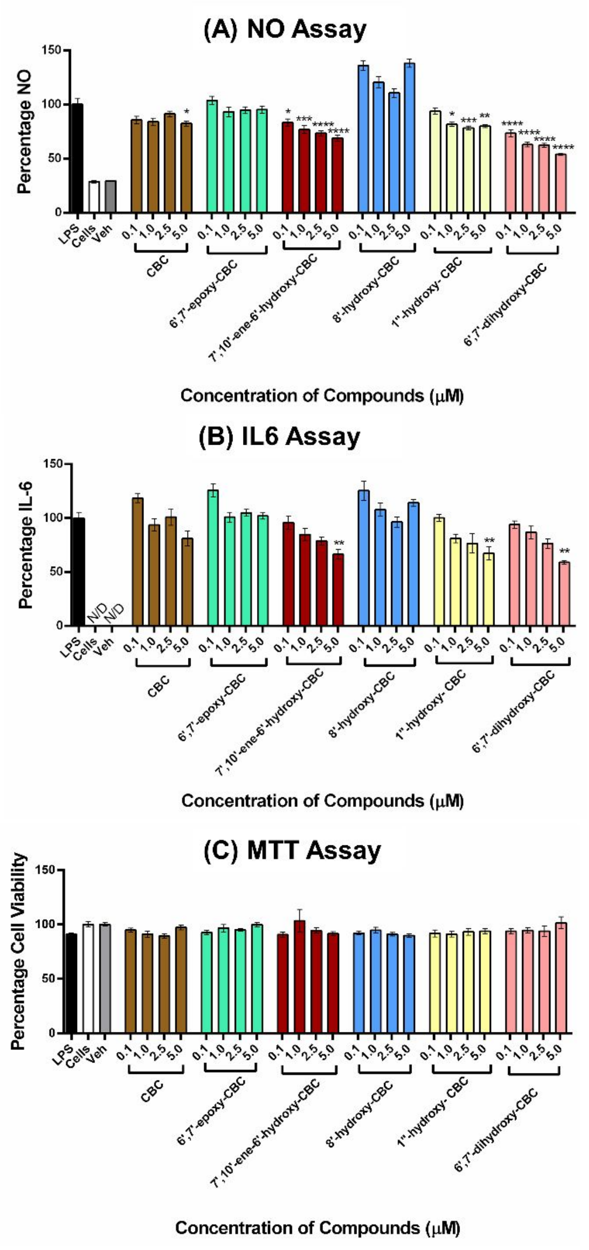

Figure S5: Effect of CBC and its metabolites on biological systems: The BV2 cell assay has been used for the detection of levels of (A) NO released, (B) IL-6 expression, and (C) MTT assay to study the cell viability in presence of CBC and its metabolites. Data are represented as means ±/SE of n=5

1.6 Expression of TNFα and Arginase 1

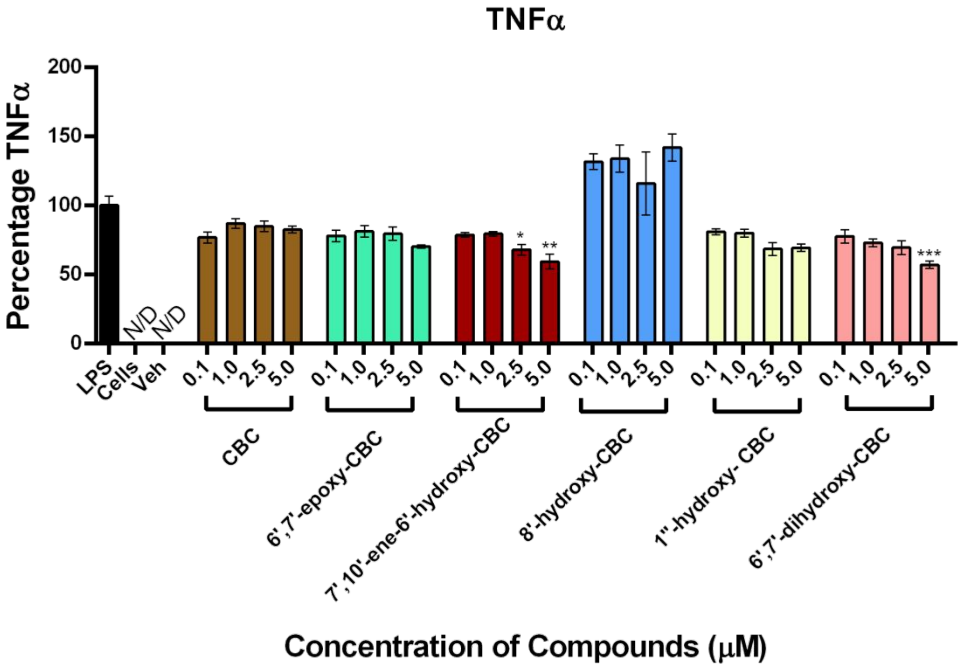

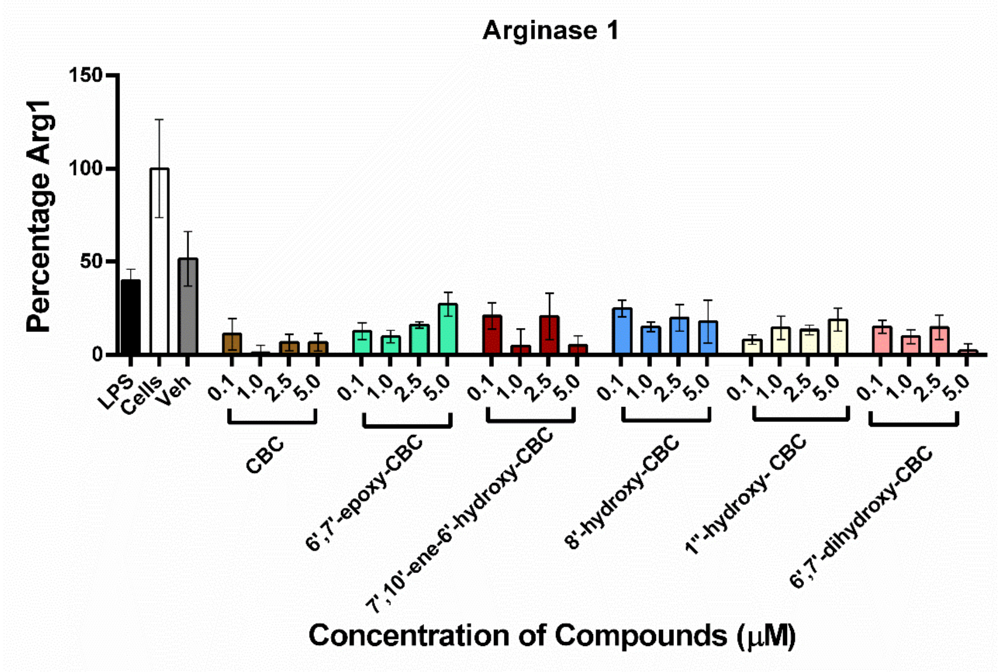

Figure S6: The BV2 cell assay for the detection of levels of TNFα and Agr1 expression. Data are represented as means ±SE of n=5

8. Docking study

1.7 Different orientations of CBC in the active site of CYPs

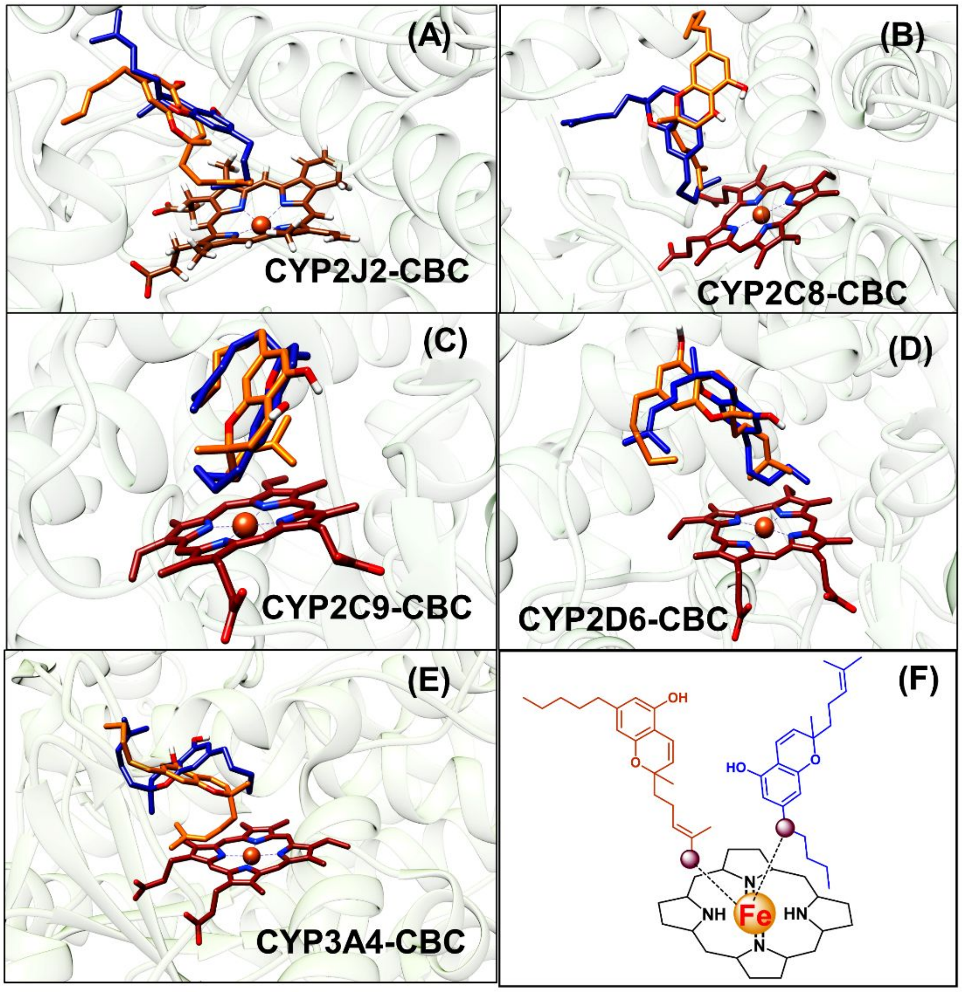

Figure S7. ***Orientations of CBC in the active site***: Docked structure of CBC into CYPs show two different orientations of binding in the active site. Some docked structures have the alkyl chain oriented near the heme (dark blue) whereas in the majority of the docked structure the unsaturated chain is oriented near the heme (orange) (F) CBC attains various conformations while binding with different CYPs. It is somewhat linear in the case of (A) CYP2J2, (B) CYP2C8, and (D) CYP2D6, whereas it attains a bent conformation in (C) CYP2C9 and (E) CYP3A4.

1.8 Active sites around CBC in different docked poses

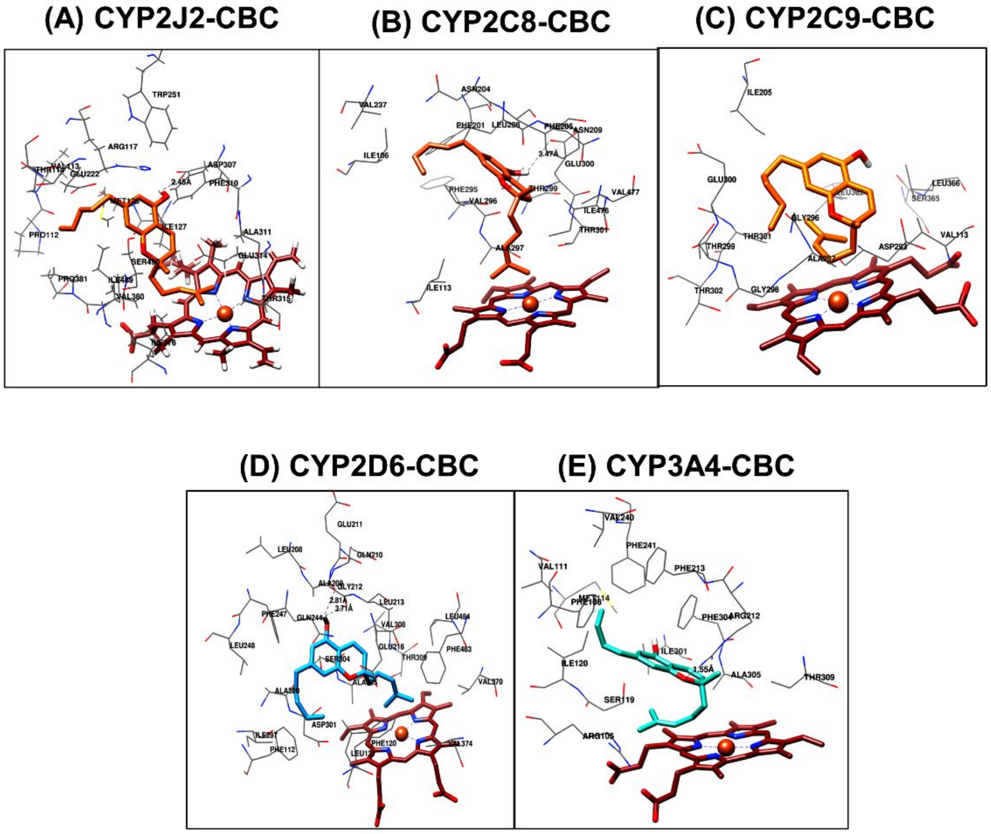

Figure S8. ***CYP active site around different poses of CBC*:** Docked structures of CBC in (A) CYP2J2, (B) CYP2C8, (C) CYP2C9, (D) CYP2D6, and (E) CYP3A4. In all the cases the alkyl chain of CBC faces the heme. The residues within 5 Å of CBC are shown as thin wires. CYP2C9 and CYP3A4 attain a bent conformer whereas the remaining CYPs take a linear structure.

9. Distance of CBC from the heme Fe center in the active site of respective CYPs

Table S3: Distance of 8'C, 6'C and 1"C of CBC from Fe as obtained from the docked structure of CBC with different CYPs

CYPs	8' – Fe	6' – Fe	1″ – Fe
2J2	3.41	4.92	13.71
2C8	4.74	6.6	13.28
2C9	3.5	3.94	8.92
2D6	3.75	5.92	13.21
3A4	6.76	4.61	10.62

10. Molecular Dynamics Simulation of CYP2J2 with CBC

1.9 Distance distributions of selected CBC atoms from the heme Fe center in CYP2J2

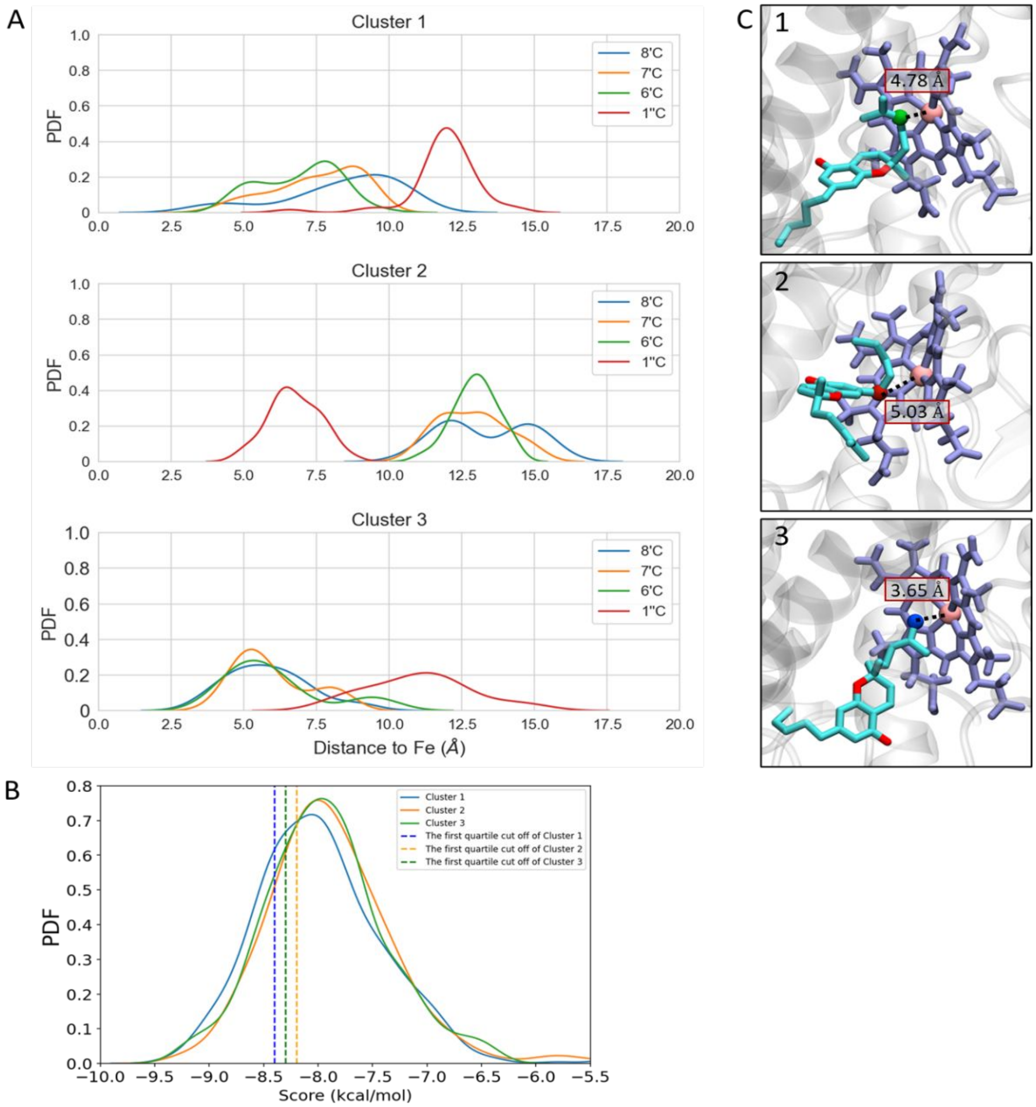

Figure S9: Ensemble molecular docking of CBC in the CYP2J2 active site results in three clusters. (A) The distribution of the selected carbon atoms' distance to the Fe center in each cluster. The distance distributions of the 8´, 7´, 6´ and 1˝ carbon atoms are shown as probability density functions (PDFs) in blue, orange, green, and red, respectively. (B) The distributions of the binding energy scores of the docked poses in each cluster. (C) Representative docked poses for each cluster. Insets 1-3 show the selected pose for clusters 1-3, respectively. The 6´, 1˝ and 8´ carbon atoms are highlighted in green, red, and blue, respectively. The distance between the selected carbon atoms and the Fe atom (pink) is shown as black dashed lines.

1.10 Average RMSD values and contact residues for CBC

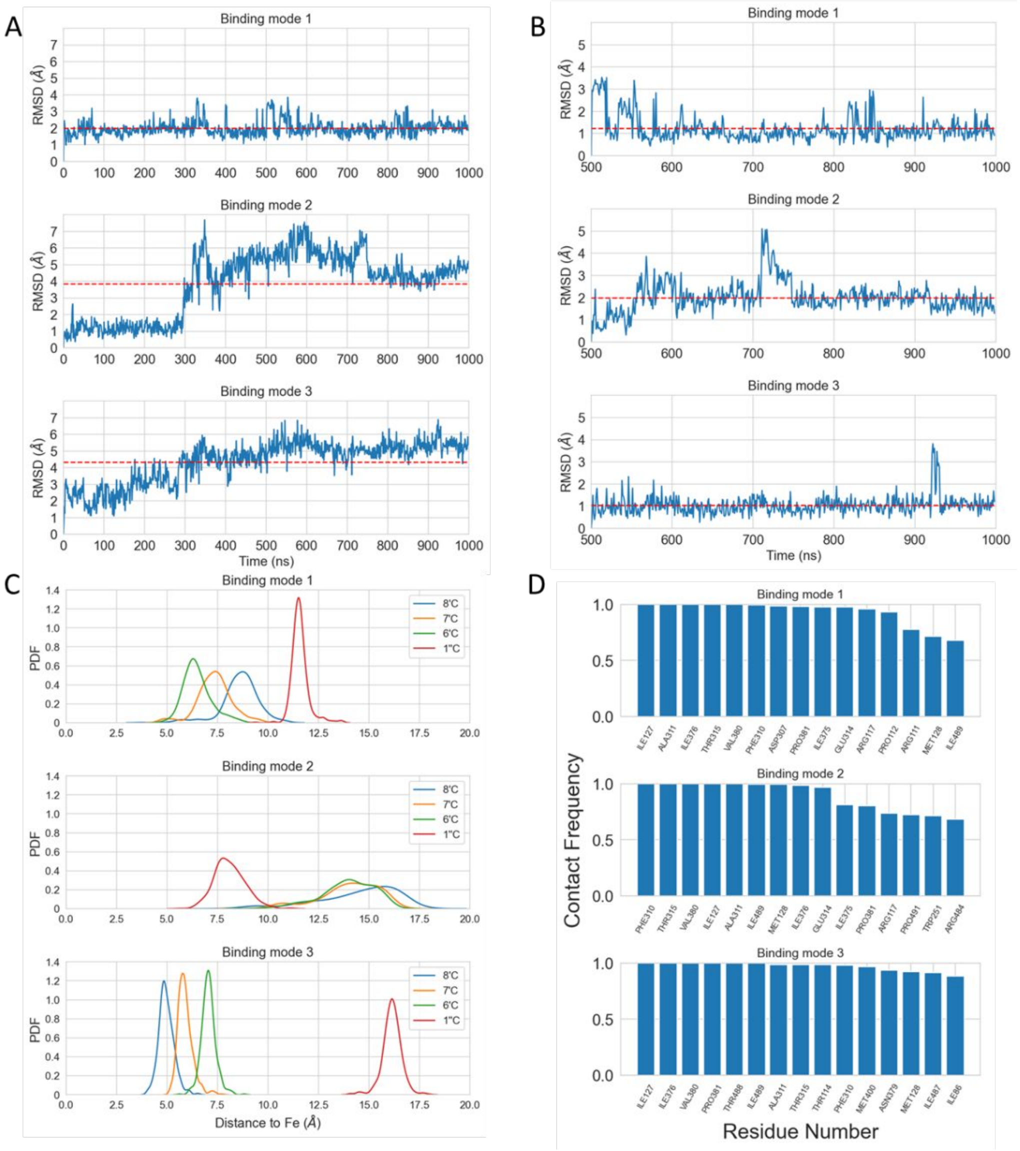

Figure S10: Molecular dynamics simulations of the three binding modes of CBC. (A) RMSD of CBC in each binding mode throughout the simulations. Average RMSD values are shown as red dashed lines. (B) RMSD of each binding mode from the last 500 ns of the simulations. Average RMSD values are shown as red dashed lines. (C) Distance distribution of selected carbon atoms to the Fe center for each binding mode. The heme distance distribution of the 8′, 7′, 6′ and 1″ carbon atoms are shown as probability density functions (PDFs) in blue, orange, green, and red, respectively. (D) The 15 closest (within 4 Å) residues to CBC in each binding mode.

1.11 Representative binding modes and free energy perturbation (FEP) calculations

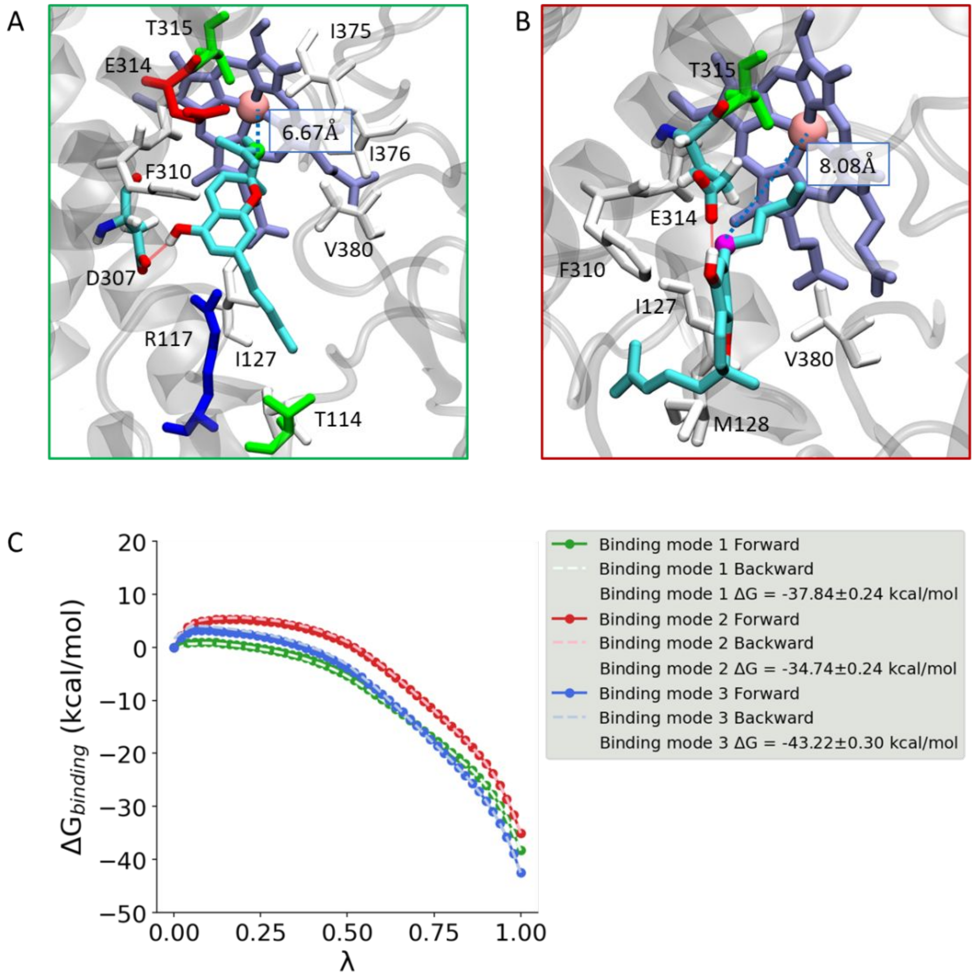

Figure S11. (A) A representative snapshot of binding mode 1 highlights some of the important residues surrounding CBC. Positively charged, negatively charged, polar and hydrophobic residues are colored in blue, red, green, and white, respectively. D307 is shown in cyan (carbon), red (oxygen), and blue (nitrogen). The hydrogen bond between CBC and D307 is shown as a red dashed line. A blue dashed line shows the distance between 6´C (green) and the Fe atom (pink). (B) A representative snapshot of binding mode 2 highlights some of the key residues surrounding CBC. Positively charged, negatively charged, polar, and hydrophobic residues are colored in blue, red, green, and white, respectively. E314 is shown in cyan (carbon), red (oxygen), and blue (nitrogen). The hydrogen bond between CBC and E314 is shown as a red dashed line. The distance between 1″ C (magenta) and the Fe atom (pink) is shown as a blue dashed line. (C) The change in the binding free energy (ΔG) during FEP simulations of the three binding modes. Forward (circles) and backward (dashed line) FEP runs of binding modes 1, 2 and 3 are shown in green, red and blue, respectively, and show convergence of the free energy values.

11. NADPH activity assay

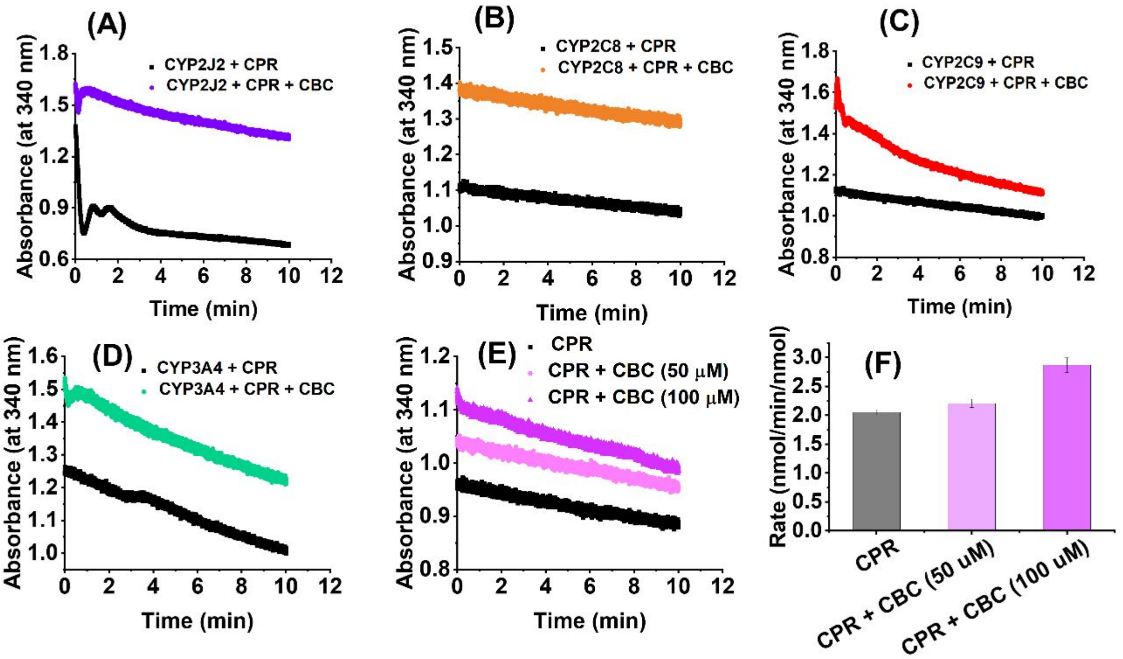

Figure S12. ***NAPDH activity assay***: Rate of change of absorbance of NADPH (at 340 nm) in presence (colored) and absence (black) of CBC (50 μM) for (A) CYP2J2, (B) CYP2C8, (C) CYP2C9, (D) CYP3A4 with CPR as the redox protein. (E) Change in absorbance for CPR without any CYP in the absence and presence of CBC at 50 and 100 μM concentration. Comparison of rate of NADPH consumption for different CYPs are shown in the absence of CBC (grey histogram) and presence of CBC (green histogram); (F) Comparison of rate of NADPH oxidation when CBC is added to CPR in absence of CYPs.

12. NADPH oxidation rates

Table S4: Rates of NADPH oxidation in absence and presence of substrate CBC.

	NADPH rate (CYP+CPR)	NADPH rate (CYP+CPR+CBC)
CYPs	nmol/min/nmol	nmol/min/nmol
2J2	13.34	21.7
2C8	5.81	9.35
2C9	9.99	18.6
2D6	12.29	18.11
3A4	17.49	20.52

13. CYP-CPR docked structure

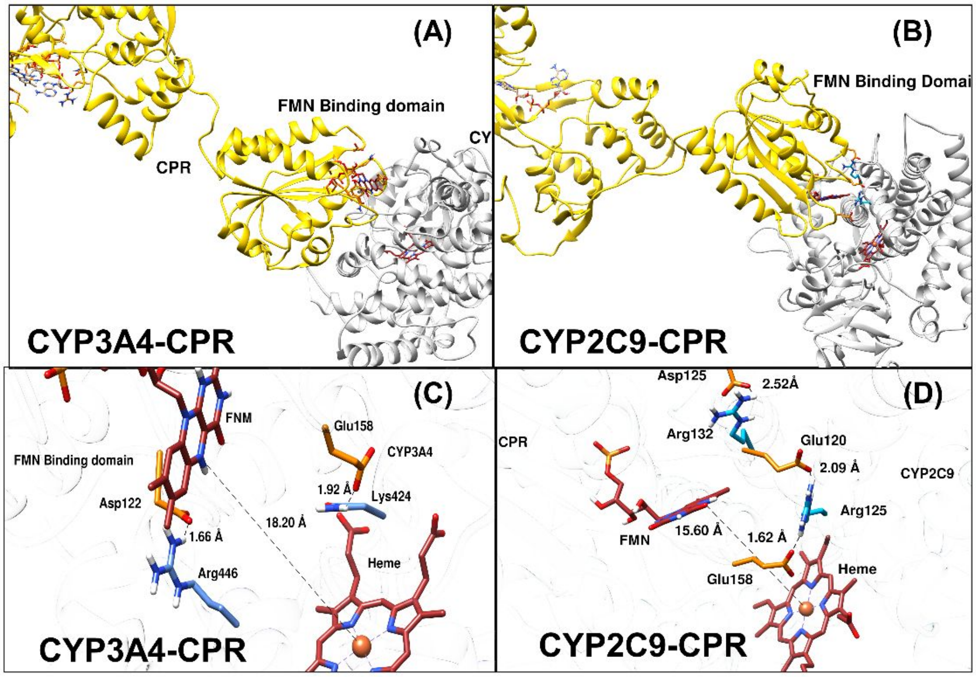

Figure S13. ***Docked structure CPR and CYPs***: Protein-protein docking shows that the FMN domain of CPR interacts with (A) CYP3A4 as well with (B) CYP2C9. The heme distance as well as the H-bonding interactions are shown for (C) CYP3A4 and (D) CYP2C9.

14. Distance between CYP and CPR in the docked structure

Table S5. Distance between CYP3A4 and CPR

CPR	CYP3A4	Distance (Å)
FMN	Fe	18.20
Asp122	Arg446	1.66 (H-bond)
Glu158	Lys424	1.92 (H-bond)

Table S6. Distance between CYP2C9 and CPR

CPR	CYP2C9	Distance (A)
FMN	Fe	15.60
Glu120	Arg125	2.09 (H-bond)
Glu158	Arg125	1.62 (H-bond)
Asp125	Arg132	2.52 (H-bond)

15. Metabolites of CBC docked with CYP-CPR

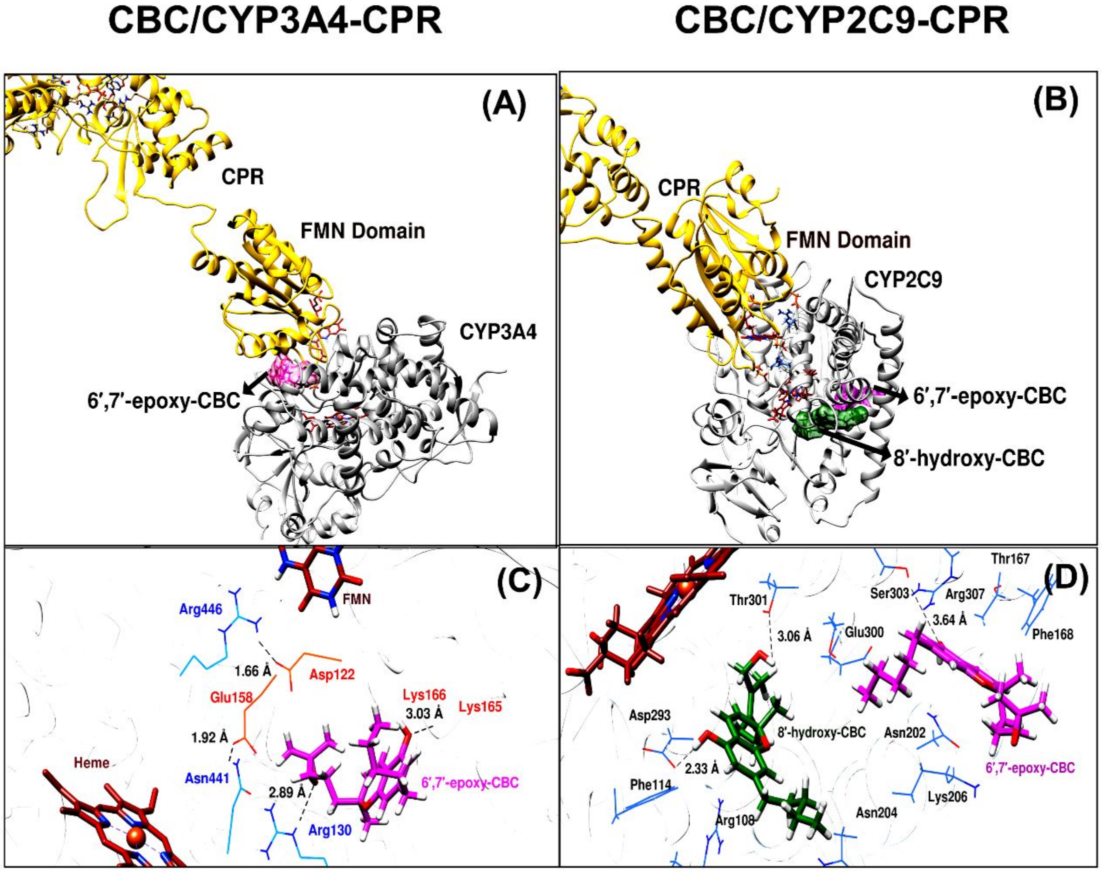

Figure S14. ***Docking studies of CBC metabolites with CYP-CPR complex***: Metabolites of CBC have been docked with CYP3A4-CPR and CYP2C9-CPR complexes to investigate the site of binding. (A) 6′,7′-epoxy-CBC (shown in purple) binds at the interface of CYP3A4 and CPR docked structure; (B) 6′,7′-epoxy-CBC (shown in purple) as well as 8′-hydoxy-CBC (shown in green) is located away from the interface of CYP2C9 and CPR docked structure. **Hydrogen bonding interaction** of (C) 6′,7′-epoxy-CBC (shown in purple) with Arg130 of CYP3A4 and Lys165 CPR; (D) 6′,7′-epoxy-CBC (shown in purple) with Ser303; 8′-hydoxy-CBC (shown in green) with Asp293 and Thr301 of CYP2C9

16. H-bonding interaction of CBC metabolites and CYP-CPR

Table S7. H-bonding distance between CYP3A4-CPR docked structure and 6′,7′-epoxy-CBC

Ligand	CYP3A4-CPR	Distance (Å)
6′,7′-epoxy-CBC (-OH)	N-H of amide (CPR)	3.03
6′,7′-epoxy-CBC (epoxy oxygen)	Arg130 (CYP3A4)	2.89

Table S8. H-bonding distance between CYP2C9-CPR docked structure and 6′,7′-epoxy-CBC as well as 8′-hydoxy-CBC

Ligand	CYP2C9-CPR	Distance (A)
8′-hydoxy-CBC (Allylic –OH)	Thr301	3.06
8′-hydoxy-CBC (ring –OH)	Asp293	2.99
6′,7′-epoxy-CBC (ring –OH)	Ser303	3.64

17. Docking studies of CBC at the active site of different CYPs

The extent of 8′-hydroxy-CBC **(2)** production by CYP2C8 is almost ~5 times more compared to the epoxy product. As shown in Figure S8B, CBC can associate at the active site through hydrogen bonding with Glu300 in helix I (3.47 Å) along with hydrophobic interaction with Phe201 and Phe205 (both in F helix). This docked conformer has C8′ position of CBC closer to the heme which can increases the formation of the 8′-hydroxy product.

Docking studies further show that CBC is associated through two hydrogen bonds at the active site pocket of CYP2D6 (Figure S8D**)**. This might restrict the entry of the substrate into the active site thereby reducing the extent of metabolism. The kinetics plot for CYP2D6 forms a sigmoidal curve which shows that CBC metabolism starts only after the substrate concentration reaches 15-20 μM (Figure 3J**)**. This lower rate of metabolism by CYP2D6 was also observed previously during cannabigerol (CBG) metabolism.[1]

On the contrary, CYP2C9 shows the maximum product formation at the allylic chain. Unlike CYP2J2 and CYP2C8, CYP2C9 is found to promote 6′,7′-epoxidation at double the rate of 8′-hydroxylation. Table S3 (distance comparison from CBC C6′ and heme) indicates that C6′ is closest to the heme for CYP2C9 among all the CYP proteins. Unlike the other docked structures, CBC takes a somewhat folded conformation at the active site of CYP2C9 which aligns the allylic double bond directly over the heme, thereby facilitating epoxide formation. A similar bent structure is also obtained in case for CYP3A4 where the C6′ is located at a distance of 4.6 Å from the heme and C8′ at a distance of 6.76 Å. This can favor the formation of the 6′,7′-epoxide over the 8′-allylic hydroxide. Theoretical calculations on energy minimization during the catalytic

process of CYP3A4 suggest that the epoxidation proceeds through a lower activation energy level compared to the hydroxylation.[2] This can explain the formation of 6′,7′-epoxy-CBC as the only product upon CBC metabolism.

18. Discussion on protein-protein docking

CYPs carry out substrate metabolism only in presence of its redox partner, CPR. CPR has three distinct domains (NADPH, FAD and FMN binding domain) out of which the electron transfer from CPR to CYP occurs through the FMN domain.[3] We aimed to investigate the interactions which stabilizes the CYP-CPR interaction and also to understand whether substrate binding can influence this interaction. We initially carried out CPR-CYP docking studies using the open conformation of CPR and taking CYP2C9 and CYP3A4 as two representative enzymes. Docking studies were carried out using Haddock 2.2 [4,5,6] server in order to find out the most energetically stable CYP-CPR structure. The FMN domain of CPR is rich in acidic amino acids making it negatively charged and it interacts with the proximal region of CYP which has basic residues.[7] As seen from the docked structure (**Fig S13A and S13C; Table S5),** CYP3A4 can associate with CPR through H-bonding interaction between Asp122-Arg446 and Glu158-Lys424 which stabilizes the protein-protein complex. On the other hand such polar interaction is also ovserved in case of CYP2C9 which can associate with CPR through three hydrogen bonds, unlike CYP3A4 which forms two H-bonds (**Figure S13D** and **Table S6**). The distance between the heme atom and the FMN group for CYP3A4-CPR is 18.20 Å and for CYP2C9-CPR it is 15.60 Å. Previously it has been reported that electrostatic interaction plays a key role in stabilizing the CYP-CPR interaction and the distance between heme and FMN fluctuates between 18-20 Å.[8] Smaller heme-FMN distance for CYP2C9-CPR can play a key role in facilitating better electron transport across the protein enabling a higher rate of metabolism.

Substrate for membrane proteins are generally hydrophobic in nature and enters into the active site through the lipid bilayer to which the protein is bound.[8,9] However, the egress channel for the metabolites (which are more hydrophilic as compared to the substrate) consist of a different pathway through which the solvent

enters the active site. Herewin we have investigated whether the metabolites can interact with CYP-CPR complex. For this, we carried out further docking with the CBC metabolites and the CYP-CPR complex. Interestingly the binding site of the metabolite differ from CYP3A4 to CYP2C9. 6′,7′-epoxy-CBC, which is the only metabolite for CYP3A4, binds near the interface of CYP34-CPR and is stabilized by H-bonding interaction through its epoxide part (with Arg130 CYP) and –OH part (amine bond between Lys165-166 in CPR) (**Figure S14A and S14C and Table S7**). This can interfere the electrostatic interaction between the two proteins thereby affecting the electron transport from CPR to CYP. On the other hand for CYP2C9, both 6′,7′-epoxy-CBC and 8′-hydroxy-CBC binds in a similar egress channel as reported previously.[10] This region of the protein is rich in hydrophilic residues which stabilizes both these metabolites through H-bonding in that channel. Interestlingly this region of the protein is situated away from the CYP-CPR interface(**Figure S14B and S14D and Table S8**). From this study it can be concluded that metabolites might play an important role in regulating the electron transfer and enabling substrate metabolism. Unlike CYP3A4, the metabolites does not interfere with the CYP-CPR interaction for CYP2C9. This can be responsible for lower metabolic rate for CYP3A4 in presence of CBC.

19. MD simulation of CYP2J2 and CBC

To study putative binding modes of CBC to CYP2J2, we used ensemble molecular dynamics (MD) and ensemble molecular docking, both of which have been implemented previously to characterize different ligand-CYP2J2 interactions.[11,12] To generate the CYP2J2 conformational dataset for ensemble docking, 200 unique protein conformations were obtained from the last 20 ns of a previously simulated CYP2J2 trajectory.[13] A CBC molecule was docked to the active site using Autodock Vina[14] and the top 10 docked poses for each conformation were collected, resulting in a set of 2000 docked poses. Based on the docked poses and RMSD (root-mean-squared displacement) three different clusters were assigned.

To obtain putative alternate binding modes of CBC leading to the formation of compound (**2**: 8′-hydroxy-CBC), (**4**: 6′,7′-epoxy-CBC) and (**10**: 1″-hydroxy- CBC), the set of docked poses was clustered using RMSD (root-mean-squared displacement) clustering[15] in VMD, resulting in three clusters. Clusters 1, 2, and 3 contain the docked poses with primarily 6′, 1″ and 8′ carbon atoms closest to the Fe atom, respectively (Figure S9A). Clusters 1, 2 and 3 may represent different binding modes of CBC leading to the production of compound 6′,7′-epoxy-CBC, 1″-hydroxy- CBC and 8′-hydoxy-CBC, respectively. The distribution of the binding energy scores of docked poses in each cluster is shown in Figure S9B. The docked pose with the most favorable binding energy score in the first quartile of the distribution was chosen as the representative binding mode for each cluster (Figure S9C). To further analyze the stability of such representative binding modes using MD simulations, each binding mode was simulated for an additional 1 μs.

After 500 ns, the RMSD of each binding mode is within 2 Å, indicating that they are stabilized in the active site cavity (Figure 5A, S10 A-B). Furthermore, binding mode 3 shows the 8′ carbon atom closest to the Fe atom among all binding modes with an average distance of 4.97 Å (Figure 5B, S10 C). This is in correlation with the experimental findings since 8′-hydoxy-CBC is formed as the major CBC metabolite in CYP2J2. Key amino acid residues interacting with CBC in binding mode 3 are highlighted in Figure 5D. Furthermore, our data suggests consistent hydrogen bonding, mainly between the CBC hydroxy group and the backbone oxygen of I487, as well as N379, mediated by a water molecule, potentially stabilizing this binding mode (Figure 5D). The 15 strongest interacting residues with each binding mode and representative snapshots of binding modes 1 and 2 are shown in Figure S10 D, S11 A-B. Moreover, absolute free energy perturbation (FEP) calculations were used to compare relative binding affinities of the regioselective binding modes (Figure 5 E and S11 C). The lowest relative binding free energy was observed in binding

mode 3, indicative of the highest binding affinity among all binding modes. The high binding affinity of binding mode 3 is likely due to contributions from the stabilizing hydrogen bonding network and the surrounding hydrophobic residues in the enzyme cavity (Figure 5 D). Collectively, our results suggest that CBC is stabilized in the active site preferably with its 8′C closest to the heme group, which primes selective production of compound (**2:** 8′-hydroxy-CBC) in CYP2J2.

20. References

(1) Rowland, P.; Blaney, F. E.; Smyth, M. G.; Jones, J. J.; Leydon, V. R.; Oxbrow, A. K.; Lewis, C. J.; Tennant, M. G.; Modi, S.; Eggleston, D. S.; et al. *Journal of Biological Chemistry* **2006**, *281* (11), 7614-7622.
(2) Hata, M.; Tanaka, Y.; Kyoda, N.; Osakabe, T.; Yuki, H.; Ishii, I.; Kitada, M.; Neya, S.; Hoshino, T. *Bioorganic & Medicinal Chemistry* **2008**, *16* (9), 5134-5148.
(3) Mukherjee, G.; Nandekar, P. P.; Wade, R. C. *Communications Biology* **2021**, *4* (1), 55.
(4) Dominguez, C.; Boelens, R.; Bonvin, A. M. J. J. *Journal of the American Chemical Society* **2003**, *125* (7), 1731-1737.
(5) van Zundert, G. C. P.; Rodrigues, J. P. G. L. M.; Trellet, M.; Schmitz, C.; Kastritis, P. L.; Karaca, E.; Melquiond, A. S. J.; van Dijk, M.; de Vries, S. J.; Bonvin, A. M. J. J. *Journal of Molecular Biology* **2016**, *428* (4), 720-725.
(6) Honorato, R. V.; Koukos, P. I.; Jiménez-García, B.; Tsaregorodtsev, A.; Verlato, M.; Giachetti, A.; Rosato, A.; Bonvin, A. M. J. J. *Frontiers in Molecular Biosciences* **2021**, *8*.
(7) Esteves, F.; Campelo, D.; Gomes, B. C.; Urban, P.; Bozonnet, S.; Lautier, T.; Rueff, J.; Truan, G.; Kranendonk, M. *Frontiers in Pharmacology* **2020**, *11*.
(8) Šrejber, M.; Navrátilová, V.; Paloncýová, M.; Bazgier, V.; Berka, K.; Anzenbacher, P.; Otyepka, M. *Journal of Inorganic Biochemistry* **2018**, *183*, 117-136.
(9) Szlenk, C. T.; Gc, J. B.; Natesan, S. *Molecular Pharmacology* **2019**, *96* (5), 527.
(10) Berka, K.; Hendrychová, T.; Anzenbacher, P.; Otyepka, M. *The Journal of Physical Chemistry A* **2011**, *115* (41), 11248-11255.
(11) Carnevale, L. N.; Arango, A. S.; Arnold, W. R.; Tajkhorshid, E.; Das, A. *Biochemistry* **2018**, *57* (46), 6489-6499.
(12) Kim, J. S.; Arango, A. S.; Shah, S.; Arnold, W. R.; Tajkhorshid, E.; Das, A. *Journal of Inorganic Biochemistry* **2022**, *229*, 111722.
(13) McDougle, D. R.; Baylon, J. L.; Meling, D. D.; Kambalyal, A.; Grinkova, Y. V.; Hammernik, J.; Tajkhorshid, E.; Das, A. *Biochimica et Biophysica Acta (BBA) - Biomembranes* **2015**, *1848* (10, Part A), 2460-2470.
(14) Trott, O.; Olson, A. J. *Journal of Computational Chemistry* **2010**, *31* (2), 455-461.
(15) Heyer, L. J.; Kruglyak S Fau - Yooseph, S.; Yooseph, S. *Genome Research* **1999**, *9* (11), 1106-1115.
